# ATAD3A oligomerization causes neurodegeneration by coupling mitochondrial fragmentation and bioenergetics defects

**DOI:** 10.1038/s41467-019-09291-x

**Published:** 2019-03-26

**Authors:** Yuanyuan Zhao, Xiaoyan Sun, Di Hu, Domenick A. Prosdocimo, Charles Hoppel, Mukesh K. Jain, Rajesh Ramachandran, Xin Qi

**Affiliations:** 1 Department of Physiology and Biophysics, Case Western Reserve University School of Medicine, Cleveland, OH 44106 USA; 2 Case Cardiovascular Research Institute and Harrington Heart and Vascular Institute, Case Western Reserve University School of Medicine, Cleveland, OH 44106 USA; 3 Department of Medicine, University Hospitals Case Medical Center, Case Western Reserve University School of Medicine, Cleveland, OH 44106 USA; 4 Center for Mitochondrial Disease, Case Western Reserve University School of Medicine, Cleveland, OH 44106 USA; 5 Department of Pharmacology, Case Western Reserve University School of Medicine, Cleveland, OH 44106 USA

## Abstract

Mitochondrial fragmentation and bioenergetic failure manifest in Huntington’s disease (HD), a fatal neurodegenerative disease. The factors that couple mitochondrial fusion/fission with bioenergetics and their impacts on neurodegeneration however remain poorly understood. Our proteomic analysis identifies mitochondrial protein ATAD3A as an interactor of mitochondrial fission GTPase, Drp1, in HD. Here we show that, in HD, ATAD3A dimerization due to deacetylation at K135 residue is required for Drp1-mediated mitochondrial fragmentation. Disturbance of ATAD3A steady state impairs mtDNA maintenance by disrupting TFAM/mtDNA binding. Blocking Drp1/ATAD3A interaction with a peptide, DA1, abolishes ATAD3A oligomerization, suppresses mitochondrial fragmentation and mtDNA lesion, and reduces bioenergetic deficits and cell death in HD mouse- and patient-derived cells. DA1 treatment reduces behavioral and neuropathological phenotypes in HD transgenic mice. Our findings demonstrate that ATAD3A plays a key role in neurodegeneration by linking Drp1-induced mitochondrial fragmentation to defective mtDNA maintenance, suggesting that DA1 might be useful for developing HD therapeutics.

## Abstract (web summary)

Huntington’s disease leads to mitochondrial fragmentation and bioenergetic failure, although how the two events are connected is poorly understood. Here, Zhao et al. identify ATAD3A as a molecular linker and show that a peptide inhibitor of ATAD3A oligomerization suppresses HD phenotypes.

## Introduction

Defects in mitochondrial fusion/fission and mitochondrial bioenergetics have been implicated in the pathogenesis of many neurodegenerative diseases, such as Alzheimer’s, Parkinson’s, and Huntington’s diseases. Dynamin-related protein 1 (Drp1), a cytosolic GTPase, mediates mitochondrial fission. Upon activation, Drp1 is recruited from the cytosol to the surface of mitochondria, where it assembles by self-oligomerization to initiate mitochondrial division^1^. Mitochondrial nucleoids, which contain mitochondrial DNA (mtDNA)-protein complex, facilitate mtDNA maintenance and gene expression and are essential for mitochondrial biogenesis and cellular energy homeostasis^2^. Recent studies suggest that the distribution and organization of mitochondrial nucleoids are associated with mitochondrial division. Mitochondrial nucleoids are found adjacent to Drp1 at the tips of newly divided mitochondria^3^. Knockout of Drp1 causes severe mtDNA nucleoid clustering, which leads to mitochondrial respiration deficiency in both cultured cells and mouse hearts^4,5^. Despite these observations, the factors that couple mitochondrial dynamics with mtDNA maintenance and bioenergetics remain poorly understood. In particular, it is unclear how dysregulation in the signaling pathways involved in mitochondrial fission impacts mtDNA integrity in the context of neurodegeneration.

Huntington’s disease (HD) is a fatal inherited neurodegenerative disease caused by an expansion of a polyglutamine repeat within the protein huntingtin (Htt)^6^. Drp1 hyperactivation and mitochondrial fission impairment occur in various HD models^7–9^. We previously reported that inhibition of Drp1 hyperactivation is sufficient to reduce HD-associated neuropathology^7^, underscoring the importance of Drp1-mediated mitochondrial damage in HD pathogenesis. Crucial questions, such as how Drp1 hyper-activation mediates mitochondrial dysregulation, especially the damage that occurs inside mitochondria, and how these processes are linked to neurodegeneration, however, remain to be answered.

ATAD3A (ATPase family AAA-domain containing protein 3 A) is a nuclear-encoded mitochondrial protein that spans the inner and outer membranes with its two terminal domains located in the outer membrane and the matrix^10–12^. ATAD3A regulates mitochondrial morphology and controls cholesterol trafficking at mitochondrial contact sites^10,13^. Either overexpression^14,15^ or downregulation of ATAD3A^10,16^ results in mitochondrial fragmentation, suggesting a scaffold-like property on maintenance of mitochondrial morphology. Moreover, ATAD3A is a component of mitochondrial nucleoid complex, which implicates in mtDNA nucleoid maintenance^17^. While global knockout of ATAD3A is embryonic lethal^18^, selective loss of ATAD3A in mouse skeletal muscle disrupts mitochondrial ultrastructure and reduces the number of cristae junctions, which impairs mtDNA integrity^19^. The expression of mutant ATAD3A in Drosophila causes severe mitochondrial fragmentation, aberrant cristae, and increased mitophagy in both motor neurons and muscle, leading to early lethality^20^. Patients carrying a ATAD3A mutant show neurodegenerative conditions associated with axonal neuropathy^20^, and spastic paraplegia^14^. The proper function of ATAD3A is therefore critical for cell survival.

In the current study, using unbiased proteomics for Drp1-interacting proteins in neuronal cells derived from HD patient induced pluripotent stem cells (HD-iPS cells), we identify ATAD3A as a candidate interactor. We show that in HD, ATAD3A forms oligomers which bridge Drp1-mediated mitochondrial fragmentation and mtDNA instability, leading to impaired mitochondrial biogenesis and neurodegeneration. We demonstrate that suppression of Drp1/ATAD3A binding by a peptide inhibitor DA1 is protective in various model of HD in vitro and in vivo.

## Results

### ATAD3A is a binding protein of Drp1 in HD

**Fig. 1:**
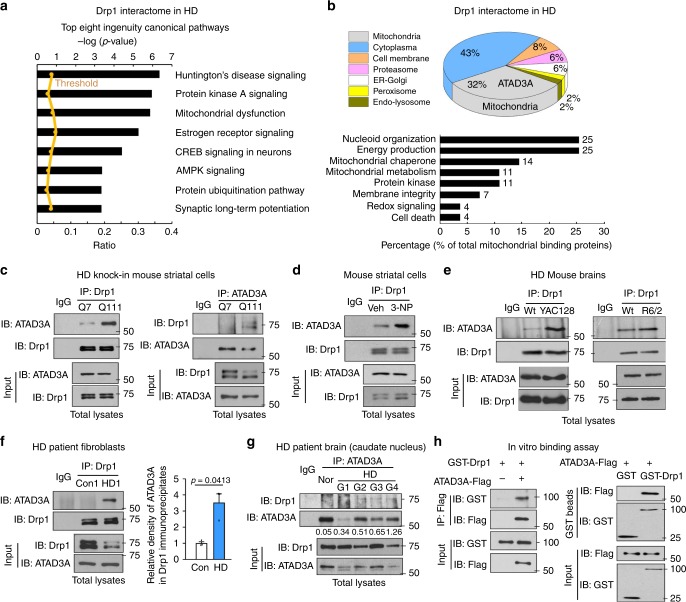
ATAD3A binds to Drp1 in HD. **a** Affinity purification followed by tandem mass spectrometry analysis was conducted to identify Drp1-interacting  proteins in striatal neuronal cells derived from HD patient-iPS or normal subject-iPS cells (also see Supplementary Fig. 1A). The molecular and cellular functions of the Drp1 interactors in HD patient-derived neuronal cells are shown. **b** Upper: cellular location of the Drp1 interactors in HD neuronal cells. In particular, 32% of proteins are located on the mitochondria. ATAD3A ranked as a top candidate. Lower: Among mitochondrial candidates, mitochondrial nucleoid organization and energy production are enriched, and each account for up to 25% of all mitochondrial candidates. Immunoprecipitation (IP) of total protein lysates was performed in HdhQ7 and HdhQ111 cells (**c**), in wildtype striatal cells exposed to 3-NP (5 mM for 4 h) (**d**), in striatal extracts of YAC128 mice (6 months old) or R6/2 mice (12 weeks old) or age-matched wildtype mice (**e**), and in fibroblasts of HD patient (HD1, GM4208, 35 years old, Male) or normal subject (Con1, Huf1) (**f**). Data are mean ± SEM. Student *t* test. *n* = 3. **g** Total protein lysates of frozen postmortem caudate nucleus of normal subjects and HD patients with different disease severity (grade 1 to grade 4, slight to severe) were subject to IP analysis. Nor: normal subject 5214; G1: HD 4283, grade 1; G2: HD 4557, grade 2; G3: HD 3573, grade 3; G4: HD 2903, grade 4. Patient information shown in Supplementary Fig. 1F. The data below the blots indicate the relative density of Drp1 in ATAD3A immunoprecipitates (Drp1/ATAD3A in IP analysis). **h** GST-Drp1 (2 µg) and ATAD3A-Flag (2 µg) purified proteins were incubated in vitro for 30 mins. Left: IP with anti-Flag antibodies followed by western blot with the indicated antibodies. Right: GST pull down analysis. All shown representative blots are from at least 3 independent experiments

Using unbiased proteomic analysis, we set out to identify protein candidates that interact with Drp1 in striatal neurons derived from HD patient-iPS cells (Supplementary Fig. 1A). Tandem mass spectrometry analysis following affinity purification identified 91 proteins that putatively bound to Drp1 in HD patient cells but not in cells derived from normal subject-iPS cells (Supplementary Fig. 1B). These proteins are involved in multiple pathways of cellular functions (Fig. 1a). Focusing on the protein candidates located on mitochondria, we detected enrichment of the proteins involved in mitochondrial nucleoid organization and energy production (Fig. 1b, Table 1). Among these candidates, ATAD3A, a component of the mitochondrial nucleoid complex^17^, ranked as the top candidate for Drp1 binding in HD neuronal cells (Fig. 1b, Table 1).

**Table 1:** A list of mitochondrial candidates that bind to Drp1 in neuronal cells derived from HD patient-iPS cells and not in cells from normal subjects

Function group	Uniprot ID	Protein name	Symbol	Spec count
Mitochondrial nucleiod assembly	Q9NVI7	ATPase family AAA domain-containing protein 3A	ATAD3A	16
	Q96EY7	Pentatricopeptide repeat-containing protein 3, mitochondrial	PTCD3	16
	P19388	DNA-directed RNA polymerases I, II, and III subunit RPABC1	POLR2E	12
	Q96EY1	DnaJ homolog subfamily A member 3, mitochondrial	DNAJA3	9
	Q00059	Transcription factor A, mitochondrial	TFAM	4
	Q07021	Complement component 1 Q subcomponent-binding protein, mitochondrial	C1QBP	7
	P55084	Trifunctional enzyme subunit beta, mitochondrial	HADHB	5
Energy production	O75947	ATP synthase subunit d, mitochondrial	ATP5H	13
	P14618	Pyruvate kinase isozymes M1/M2	PKM2	12
	P24539	ATP synthase subunit b, mitochondrial	ATP5F1	6
	P36542	ATP synthase subunit gamma, mitochondrial	ATP5C1	5
	P22695	Cytochrome b-c1 complex subunit 2, mitochondrial	UQCRC2	5
	O75964	ATP synthase subunit g, mitochondrial	ATP5L	5
	P56134	ATP synthase subunit f, mitochondrial	ATP5J2	4
Mitochondrial chaperone	P38646	Stress-70 protein, mitochondrial	HSPA9	36
	P08107	Heat shock 70 kDa protein 1 A/1B	HSPA1A	19
	P54652	Heat shock-related 70 kDa protein 2	HSPA2	14
	P34931	Heat shock 70 kDa protein 1-like	HSPA1L	9
Mitochondrial metabolism	P36957	Dihydrolipoyllysine-residue succinyltransferase component of 2-oxoglutarate dehydrogenase complex, mitochondrial	DLST	14
	O14874	[3-methyl-2-oxobutanoate dehydrogenase [lipoamide]] kinase, mitochondrial	BCKDK	11
	P11182	Lipoamide acyltransferase component of branched-chain alpha-keto acid dehydrogenase complex, mitochondrial	DBT	5
Protein kinase	P67775	Serine/threonine-protein phosphatase 2 A catalytic subunit alpha isoform	PPP2CA	11
	P12532	Creatine kinase U-type, mitochondrial	CKMT1A	11
	P28482	Mitogen-activated protein kinase 1	MAPK1	11
Mitochodnrial membrane integrity	P05141	ADP/ATP translocase 2	SLC25A5	11
	P45880	Voltage-dependent anion-selective channel protein 2	VDAC2	6
Redox signaling	P30048	Thioredoxin-dependent peroxide reductase, mitochondrial	PRDX3	5
Apoptotic signaling	Q8WUZ0	B-cell CLL/lymphoma 7 protein family member C	BCL7C	4

Immunoprecipitation (IP) analysis demonstrated a greater interaction between Drp1 and ATAD3A in HdhQ111 cells than in HdhQ7 cells (Fig. 1c). In wildtype mouse striatal cells, 3-nitropropionic acid (3-NP), a neurotoxin causing HD-like symptoms in rodents and primates^21^, induced an interaction between Drp1 and ATAD3A (Fig. 1d). ATAD3A preferentially bound to Drp1 in striatal protein lysates of HD YAC128 and R6/2 mice relative to those of wildtype mice (Fig. 1e). Consistently, increased binding of Drp1/ATAD3A was observed in HD patient fibroblasts (Fig. 1f, Supplementary Fig. 1C) and HD patient postmortem brains (Fig. 1g, Supplementary Fig. 1D, 1E), suggesting a role of the interaction in human HD.

In vitro protein binding assay showed that GST-Drp1 and ATAD3A-Flag protein interacted (Fig. 1h). To map the domain interaction between ATAD3A and Drp1, we constructed several truncated mutants of ATAD3A, including ATAD3A ΔN50, which lacks the first 50 amino acids of the N-terminus; ATAD3A ΔCC, in which the two coiled-coil (CC) domains of ATAD3A are deleted; and ATAD3A ΔN245, which lacks N-terminal 245 amino acids and only contains a part of TM domain and ATPase domain (Supplementary Fig. 1G). By IP analysis, we demonstrated that ATAD3A CC domain bound to Drp1 GTPase domain (Supplementary Fig. 1G).

Collectively, our data demonstrate that Drp1 directly binds to ATAD3A and that the binding is elevated in HD.

### Oligomerization of ATAD3A increases in HD

**Fig. 2:**
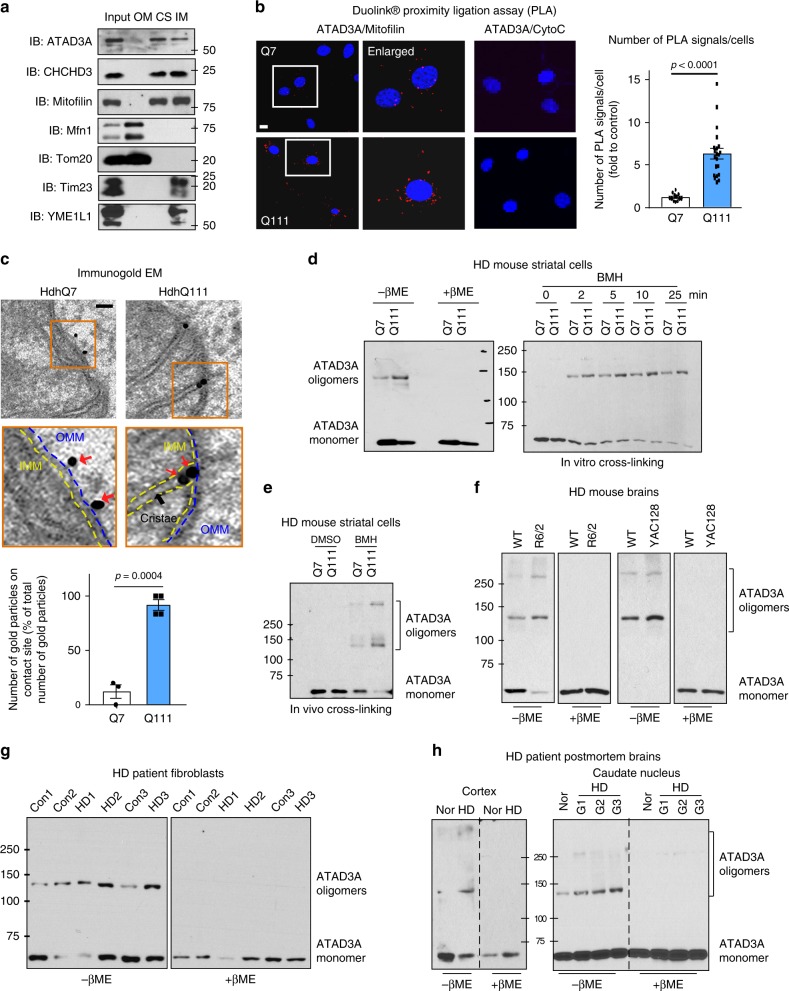
Enhanced ATAD3A oligomerization in HD models. **a** Western blot (WB) analysis of rat liver mitochondrial fractionations with the indicated mitochondrial antibodies. OM: mitochondrial outer membrane. CS: contact sites. IM: mitochondrial inner membrane. **b** HdhQ7 and HdhQ111 cells were stained with anti-ATAD3A and anti-Mitofilin (a mitochondrial contact site protein) or anti-Cyto *C* (a mitochondrial intermembrane space protein) antibodies. Nuclei were stained with DAPI. In situ Duolink proximity ligation assay (PLA) was performed. Histogram: quantitation of the number of PLA-positive signals (red). At least 200 cells/group were analyzed. 3 independent experiments, unpaired Student *t*-test. Scale bar: 10 µm. **c** Immunogold electron microscopy analysis with an antibody directed against ATAD3A N-terminus was performed in HdhQ7 and HdhQ111 cells. Bottom images are the images boxed. Scale bar: 100 nm. Blue dash line: OMM, mitochondrial outer membrane. Yellow dash line: IMM, mitochondrial inner membrane. Red arrowhead: ATAD3A. Histogram: the percentage of ATAD3A immunopositive gold particles on the contact site versus total number of gold particles. 3 independent experiments, unpaired Student *t*-test. **d** Left: ATAD3A protein level was determined by WB in the presence or absence of β-ME. Right: Protein lysates were incubated with the crosslinker BMH (1 mM). **e** HdhQ7 and Q111 cells were treated with BMH (1 mM, 20 min). **f** Total lysates of striatum from YAC128 (12 months old), R6/2 (12 weeks old), or age-matched wildtype mice were analyzed by WB with anti-ATAD3A antibody in the presence or absence of β-ME. *n* = 6–10 mice/group. **g** ATAD3A oligomers were analyzed in HD patient fibroblasts (HD1: GM21756, Female; HD2: GM04693, Male; HD3: GM21756, Female) and normal subjects (Con 1: nHDF, fibroblasts from juvenile; Con 2: HDF, fibroblasts from adult; Con 3: Huf1822, adult). **h** ATAD3A oligomers were analyzed in total lysates of HD patient postmortem brains (cortex: left; caudate nucleus: right) or normal subjects, under non-reducing condition. [Cortex: Normal X5302; HD X5298. Caudate nucleus: Normal 5214; HD Grade1 (G1): 4283; HD Grade2 (G2): 4557; HD Grade3 (G3): 3573]. See Supplementary Fig. 1F for patient information. The data are mean ± SEM. Shown representative blots are from at least 3 independent experiments

Western blot analysis of rat liver mitochondrial sub-compartmental fractionations revealed that ATAD3A was located on both contact sites and the inner membrane (IMM) (Fig. 2a). CHCHD3 and Mitofilin, two proteins located on the contact sites^22^, were found in the same fractionations as ATAD3A (Fig. 2a). To quantify the mitochondrial sub-compartmental location of ATAD3A in HD, we applied in situ Duolink proximity ligation assay (PLA)^23^ which allows us to examine the endogenous ATAD3A location by determining the proximity between ATAD3A and the markers of mitochondrial sub-compartments. We observed a more than 5-fold increase in the PLA positive signals in HdhQ111 cells relative to HdhQ7 cells after staining the cells with anti-ATAD3A and anti-mitofilin (a mitochondrial contact site marker) antibodies (Fig. 2b). PLA positive signal was observed in neither HdhQ7 nor HdhQ111 cells stained with anti-ATAD3A and anti-cytochrome *c* (a mitochondrial intermembrane space protein) (Fig. 2b). Immunogold electron microscopy consistently showed that gold particles immuno-labeled with anti-ATAD3A antibody (directed against ATAD3A N-terminus) accumulated at the contact sites that connect the outer membrane (OMM) and the IMM in HdhQ111 cells (Fig. 2c). In contrast, in HdhQ7 cells, the gold particle-labeled ATAD3A N-terminus was located to the cytosolic side of the OMM (Fig. 2c). These data indicate an enhanced localization of ATAD3A at contact sites in HD cells.

Structural prediction identified two CC domains within the N terminus of ATAD3A that may participate in higher-order ATAD3A oligomerization (Supplementary Fig. 2A). Under non-reducing conditions, ATAD3A dimerization increased both in HdhQ111 cells (Fig. 2d) and in Neuro2a cells exposed to 3-NP (Supplementary Fig. 2B). Both the addition of the chemical crosslinker BMH (bismaleimidohexane) to total cell lysates (in vitro cross-linking) and the treatment of cells with BMH (in vivo cross-linking) led to increased oligomerization of ATAD3A, the extent of which was consistently greater in HdhQ111 cells than in HdhQ7 cells (Fig. 2d). The increased oligomers of ATAD3A was again observed in mitochondrial fractions of HdhQ111 cells after cross-linking with DTSSP (3,3’-dithiobis(sulfosuccinimidyl propionate)) (Supplementary Fig. 2C) and in total lysates of HEK293 cells after crosslinking with DSP (dithiobis(succinimidyl propionate)) (Supplementary Fig. 2D). Notably, ATAD3A oligomers increased in HD YAC128 and R6/2 mouse striata (Fig. 2f), HD patient fibroblasts (Fig. 2g), and HD patient postmortem cortex and caudate nucleus (Fig. 2h), under non-reducing conditions. Our results demonstrate an increase of ATAD3A oligomerization in HD, suggestive of ATAD3A aberrant activation.

After transfecting the truncated mutants of ATAD3A in HEK293 cells, we found that the ΔN50 mutant showed greatly elevated ATAD3A dimers under the non-reducing conditions, whereas both ΔCC and ΔN245 mutants lacking the CC domain abolished the dimerization (Supplementary Fig. 2E). In cells treated with 1% formaldehyde (FA) for crosslinking, ATAD3A ΔN50 was readily oligomerized, and the ΔCC and ΔN245 mutants lost this capacity (Supplementary Fig. 2F). Myc-tagged ATAD3A can form heterodimers with flag-tagged ATAD3A, and deletion of the CC domain (ΔCC and ΔN245) completely blocked the heterodimer (Supplementary Fig. 2G). Thus, the CC domain of ATAD3A is required for ATAD3A oligomerization.

The immuno-density of ATAD3A greatly increased in HD YAC128 and R6/2 mouse cortex and HD patient caudate nucleus (Supplementary Fig. 2H). The protein levels of ATAD3A were unaltered in both mitochondrial fractions and total protein extracts of HD mouse striatum (Supplementary Fig. 2I), excluding the possibility that enhanced ATAD3A dimerization is due to increased protein expression. This increased immunodensity of ATAD3A may be the result of enhanced ATAD3A oligomerization.

### ATAD3A CC domain is required for Drp1 fission activity

**Fig. 3:**
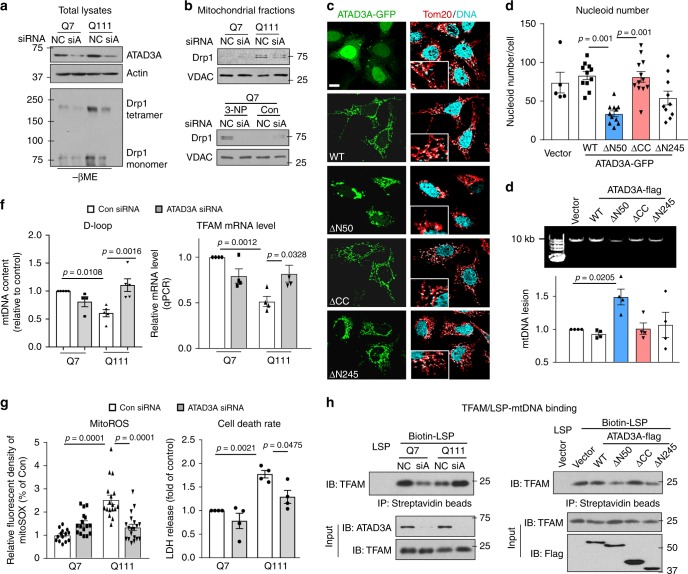
ATAD3A oligomerization impairs mitochondrial fission and mtDNA stability. HdhQ7 and Q111 cells were transfected with control siRNA (NC) or ATAD3A siRNA (siA) for 3 days. **a** Downregulation of ATAD3A was validated by WB. Actin: a loading control. Drp1 polymerization was analyzed by WB with anti-Drp1 antibodies in the absence of β-ME. **b** Mitochondria were isolated from HD striatal cells (Upper) and cells exposed to 5 mM 3-NP for 4 h (Lower). Drp1 mitochondrial level was analyzed by WB. Mitochondrial loading control: VDAC. **c** HeLa cells were transfected with ATAD3A-GFP truncated mutants, shown in Supplementary Fig. 3E, for 48 h. Cells were stained with anti-Tom20 (red) and anti-DNA (cyan) antibodies. The co-localization of DNA and Tom20 was analyzed by confocal microscopy. Insert: the enlarged images. Scale bar: 10 µm. **d** The number of nucleoids immunopositive for both anti-DNA and anti-Tom20 was quantitated by NIH Image J software. 40–50 cells per group were analyzed. Three independent experiments, one-way ANOVA with Tukey’s post-hoc test. **e** Neuro2A cells were transfected with ATAD3A-Flag WT or mutants for 48 h, and total DNA was extracted for qPCR analysis to monitor the mtDNA lesion. Upper: Representative DNA agarose gel of the amplification of the 10 kb mtDNA fragment. Lower: The quantification of mtDNA lesion. Four independent experiments, one-way ANOVA with Tukey’s post-hoc test. **f** mtDNA content was analyzed by qPCR using primers from D-loop (left). TFAM mRNA level was analyzed by qPCR (right). At least 3 independent experiments, one-way ANOVA with Tukey’s post-hoc test. **g** Left: Cells were stained with mitoSOX fluorescent probe to evaluate mitochondrial superoxide production (mitoROS). Right: Cell death rate was measured by LDH release into cytosol. Four independent experiments, one-way ANOVA with Tukey’s post-hoc test. **h** The binding of TFAM and biotinylated mtDNA LSP probe was determined by biotin-streptavidin pull down in HD striatal cells transfected with ATAD3A siRNA (left) and in Neuo2A cells expressing ATAD3A truncated mutants (right). All shown representative blots are from at least 3 independent experiments. All data are mean ± SEM

Expression of ATAD3A ΔN50 mutant induced Drp1 translocation to the mitochondria and Drp1 polymerization, two indicators of Drp1 fission activity^24^. In contrast, expression of ATAD3A ΔCC mutant abolished these events (Supplementary Fig. 3A, B). Conversely, knockdown of ATAD3A by RNA interference (siRNA) in HdhQ111 cells or 3-NP-treated wildtype cells reduced Drp1 polymerization (Fig. 3a) and Drp1 translocation to the mitochondria (Fig. 3b). ATAD3A silencing had no observable effect on other components of mitochondrial dynamics, including Mff, OPA1 and Mfn1 (Supplementary Fig. 3C).

A mutation occurring in the ATPase domain of ATAD3A (ATAD3A K358E) has been shown to induce Drp1-mediated mitochondrial fragmentation^10,25^. However, expression of the K358E mutant in ATAD3A ΔN50 (ΔN50 K358E) had no additional effect on Drp1 translocation to the mitochondria when compared to that in cells expressing ATAD3A ΔN50 (Supplementary Fig. 3D), suggesting that the CC domain is required to recruit Drp1 to the mitochondria. Consistent with a previous study^10^, HeLa cells expressing ATAD3A ΔN50 showed mitochondrial fragmentation, whereas both ATAD3A ΔCC-expressing and ATAD3A ΔN245-expressing cells exhibited elongated and interconnected mitochondria (Supplementary Fig. 3E, F). Neither overexpression of Drp1 nor knockout (KO) of Drp1 nor fission adaptors (Mff or Fis1) influences ATAD3A protein level (Supplementary Fig. 3G, H). In fission adaptors Mff-KO, Mid49-KO, or Fis1-KO MEFs that exhibit elongated mitochondrial network [^26,27^ and Supplementary Fig. 4B], downregulating ATAD3A elicited mitochondrial fragmentation; the number of cells with fragmented mitochondria were significantly higher than those with control shRNA (Supplementary Fig. 4).

### ATAD3A oligomerization impairs mtDNA maintenance in HD

ATAD3A tightly maintains mtDNA nucleoids^13,17^. Expression of ATAD3A ΔN50 mutant decreased the number of nucleoids immuno-positive for both anti-DNA and anti-Tom20 antibodies. In contrast, cells expressing ΔCC mutant exhibited a similar number of nucleoids as those in cells expressing control vector or ATAD3A WT (Fig. 3c, d). Expression of ΔN50 ATAD3A induced more than 50% lesion to mtDNA, which was not seen in cells expressing ΔCC mutant (Fig. 3e). HdhQ111 cells exhibited a decreased copy number of mtDNA and non-coding region of mitochondrial genome D-loop (Fig. 3f, Supplementary Fig. 5A, B), which were corrected by ATAD3A silencing (Fig. 3a, f, Supplementary Fig. 5C). Down-regulation of ATAD3A was sufficient to diminish mitochondrial superoxide production and cell death in HdhQ111 cells (Fig. 3g). Consistent with previous studies^4,5^, we observed decreased mtDNA abundance and nucleoid disorganization in Drp1 KO MEFs (Supplementary Fig. 5D, E). The decrease of mtDNA content due to ATAD3A reduction was abolished in Drp1 KO MEF (Supplementary Fig. 5F). In contrast, knockdown of ATAD3A reduced the number of mitochondrial nucleoids in fission adaptors Mff-KO, Mid49-KO, or Fis1-KO MEFs (Supplementary Fig. 5G). Thus, among fission-related proteins, ATAD3A and Drp1 may form a complex to uniquely maintain mtDNA stability.

mtDNA maintenance, replication, and transcription are controlled by mitochondrial nucleoid, which is composed of a set of mtDNA-binding proteins, including TFAM (the transcription factor A, mitochondrial), SSBP1 (the mitochondrial single-strand binding protein), Twinkle (the mtDNA helicase), POLG and POLRMT (the mtDNA polymerases)^28^. By qPCR, we showed a significant decrease of TFAM mRNA in HdhQ111 cells, which was corrected by ATAD3A knockdown (Fig. 3f and Supplementary Fig. 5H). ATAD3A silencing had no effect on POLG, POLRMT, Twinkle, or SSBP1 (Supplementary Fig. 5H). TFAM specifically binds the light strand promoter (LSP) within the D-loop region of mtDNA, the interaction of which is necessary to activate bidirectional mtDNA transcription^29^. Expression of ATAD3A ΔN50 mutant decreased the binding of TFAM and biotinylated mtDNA LSP probe compared to that in cells expressing ATAD3A WT or ΔCC mutant (Fig. 3h-right), suggesting that ATAD3A oligomerization impairs TFAM-mediated mtDNA packaging. In HdhQ111 cells, the binding of endogenous TFAM to mtDNA LSP was greatly decreased, which was reversed by knockdown of ATAD3A (Fig. 3h-left). Thus, enhancing dimerization of ATAD3A in HD not only resulted in Drp1 activation but also led to mtDNA instability and damage by disrupting TFAM/mtDNA binding. Downregulation of ATAD3A in HdhQ7 cells reduced the binding between TFAM and LSP and slightly decreased mtDNA content and TFAM gene expression when compared to that in HdhQ7 cells expressing control siRNA (Fig. 3f), which is consistent with an important role of ATAD3A steady state in mtDNA nucleoid maintenance^17,28,30^.

### ATAD3A deacetylation promotes its dimerization

**Fig. 4:**
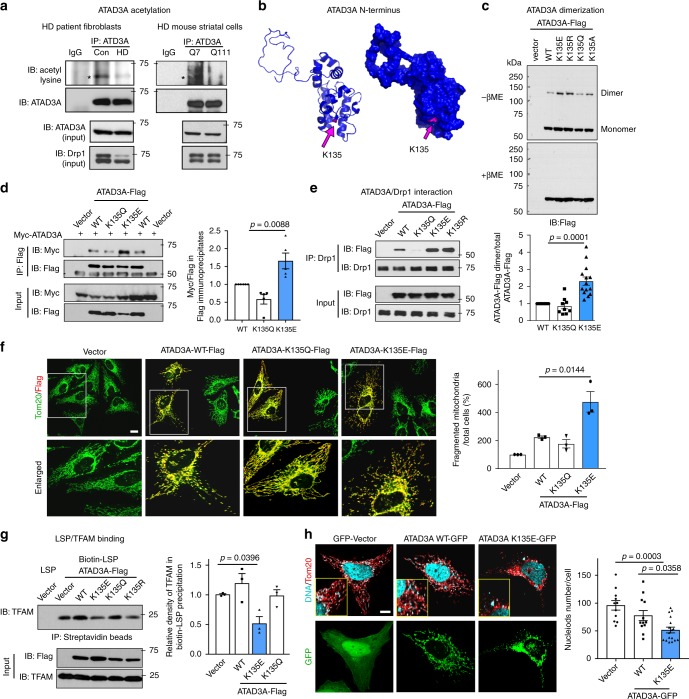
ATAD3A deacetylation. **a** Total cell lysates of HdhQ7 and HdhQ111 cells or fibroblasts of HD patient and control subjects were subject to IP with anti-ATAD3A antibodies followed by WB with anti-acetyl lysine antibodies. Asterisks (*) indicates the acetylated ATAD3A. **b** ATAD3A K135 in peptide 130-AQYADLLAR-138 in human (K134 in mouse) was identified as an acetylated site (also see Supplementary Fig. 6A). The K135 labeled in pink is present on the surface of ATAD3A N-terminus simulated structure. HEK293 cells were transfected with the indicated Flag-tagged K135 mutants or ATAD3A WT for 48 h. **c** ATAD3A dimers were analyzed by WB in the presence or absence of β-ME. Histogram: the quantification of relative density of ATAD3A dimer in the absence of β-ME versus total protein level under reduced conditions. At least 6 independent experiments, one-way ANOVA with Tukey’s post-hoc test. **d** Immunoprecipiates with anti-Flag antibodies were analyzed with anti-Myc antibodies. Histogram: the quantification of relative density of Myc-ATAD3A/ATAD3A-Flag in the immunoprecipitates. Five independent experiments, one-way ANOVA with Tukey’s post-hoc test. **e** IP of total protein lysates was performed with the indicated antibodies. **f** HeLa cells were transfected with Flag-ATAD3A WT or K mutants for 48 h. Cells were stained with anti-Tom20 (green) and anti-Flag (red) antibodies. Mitochondrial morphology was imaged. Scale bar: 10 µm. Histogram: the percentage of cells with fragmented mitochondria to total number of cells. Three independent experiments, one-way ANOVA with Tukey’s post-hoc test. **g** The binding of TFAM and biotinylated mtDNA LSP probe was determined by biotin-streptavidin pull down. Histogram: the relative density of TFAM in the Biotin-LSP precipitates. Three independent experiments, one-way ANOVA with Tukey’s post-hoc test. **h** HeLa cells were stained with anti-Tom20 (red) and anti-DNA (blue) antibodies at the indicated groups. The co-localization of DNA and Tom20 was analyzed by confocal microscopy. Insert: the enlarged images. Scale bar: 10 µm. The number of nucleoids immunopositive for both anti-DNA and anti-Tom20 was quantitated by NIH Image J software. Three independent experiments, one-way ANOVA with Tukey’s post-hoc test. All shown blots are from at least 3 independent experiments. Data are mean ± SEM

To examine the mechanism by which ATAD3A forms dimers in HD, we analyzed ATAD3A acetylation, a post-translational modification that regulates oligomerization of a variety of proteins. ATAD3A was basally acetylated in wildtype HdhQ7 cells and control human fibroblasts, whereas the level of ATAD3A acetylation was greatly reduced in both HdhQ111 cells and HD patient fibroblasts (Fig. 4a), suggestive of a deacetylation. To identify acetylated lysines on ATAD3A, we extracted total cell lysates of HdhQ7 and HdhQ111 cells and immunoprecipitated with anti-ATAD3A antibodies followed by LC-MS/MS analysis. When we focused on the N-terminus of ATAD3A, which mediates dimerization, the interaction with Drp1, and the stability of mitochondrial nucleoids (Fig. 3, Supplementary Figs. 1–3), we identified K134 on the peptide 129-AQYQDKLAR-137 in mice (K135 in human) to be acetylated (Supplementary Fig. 6A) in HdhQ7 cells. The lysine 134 residue, conserved among species, resides in the linker between CC1 and CC2 domain and is present on the surface of ATAD3A simulated structure (Supplementary Fig. 6B and Fig. 4b), which renders it accessible for reversible acetylation. No phosphorylated site within N-terminus of ATAD3A was identified by the mass spectrometry we used in both HdhQ7 and HdhQ111 cells. Although computation analysis predicts serine 48 as a putative phosphorylated site (Supplementary Fig. 6C), mutation (replace serine to either alanine or aspartate) in two conserved serine sites (serine 2 and 48) of ATAD3A had no effects on ATAD3A dimerization (Supplementary Fig. 6D). These data thus led us to determine acetylation as a functionally posttranslational modification of ATAD3A.

To assess whether K135 is important for regulation of ATAD3A dimerization, we replaced K135 with glutamine (K135Q) or Alanine (K135A), which neutralizes the positive charge and mimics the acetylated state. We also used mutant K135E (replaced lysine with glutamic acid) or K135R (replaced lysine with arginine) to mimic deacetylated ATAD3A. The K135E and K135Q mutants were mainly used in our studies. We transfected ATAD3A WT or K mutants into HEK293 cells. Expression of acetyl-deficient K135E or K135R mutant enhanced ATAD3A ability to dimerize; the level of dimer was significantly higher than that in ATAD3A-WT expressing cells (Fig. 4c). While the levels of ATAD3A dimer were comparable between ATAD3A-WT-expressing and ATAD3A K135Q mutant-expressing cells (Fig. 4c), the K135Q mutant was restricted in its ability to form heterodimer with ATAD3A-WT. In contrast, K135E mutant more efficiently bound to ATAD3A WT to form dimer (Fig. 4d). Thus, dimerization of ATAD3A is promoted by deacetylation of ATAD3A at K135 site.

We found that the acetyl-deficient mutant K135E or K135R bound to Drp1 to a greater extent than ATAD3A WT, whereas the acetyl-mimetic ATAD3A K135Q mutant lost the capacity to bind to Drp1 (Fig. 4e, Supplementary Fig. 6E). HeLa cells expressing ATAD3A K135E mutant exhibited extensive mitochondrial fragmentation relative to that in ATAD3A WT-expressing cells (Fig. 4f). Moreover, expression of K135E or K135R mutant reduced the binding of mtDNA LSP to TFAM (Fig. 4g) and decreased the number of mitochondrial nucleoids (Fig. 4h), suggesting mtDNA lesion.

### Peptide DA1 blocks ATAD3A/Drp1 binding in HD

**Fig. 5:**
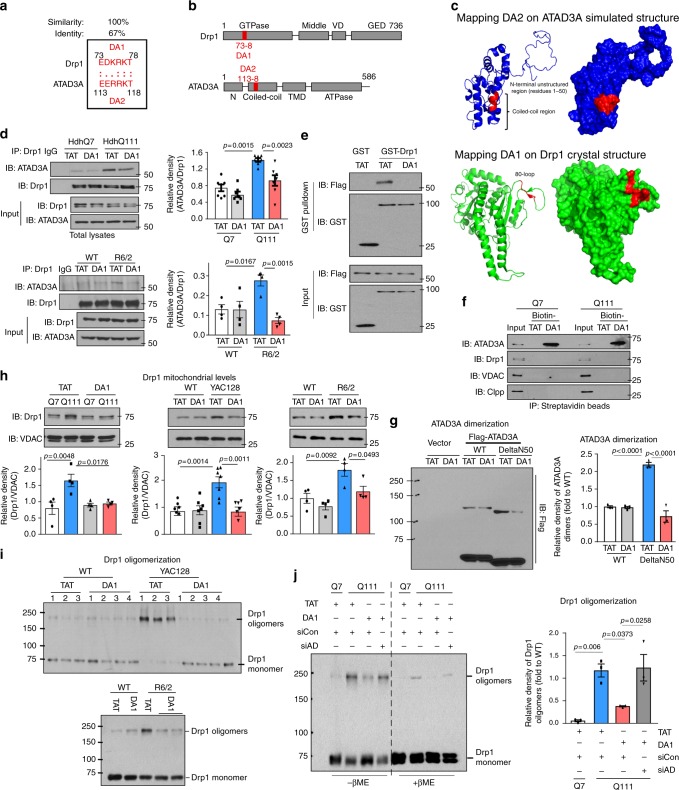
DA1 peptide blocks Drp1/ATAD3A binding. **a** Sequence of homology between Drp1 (human, NP_036192) and ATAD3A (human, NP_001164006). Columns (:) indicate identical amino acids; single dot (·) indicate high similarity between amino acids. **b** Stick drawings of ATAD3A and Drp1 main domains. Highlighted in red are the two regions of homology between the two proteins, region DA1 in Drp1 and the corresponding region DA2 in ATAD3A. **c** Mapping DA1 on crystal structure of Drp1 GTPase-GED fusion construct and DA2 on the N-terminus (residues 1–210) of ATAD3A simulated structure. **d** Upper: HdhQ7 and HdhQ111 cells were treated with TAT or peptide DA1 (1 µM/day for 4 days). Lower: R6/2 or wildtype mice were treated with TAT or DA1 (1 mg/kg/day) from 6 to 12 weeks. The total lysates of cells or mouse striatum were subject to IP analysis. Three independent experiments. **e** DA1 or TAT was incubated with recombinant proteins in vitro for 30 mins. GST pull down followed by WB analysis was carried out. 3 independent experiments. **f** Biotin-conjugated DA1 or TAT (10 µM, each) was incubated with total lysates of HdhQ7 and HdhQ111 cells. Immunoprecipitates were analyzed by WB. 3 independent experiments. **g** HEK293 cells were transfected with ATAD3A-Flag wildtype (WT) or ΔN50 mutant plasmids followed by TAT or DA1 treatment (1 µM, each) for 48 h. ATAD3A oligomerization was analyzed by WB. Three independent experiments. **h** Left: HD cells were treated with TAT or DA1 (1 μM/day for 4 days), 4 independent experiments. YAC128 or wildtype mice from 6 months of age (Middle, 6–7 mice/group), and R6/2 or wildtype mice from 12 weeks of age (Right, 4 mice/group) received either TAT or DA1 (1 mg/kg/day). Drp1 mitochondrial levels were determined by WB. **i** Drp1 oligomerization was analyzed using total striatal lysates of mice (Upper: YAC128-12 months old; Lower: R6/2 mice-12 weeks old). *n* = 6 mice/group. **j** Drp1 oligomerization was analyzed by WB at the indicated groups. Three independent experiments. All data are mean ± SME. One-way ANOVA with Tukey’s post-hoc test

Next, we assessed the functional significance of enhanced Drp1/ATAD3A binding in HD pathology. We previously demonstrated that short peptides interfering with specific protein–protein interactions can serve as pharmacological inhibitors to identify the role of interacting proteins in the pathogenesis of human diseases. In a similar approach to the peptide designs for Drp1 peptide P110^7^ or VCP peptide HV-3^31^, we used L-ALIGN sequence alignment software^32^ and identified one region of homology between Drp1 (human, NP_036192) and ATAD3A (human, NP_001164006) (Fig. 5a). The two regions were marked as region DA1 and DA2. Consistent with our domain mapping of Drp1-ATAD3A interaction (Supplementary Fig. 1G), the regions of homology are only located in the CC domain of ATAD3A and GTPase domain of Drp1 (Fig. 5b). The two regions, present on the surface of Drp1 and ATAD3A (Fig. 5c), are likely available for protein–protein interactions.

We synthesized peptides corresponding to the homologous regions between Drp1 and ATAD3A (Fig. 5a), and conjugated them to the cell permeating TAT protein-derived peptide, TAT_47–57_, in order to enable in vivo delivery^7,31^. These peptides are referred to as DA1 and DA2. DA1 treatment significantly reduced the binding of Drp1/ATAD3A in HdhQ111 cells (Fig. 5d and Supplementary Fig. 7A), in striatal cells exposed to 3-NP (Supplementary Fig. 7B) and in striatal extracts of HD R6/2 mice (Fig. 5d), whereas DA2 showed a trend toward inhibition of the interaction (Supplementary Fig. 7A). Notably, the presence of DA1 abolished the binding of GST-Drp1 and ATAD3A-Flag in vitro (Fig. 5e), indicating a direct inhibition. Peptide DA1 is derived from the loop region of Drp1 GTPase domain, which is critical for protein–protein interaction^33^, and corresponds to a sequence in the first CC domain of ATAD3A (Fig. 5b). The sequence of DA1 in Drp1 is highly conserved among species (Supplementary Fig. 7C). Treatment with DA1 did not influence the interactions of Drp1 with its mitochondrial adaptors Mff, Fis1 and MIEF1 in HdhQ111 cells (Supplementary Fig. 7D), suggesting a selectivity.

To examine the specificity of DA1, we incubated biotin-conjugated DA1 or TAT with total protein lysates of HD striatal cells followed by IP with streptavidin beads. Biotin-DA1 pulled down endogenous ATAD3A and not Drp1 (Fig. 5f). No detectable binding was observed between biotin-DA1 and the mitochondrial matrix protein ClpP or the OMM protein VDAC (Fig. 5f). Moreover, biotin-DA1 bound to ATAD3A-Flag and not to GST-Drp1 recombinant protein in vitro (Supplementary Fig. 7E). DA1 had no effects on Drp1 oligomerization-induced GTPase activity in vitro, excluding a direct role on Drp1 (Supplementary Fig. 7F). These results suggest that DA1 competes with Drp1 to bind to ATAD3A, which in turn interferes with the interaction of Drp1/ATAD3A. Furthermore, DA1 treatment abolished the dimerization of ATAD3A in cells exposed to 3-NP (Supplementary Fig. 7G), and in cells expressing ATAD3A ΔN50 mutant (Fig. 5g) or ATAD3A acetyl-deficient mutant K135E (Supplementary Fig. 7H), whereas it had minor effect on ATAD3A dimerization in normal cells or in cells expressing ATAD3A wildtype. Thus, DA1 preferentially prevents ATAD3A oligomerization induced under stress or diseased conditions, rather than that in control cells. This is likely due to the lower level of ATAD3A oligomers under normal conditions or the steady state of the wildtype protein.

DA1 treatment abrogated Drp1 translocation to the mitochondria and Drp1 polymerization in HD mouse striatal cells and striatum of HD YAC128 and R6/2 mice (Fig. 5h, i), but it did not affect other mitochondrial fusion and fission-related proteins (Supplementary Fig. 7I). The inhibitory effect of DA1 on Drp1 tetramer formation was abolished upon ATAD3A knockdown (Fig. 5j), indicating that DA1 requires the presence of ATAD3A to suppress Drp1 activation. Together, our results support the hypothesis that DA1 peptide blocks ATAD3A and Drp1 interaction in HD by directly binding to ATAD3A, which in turn suppresses ATAD3A dimerization and inhibits the activation of both Drp1 and ATAD3A.

### DA1 improves mitochondrial function in HD cells

**Fig. 6:**
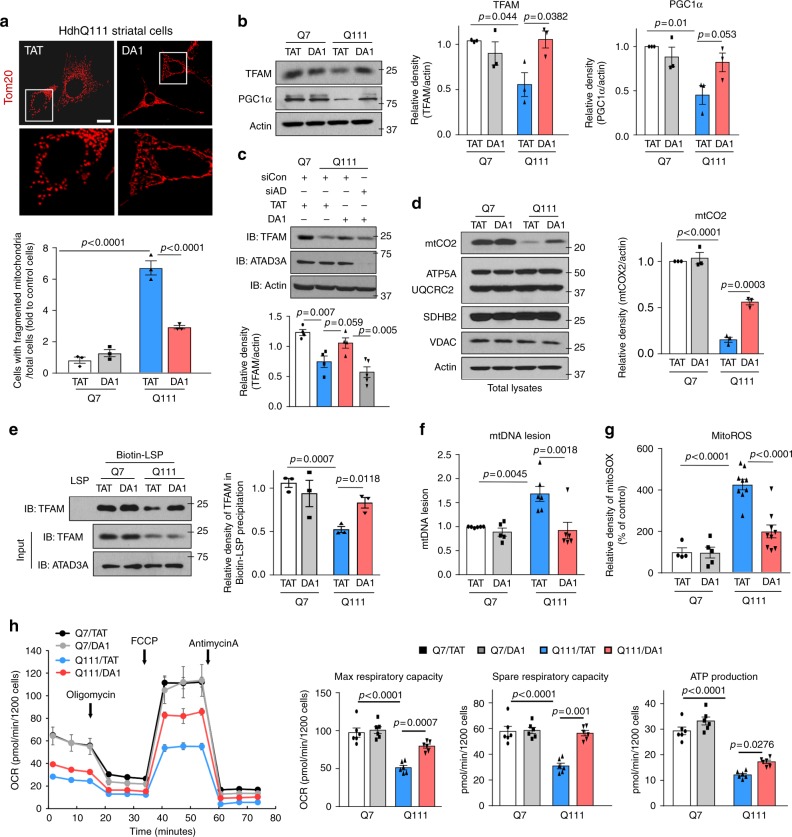
DA1 treatment reduces mitochondrial damage and cell death in HD cell cultures. HdhQ7 and HdhQ111 striatal cells were treated with control TAT or peptide DA1 (1 µM/day for 4 days). **a** Mitochondrial morphology was determined by staining cells with anti-Tom20 antibody. Scale bar: 10 µm. The percentage of cells with fragmented mitochondria relative to the total number of cells was quantitated. Three independent studies, one-way ANOVA with Tukey’s post-hoc test. **b** TFAM and PGC1α protein levels were analyzed by WB. Histogram: the relative density of TFAM and PGC1α to Actin. Three independent studies, one-way ANOVA with Tukey’s post-hoc test. **c** HdhQ7 and HdhQ111 cells were transfected with control siRNA (siCon) or ATAD3A siRNA (siAD) followed by treatment with TAT or DA1 (1 µM/day for 4 days). TFAM and ATAD3A protein levels were analyzed by WB. Histogram: the relative density of TFAM to Actin. Four independent studies, one-way ANOVA with Tukey’s post-hoc test. **d** mtDNA-encoded or nuclear-encoded mitochondrial electron transport proteins were analyzed by WB with the indicated antibodies. Actin: a loading control. VDAC: a mitochondrial loading control. Histogram: the quantitation of relative mtCO2 protein level to Actin. Three independent studies, one-way ANOVA with Tukey’s post-hoc test. **e** TFAM and mtDNA LSP interaction was analyzed by biotin-streptavidin pull down. Histogram: the relative density of TFAM in the Biotin-LSP precipitates. Three independent experiments, one-way ANOVA with Tukey’s post-hoc test. **f** mtDNA lesion was measured by qPCR. Representative DNA gel is showed in Supplementary Fig. 7J. Five independent studies, one-way ANOVA with Tukey’s post-hoc test. **g** mitoROS was evaluated by mitoSOX fluorescent probe. At least 100 cells per group were analyzed. At least 3 independent studies, one-way ANOVA with Tukey’s post-hoc test. **h** Mitochondrial respiratory activity was determined by a seahorse analyzer. Three independent studies, one-way ANOVA with Tukey’s post-hoc test. All data are mean ± SEM

DA1 treatment reduced the number of cells with fragmented mitochondria in HdhQ111 cells relative to the cells treated with control peptide TAT (Fig. 6a). The levels of TFAM and PGC1α, reflecting mitochondrial bioenergetics activity^34^, were decreased in HdhQ111 cells and were restored by DA1 treatment (Fig. 6b). The recovery of DA1 on mitochondrial biogenesis in HD mutant cells requires the presence of ATAD3A, as demonstrated by the finding that ATAD3A knockdown abolished the elevation of TFAM level by DA1 treatment (Fig. 6c). DA1 treatment corrected the decreased mtCO2 protein level in HdhQ111 cells, whereas it had no effect on nuclear-encoded mitochondrial respiratory chain components (Fig. 6d). Notably, treatment with DA1 restored the binding of TFAM and mtDNA (Fig. 6e), increased mtDNA copy number, diminished mtDNA lesion, and suppressed mitochondrial oxidative stress in HdhQ111 cells relative to cells treated with control peptide TAT (Fig. 6f, g and Supplementary Fig. 7J). Furthermore, DA1 treatment attenuated mitochondrial respiratory defects in HdhQ111 cells; it improved maximal and spare respiratory capacity and ATP production, with minor effects on HdhQ7 cells (Fig. 6h).

**Fig. 7:**
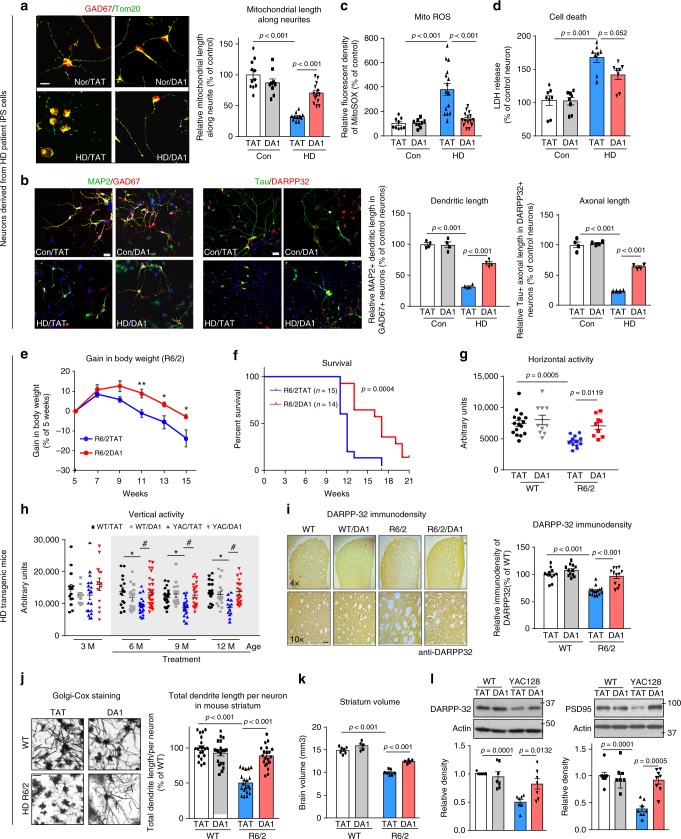
DA1 treatment reduces HD-associated neuropathology. **a** Neurons were stained with anti-GAD67 antibodies to indicate striatal neurons and anti-Tom20 antibodies to label mitochondria. See Supplementary Fig. 7K for a cluster of neurons. Scale bar: 10 μm. Mitochondrial length along the neurite of GAD67-immunopositive neurons was quantitated. At least 50 neurons/group were analyzed. **b** Neurons were stained with anti-MAP2/anti-GAD67 (left) or anti-Tau/anti-DARPP32 (right) to indicate dendritic and axonal morphology, respectively. MAP2^+^ dendrite and Tau^+^ axon length in neurons were quantitated. At least 50 neurons/group were analyzed. Scale bar: 10 μm. **c** mitoROS was determined by mitoSOX probe. **d** Cell death induced by BDNF withdrawal for 24 h was determined by the release of LDH. Three independent experiments. HD R6/2 and wildtype mice were treated with either TAT or DA1 from 6 weeks (see Supplementary Fig. 8A). **e** Body weight was recorded. **p < 0.001, *p < 0.05 vs. HD mice treated with TAT. Student *t*-test. TAT: 21 mice; DA1: 15 mice. **f** Survival of R6/2 mice from the age of 6 to 21 weeks was analyzed by Log-rank (Mantel-Cox) test. **g** locomotion activity of R6/2 mice (12 weeks old) was determined (WT/TAT: 16 mice; WT/DA1: 10 mice; R6/2/TAT: 13 mice; R6/2/DA1: 9 mice). Shown is horizontal activity. **h** YAC128 and wildtype mice were treated with TAT or DA1 from the age of 3 to 12 months (see Supplementary Fig. 8A). Locomotion activity was monitored (*n* = 12–20 mice/group). Shown is vertical activity. Two-way ANOVA with Tukey’s post-hoc test. #*p* < 0.05 vs. HD mice treated with TAT; *p < 0.05 vs. wildtype mice treated with TAT. **i** DARPP-32 immunodensity was assessed in the dorsolateral striatum of TAT-treated or DA1-treated R6/2 mice (12 weeks old). Scale bar: 100 µm. *n* = 6 mice/group. **j** Golgi-Cox staining of mouse brain was shown. The total dendritic length/neuron was quantitated. 20 neurons/group was analyzed. Scale bar: 10 µm. **k** Striatum volume was assessed. *n* = 5–7 mice/group. **l** DARPP-32 and PSD95 protein levels in YAC128 mouse (12 months old) striatal extracts were analyzed by WB. *n* = 7 mice/group. All data are mean ± SEM. One-way ANOVA with Tukey’s post-hoc test in **a**–**d**, **g**, **i**–**l**

In neurons derived from HD patient-iPS cells, consistent with our previous study^7^, mitochondria were extensively fragmented along the neurites of neurons immune-positive for anti-GAD67 (a marker of GABAergic striatal neurons). In contrast, DA1 treatment increased the length of mitochondria along the neurites of GAD67^+^-neurons (Fig. 7a and Supplementary Fig. 7K). Moreover, DA1 treatment improved MAP2^+^ dendritic and Tau^+^ axonal outgrowth of striatal neurons derived from HD patient iPS cells (Fig. 7b). The improvement on mitochondrial and neuronal morphology by DA1 treatment was accompanied with decreased mitoROS and cell death in neurons derived from HD patients (Fig. 7c, d). DA1 treatment had no observed effects on mitochondrial morphology and neuronal survival in cells derived from iPS cells of normal subject (Fig. 7a–d). Thus, blocking Drp1/ATAD3A interaction by DA1 peptide inhibitor improves mitochondrial bioenergetics activity and reduces mitochondrial impairment in HD cells.

### DA1 reduces behavioral deficits in HD mice

To test whether DA1 treatment improves the disease phenotypes in HD transgenic mice, we subcutaneously treated HD R6/2 from the age of 6 to 21 weeks, and YAC128 mice from the age of 3 to 12 months, with either control peptide TAT or peptide DA1 (1 mg/kg/day) (Supplementary Fig. 8A). DA1, administered subcutaneously at 1 mg/kg/day, entered the brain (Supplementary Fig. 8B), and did not cause weight loss or visible side effects in organs (Supplementary Fig. 8C–D). In HD R6/2 mice, while sustained DA1 treatment moderately suppressed body weight loss (Fig. 7e), the treatment greatly prolonged the survival of mice (Fig. 7f). The treatment had no effect on life-span of wildtype mice (Supplementary Fig. 8D). DA1 treatment also increased R6/2 mice horizontal activity and total traveled distance at the age of 12 weeks (Fig. 7g and Supplementary Fig. 8E), and reduced the severity of clasping behavior over a four-week observation period (Supplementary Fig. 8F). In YAC128 mice that exhibit progressive deficits in movement activity, sustained treatment with DA1 improved general movement activity starting at the age of 6 months, and the protective effect lasted until the age of 12 months (Fig. 7h and Supplementary Fig. 8G). In contrast, sustained treatment with DA1 had minor effects on behavioral status of wildtype mice through our studies.

### DA1 reduces HD neuropathology in mice

DARPP-32 is a marker of medium spiny neurons (MSN), the decreased level of which is used as a measure of striatal neuronal survival in HD^35^. In R6/2 mice, we observed a decrease in the area occupied by DARPP-32-immunostained cells in the striatum, which was increased by DA1 (Fig. 7i). Treatment with DA1 also improved dendritic morphology of MSN in R6/2 mice, as assessed by Golgi-Cox staining (Fig. 7j), and increased striatum volume of R6/2 mice (Fig. 7k). In YAC128 mice, DA1 treatment increased the protein levels of DARPP-32 and PSD95 (a post-synaptic marker that maintains the postsynaptic density^36^) (Fig. 7l). These results suggest that DA1 treatment improves striatal neuronal survival in vivo.

**Fig. 8:**
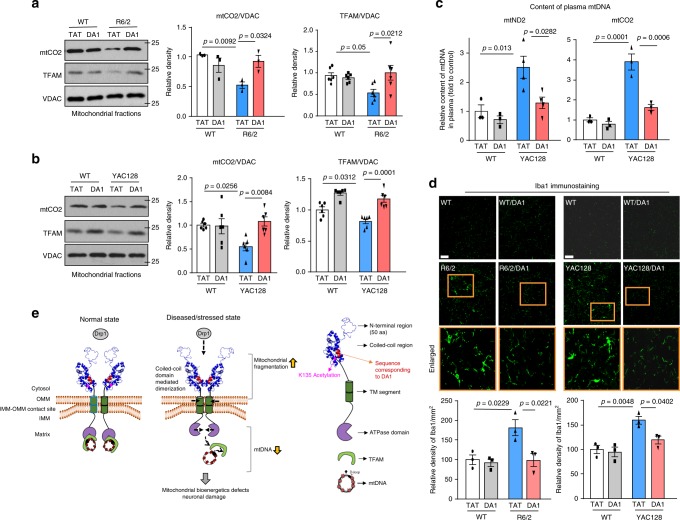
DA1 treatment reduces mtDNA damage in HD mice. Mitochondria were isolated from striatum of R6/2 or age-matched wildtype mice (12 weeks old) (**a**), and YAC128 or age-matched wildtype mice (6 months old) (**b**). Protein levels of mtCO2 and TFAM were determined by WB. VDAC: a mitochondrial loading control. Histogram: the relative density of mtCO2 and TFAM to VDAC. *n* = 3–6 mice/group. One-way ANOVA with Tukey’s post-hoc test. **c** Plasma samples were harvested from YAC128 mice or wildtype mice (6 months old). The mtDNA content in plasma was examined by qPCR. One-way ANOVA with Tukey’s post-hoc test. *n* = 6 mice/group. **d** Brain coronal sections (10 µm) of R6/2 (left, 12 weeks old) or YAC128 (right, 6 months old) mice were stained with anti-Iba1 antibody to determine microglial activity. Boxed images were enlarged on the bottom. Scale bar: 10 μm. Histogram: the quantitation of Iba1 immunodensity/mm^2^. *n* = 3 mice/group. ANOVA with Tukey’s post-hoc test. All data are mean ± SEM. **e** A summary scheme. Under stressed conditions, such as HD, ATAD3A forms oligomers due to K135 deacetylation via its coiled-coil domain, and recruits fission protein Drp1 to the mitochondria where ATAD3A and Drp1 form a complex. This leads to mitochondrial fragmentation. ATAD3A oligomerization impairs mtDNA maintenance by disrupting the binding between TFAM and mtDNA, resulting in the loss of mtDNA and subsequent mitochondrial bioenergetics defects. As a result, ATAD3A oligomerization simultaneously causes mitochondrial fragmentation and mitochondrial bioenergetics defects, which lead to mitochondrial dysfunction and neuronal cell death. DA1 peptide directly binds to ATAD3A to block ATAD3A and Drp1 interaction. Treatment with DA1 quenches ATAD3A oligomerization which therefore reduces excessive mitochondrial fission and the loss of mtDNA. Consequently, treatment with DA1 both in HD cultures and in HD animals reduces HD-associated neuropathology

The protein levels of TFAM and mtDNA-coded protein mtCO2 were reduced in both R6/2 and YAC128 mice, which was corrected by DA1 treatment (Fig. 8a, b). Again, DA1 treatment had no observable effects on nuclear-encoded mitochondrial proteins (Supplementary Fig. 8H). Treatment with DA1 increased PGC1α protein levels in YAC128 mouse striatum until 12 months of age (Supplementary Fig. 8i). These results are consistent with our findings in culture and suggest that DA1 treatment improves mtDNA maintenance and bioenergetics activity in mouse models of HD. MtDNA detected in the plasma of HD mice and HD patients can serve as a biomarker that correlates with disease progression^37,38^. In YAC128 mice, which exhibit chronic HD-like neurodegeneration with a year-long disease development, plasma mtDNA content was increased at the age of 6 months [Fig. 8c and^37^], an age prior to the onset of neurodegeneration. DA1 treatment abolished this increase (Fig. 8c), further indicating a protective effect on mitochondrial function in HD. Elevated plasma levels of mtDNA can act as cell damage-associated molecular pattern (DAMP), eliciting inflammation^39–42^. In HD R6/2 and YAC128 mouse striatum, activated microglial cells marked by anti-Iba1 antibody indicated an induction of inflammation. Treatment with DA1 corrected this inflammatory response (Fig. 8d), which is likely the result of suppression of mtDNA lesion.

## Discussion

In this study, we find that under stressed conditions, such as in HD, ATAD3A, via pathological oligomerization, exhibits a gain-of-function that causes mitochondrial fragmentation and impairs mitochondrial biogenesis, resulting in neurodegeneration (Fig. 8e). This hypothesis is further supported by our results that the reduction of ATAD3A dimers and subsequent ATAD3A/Drp1 binding by treatment with DA1 suppressed mitochondrial fragmentation and mtDNA damage, and reduced HD neuropathology. Therefore, our findings, by providing evidence for a crucial role of ATAD3A in maintaining mitochondrial integrity and neuronal survival, reveal insights into the pathogenesis of HD, and therefore highlight a promising therapeutic strategy for HD.

ATAD3A has been suggested to locate in the contact sites between the IMM and the OMM^10,12^, and to be enriched in mitochondria-associated membranes (MAMs)^11,13,16^. We showed that endogenous ATAD3A was more abundant in the contact sites of mitochondria in HD mutant cells. Thus, it is possible that ATAD3A interacts with Drp1 on the mitochondrial contact sites of HD cells, where Drp1, upon translocation to the mitochondria, can have a great access to ATAD3A. Mitochondrial fission adaptors Mff, Fis1, and MiD49/51 have been shown to recruit Drp1 to the mitochondria via protein–protein interaction^26,43–45^. ATAD3A does not seem to affect Mff, Fis1, and Mid49/51, and vice versa. However, either knockout or overexpression of ATAD3A elicits mitochondrial fragmentation. In the current study, we cannot exclude the possibility that ATAD3A may function through Drp1 adaptors. Though Drp1/ATAD3A direct binding is observed in vitro, to what extent the binding in vivo leads to the observed phenotypes is less apparent. Whether DA1 has additional mechanism of actions that are implicated in the HD pathology remains to be explored.

AAA family proteins often function as hexameric rings via conserved ATPase domain^46^. We found that the CC domain of ATAD3A is in fact required for ATAD3A oligomerization, as deletion of the CC domain abrogated ATADA3A oligomerization even when the ATPase domain is present. Moreover, ATAD3A oligomerization via the CC domain seems to be a key linking between mitochondrial fission and mtDNA stability. This conclusion is supported by our findings that the prevention of ATAD3A oligomerization by DA1 peptide corrected mitochondrial fragmentation and mtDNA depletion in HD models. Genetically defective ATP-binding in the ATPase domain reduces the ATPase activity of ATAD3A but does not affect ATAD3A steady state or mtDNA content^14^. Though expression of the ATPase deficient mutants is associated with mitochondrial fragmentation^10,14,25^, the deletion of the first 50 amino acids in ATAD3A enhances recruitment of Drp1 to the mitochondria. Thus, the CC domain rather than the ATPase enzyme activity of ATAD3A functions as a linker, connecting signals between in and outside mitochondria. The first 50 amino acids are non-structured. Deletion of the 50 amino acids enhanced ATAD3A oligomerization and the binding of ATAD3A to Drp1, and disrupted TFAM/mtDNA complex. Thus, the first 50 amino acids may act as an inhibitory segment to retain the steady state of ATAD3A.

We found that ATAD3A deacetylation at residue K135 is required to its dimerization that is essential for induction of mitochondrial fragmentation and mtDNA lesion. Thus, our findings suggest a model in which in resting cells ATAD3A is acetylated at K135 to maintain its steady state, whereas deacetylation of ATAD3A at this specific site, such as under HD conditions, serves as a switch to promote its oligomerization via the CC domain, which results in a signaling-on conformation of ATAD3A, leading to aberrant activation of the protein. Future structural analysis of such conformational changes by mutating the K135 residue would be informative. It would be also interesting to identify the enzymes that are responsible for ATAD3A deacetylation in the context of HD.

ATAD3A can co-purify with TFAM^17^ but does not directly contact mtDNA^47^. Manipulation of ATAD3A selectively regulates TFAM, which influences the binding between TFAM and mtDNA. Thus, ATAD3A may act upstream of TFAM. Disturbance of ATAD3A steady state by oligomerization may break the molecular balance between ATAD3A and TFAM/mtDNA, which in turn impairs mtDNA stability and abundance. Interestingly, our proteomic analysis identified TFAM as one of interactors of Drp1 in HD. Similar to ATAD3A, TFAM can be detected on the contact sites of mitochondria^13^. Thus, centered on ATAD3A, Drp1, ATAD3A, and TFAM may form a complex on mitochondria where membrane fission links with nucleoid dynamics.

In summary, our findings suggest that ATAD3A is a potential therapeutic target for combating the mitochondrial damage and neurodegeneration underlying HD and other neurological disorders that feature mitochondrial fragmentation and bioenergetic failure. DA1 biological effects require the presence of ATAD3A, and DA1 influences neither the binding of Drp1 with its mitochondrial adaptors nor the levels of mitochondrial fusion/fission proteins. These characteristics of DA1 make it a unique inhibitor to modulate both mitochondrial fission impairment and bioenergetic defects under pathological conditions. Thus, DA1 peptide might be a useful strategy to prevent or slow the progression of neurodegeneration.

## Methods

### Antibodies and reagents

Protein phosphatase inhibitor, protease inhibitor cocktails, Flag peptide, GST control protein, and 3-nitropropionic acid (3-NP, N5636) were purchased from Sigma-Aldrich. DSP (dithiobis[succinimidylpropionate], 22585), DTSSP (3,3´-dithiobis[sulfosuccinimidylpropionate], 21578), and BMH (bismaleimidohexane, 22330) were purchased from Thermo Fisher Scientific. Antibodies used in this study are listed below: antibodies for Drp1 (61113, 1:1000) and OPA1 (612607, 1:1000) were purchased from BD bioscience. Antibodies of ATAD3A (H00055210-D01, 1:1000), Mfn1 (H00055669-M04, 1:1000), Mfn2 (H00009927-M01, 1:1000) were from Abnova. Antibodies of CHCHD3 (25625-1-AP, 1:1000), YME1L1 (11510-1-AP, 1:500), Mff (17090-1-AP, 1:1000), Fis1 (10956-1-AP, 1:1000), MIEF1 (20164-1-AP, 1:1000), and mtCO2 (55070-1-AP, 1:1000) were from ProteinTech; Tom20 (sc-11415, 1:1000), GFP (sc-9996, 1:2000), Myc (sc-40, 1:1000), Ac-lysine (AKL5C1, sc-32268), and GST (sc-138, 1:1000) antibodies were from Santa Cruz Biotechnology. Mitofilin (ab110329, 1:1000), Cytochrome *C* (ab110325, 1:10000), VDAC (ab14734, 1:2000), TFAM (ab131607, 1:2000), ClpP (ab124822, 1:2000), DARPP-32 (ab40801, 1:10000), and mitochondrial OXPHOS cocktail (ab110413, 1:1000) antibodies were from Abcam. FLAG (F3165, 1:2000) and β-Actin (A1978, 1:10000) antibodies were from Sigma. Antibody of PGC1α (3934–100, 1:500) was from Biovision. Anti-DNA (CBL186, 1:500) and anti-GAD67 (MAB5406, 1:500) antibodies were from Millipore. Antibodies of PSD95 (2507, 1:1000), Acetylated-Lysine (9441 S, 1:1000), and MAP2 (4542, 1:300) were from cell signaling. Iba1 (019–19741, 1:500) antibody was from Wako Chemicals. Antibody of Tau (T1308-1, 1:300) was purchased from rpeptide. HRP-conjugated anti-mouse and rabbit secondary antibodies were from Thermo Fisher Scientific. VeriBlot secondary antibody (HRP) (ab131366, 1:2000) that does not recognize heavy or light chains was from Abcam. Alexa 488/568/405 goat anti-mouse and rabbit secondary antibodies were from Life Technologies. Streptavidin beads (20357) were purchased from Thermo Fisher Scientific. Glutathionesepharose beads were obtained from GE Healthcare. GST-Drp1 recombinant protein was from Abnova.

### Cells culture

HEK293T, HeLa, and Neuro2A cells were cultured in DMEM supplemented with 10% (v/v) heat-inactivated FBS and 1% (v/v) antibiotics (100 unit/ml penicillin, 100 ug/ml streptomycin).

Drp1 WT and KO MEFs were obtained from Dr. Hiromi Sesaki (Johns Hopkins University). MFF WT and KO MEFs, as well as Fis1 KO MEFs, were obtained from Dr. David C. Chan (California Institute of Technology). MiD49 KO and WT MEFs were obtained from Mike Ryan (Monash Biomedicine Discovery Institute, Monash University). The cells were maintained in DMEM supplemented with 10% FBS and 1% penicillin/streptomycin.

HD patient fibroblasts (obtained from Coriell Institute, USA) and normal fibroblasts were maintained in MEM supplemented with 15% (v/v) FBS and 1% (v/v) penicillin/streptomycin.

iPS cells from a normal subject and a HD patient (carrying 41 CAG repeats) were differentiated into neurons using the protocol from our previous studies^7,31^. Briefly, the iPS cells were plated onto 6-well plates precoated with 2.5% Matrigel and allowed to reach 90% confluence in feeder-free medium. For the first 10 days, the cells were treated with SB431542 (10 μM; Tocris Bioscience) and Noggin (100 ng/ml) in Neural Media (NM) containing Neurobasal and DMEM (1:1), B-27 supplement minus vitamin A (50×, Invitrogen), N2 supplement (100×, Invitrogen), GlutaMax (Invitrogen, 100×), FGF2 (20 ng/μl) and EGF (20 ng/μl), and 100 units/ml penicillin and 100 μg/ml streptomycin). For the next 10 days, the cells were treated with human recombinant Sonic hedgehog (SHH, 200 ng/ml), DKK1 (100 ng/ml) and BDNF (20 ng/ml), and 10 μM Y27632 (Sigma) in neuronal differentiation medium containing Neurobasal and DMEM (1:3), B27, N2, GlutaMax, and PS. Cells were then switched to treatment with BDNF (20 ng/ml), ascorbic acid (200 µM, Sigma-Aldrich), cAMP (0.5 mM, Sigma-Aldrich), and Y27632 (10 µM) in neuronal differentiation medium. All growth factors were purchased from PeproTech (Rocky Hill, NJ, USA). Twenty days after the initiation of differentiation, the neurons (about 5000 cells) were plated onto 12-mm poly-D-lysine/laminine-coated coverslips and grown in 24-well plates in neuronal differentiation medium.

All of the above cells were maintained at 37 °C in 5% CO2-95% air.

Mouse striatal cell lines HdhQ7 and Q111 cells are immortalized from knock-in mice carrying 7 and 111 CAG repeats, respectively, in the mouse htt gene^48^. HdhQ7 and HdhQ111 purchased from CHDI Foundation were cultured in DMEM supplemented with 10% (v/v) heat-inactivated FBS, 1% (v/v) penicillin/streptomycin, and 400 µg/ml G418 at 33 °C with 5% CO_2_. Cells within 14 passages were used in all experiments.

### Plasmids and transfection

Human ATAD3A was amplified from HEK293T cDNA samples through standard PCR methods, and then cloned into pCMV-Myc, pcDNA3.1 (+)-Flag, pEGFP-N1 vectors. ATAD3A truncated mutants were constructed using overlap cloning techniques. ATAD3A point mutant K358E, and K135 mutants (K135E, K135R, K135Q, and K135A) and S2S48 mutants were constructed by using QuickChange II Site-Directed Mutagenesis Kit (Agilent Technologies). Drp1 truncated mutants were described in our previous study^49^. Cells were transfected with plasmids using *Trans*IT®-2020 transfection reagent (Mirus Bio LLC, Madison, WI) according to manufacturer’s instruction.

### Knockdown of ATAD3A

For silencing ATAD3A, cells were transfected with control siRNA or mouse ATAD3A siRNA using TransIT-TKO Transfection Reagent (Mirus Bio LLC, Madison, WI) following the protocol recommended by the manufacturer. The siRNA oligo sequences are as follows (only the sense strand is shown): control siRNA (non-targeting): UUCUCCGAACGUGUCACGU; ATAD3A: AGAUGGAGCUGAGGCAUAA (Dharmacon, GE). For knockdown of ATAD3A in MEFs, control shRNA and ATAD3A shRNAs (Sigma, SHCLNG-NM_179203) were lentivirally infected in MEFs followed by selection with 1 µg/ml puromycin. The information of shRNAs used here are as follows: shATAD3A#1(TRCN0000242003), shATAD3A#2 (TRCN0000242004) and shATAD3A#3 (TRCN0000241479).

### Drp1 interactome

Forty days after neuronal differentiation, neuron culture derived from HD patient-iPS or normal subject-iPS cells were lysed in total lysis buffer (10 mM HEPES-NaOH, pH 7.9, 150 mM NaCl, 1 mM EGTA, 1% Triton X-100, and protease inhibitor cocktail). Three milligram of the lysates were immunoprecipitated using anti-Drp1 antibody overnight followed by 2 h incubation with protein A/G beads. Bound proteins were eluted by 0.1 M glycine (pH 3.0). The proteins from each sample were then precipitated using 30% trichloroacetic acid (v/v), and the protein pellet was washed using ice-cold acetone. The pellet was air-dried, and the proteins were then trypsinized and analyzed by tandem mass spectrometry.

Digested peptides were reconstituted with 0.1% formic acid and analyzed by LC-MS/MS. Separation of peptides via capillary liquid chromatography was performed using Waters nanoAquity system (Waters Corp., Milford, MA). Mobile phase A (aqueous) contained 0.1% formic acid in 5% acetonitrile, and mobile phase B (organic) contained 0.1% formic acid in 85% acetonitrile. Separation was achieved using a C18 column (75 µm × 20 cm, Waters Corp., Ethylene Bridged Hybrid column BEH300) through a 150 min gradient of 6 to 45% mobile phase B at a flow rate of 0.30 µL/min. Mass spectrometry analysis was performed using a hybrid linear ion trap Orbitrap Velos mass spectrometer (LTQ-Orbitrap Velos, Thermo, Waltham, MA). Survey scan was operated at 60,000 resolution, followed by eight standard collision-induced dissociation (CID) fragmentations in a data-dependent manner. Dynamic exclusion was enabled as the following: repeat count, 2; repeat duration, 30 s; exclusion list size, 250; and exclusion duration, 120 s.

Acquired tandem mass spectra were searched against the Uniprot human protein database (downloaded on May 5, 2012), containing 40,464 protein entries. A decoy database containing the reversed sequences of all the proteins was appended to estimate false discovery rate. Protein identification using Sequest or ProLuCID and DTASelect was performed using the Integrated Proteomics Pipeline (IP2, Integrated Proteomics Applications, Inc. San Diego, CA). The precursor mass accuracy was limited to 15 ppm for spectra acquired; the product ions mass accuracy was set at 0.6 Da. Fully tryptic enzyme specificity and upto two missed cleavages were allowed. Static modifications included carbamidomethylation on cysteines (57 Da), and variable modifications included oxidation on methionines (16 Da). Isotopic C^13^ incorporated ions were automatically included. DTASelect was applied to generate search results of peptide-to-spectra matches (PSMs) with a max false discovery rate (FDR) of 5%, yielding a protein FDR of less than 1% with at least two peptides per protein being assigned.

Cellular location, molecular and cellular function, and enriched mitochondrial candidates of Drp1-interacting proteins were analyzed using Ingenuity IPA software.

### Analysis of ATAD3A oligomers

To analyze dimerization of ATAD3A under non-reducing conditions, the cells or tissues were harvested and lysed with total lysates buffer. An equal amount of proteins was subject to 8% SDS-PAGE analysis in the presence or absence of β-mercaptoethanol (β-ME). For in vitro BMH crosslinking, the cell lysates were incubated with 1 mM BMH at r.t. for upto 25 min and quenched with 0.1% β-ME, followed by SDS-PAGE analysis. For in-vivo crosslinking, the cells were treated with cell-permeable crosslinker BMH (1 mM) for 20 min at 37 °C. After crosslinking, the cells were quenched in PBS with 0.1% β-ME. For DSP crosslinking, the cells were treated with cell-permeable crosslinker DSP for 5 min at 37 °C, followed by quenching and washing with 10 mM Glycine in PBS. For formaldehyde (FA) crosslinking, the cells were incubated with PBS buffer containing 1% FA for 20 min at r.t., and the crosslinking reaction was terminated by washing in PBS containing 100 mM glycine. The cells were then lysed and subject to 8% SDS-PAGE analysis. For DTSSP crosslinking of proteins in mitochondrial fractions, isolated mitochondria samples were suspended in the mitochondrial lysis buffer (250 mM sucrose, 20 mM HEPES-NaOH, pH 7.5, 10 mM KCl, 1.5 mM MgCl_2_, 1 mM EDTA, 1 mM EGTA and protease inhibitor). An equal amount of crude mitochondria was incubated at 30 °C in the presence of 0.5 mM DTSSP. The cross-linking was terminated by the addition of SDS loading buffer. The samples were subject to 8% SDS-PAGE analysis.

### Isolation of mitochondria-enriched fractionations

Cells were washed with cold phosphate-buffered saline (PBS) and incubated on ice in mitochondrial lysis buffer (250 mM sucrose, 20 mM HEPES-NaOH, pH 7.5, 10 mM KCl, 1.5 mM MgCl2, 1 mM EDTA, 1 mM EGTA, protease inhibitor cocktail, and phosphatase inhibitor cocktail) for 30 min. The cells were scraped and then disrupted 15 times by repeated aspiration through a 25-gauge needle. Mouse brains were minced and homogenized in mitochondrial lysis buffer to release mitochondria. The homogenates were spun at 800 × *g* for 10 min at 4 °C, and the resulting supernatants were spun at 12,000 × *g* for 20 min at 4 °C. The pellets were washed with lysis buffer and spun at 12,000 × *g* again for 20 min at 4 °C. The final pellets were suspended in lysis buffer containing 1% Triton X-100 and were mitochondrial-rich lysate fractions. The mitochondrial protein voltage-dependent anion channel (VDAC) was used as a loading control.

### Rat liver mitochondrial sub-compartmental fractionations

Isolation of mitochondrial sub-compartmental fractionations including contact sites was described previously^50^. Briefly, liver from one rat (male Lewis, 370 g) was used and mitochondria isolated by differential centrifugation and purified further by a Percoll gradient centrifugation. The mitochondrial pellet was slowly resuspended in 70 ml 20 mM potassium phosphate/ 0.2% defatted BSA, pH 7.2, and then incubated on ice for 20 min with gentle stirring to induce swelling and rupture of the mitochondrial outer membrane. After 20 min, ATP (0.5 M) and MgCl_2_ (1 M) were added to a final concentration of 1 mM each and the mitochondrial suspension was incubated for an additional 5 min on ice with stirring. The swollen/shrunk mitochondria were centrifuged for 20 min at 4 °C at 22,550 × *g* (Sorvall RC5C, SS-34 rotor), and the pellet was slowly resuspended in 70 ml 20 mM potassium phosphate/0.2% defatted BSA, pH 7.2. The mitochondrial suspension was treated with two strokes of a tight-fitting pestle (Potter–Elvjehem, setting 3) and centrifuged for 15 min at 4 °C with 1900 × *g* (Sorvall RC5C, SS-34 rotor). The supernatant was carefully removed and centrifuged for 20 min at 22,550 × *g* (Sorvall RC5C, SS-34 rotor) to obtain the crude outer membrane pellet. The pellet was resuspended in 0.6 ml of 20 mM potassium phosphate, pH 7.2 (13–17 mg protein/ml), using a hand-driven Potter homogenizer and separated into outer membranes, contact site fraction, and inner membranes by discontinuous sucrose gradient centrifugation.

The gradient was 1.2 ml each of 51.3, 37.7, and 25.2% sucrose in 20 mM potassium phosphate, pH 7.2; 0.8 ml (10.5–13.6 mg protein) of crude outer membrane fraction was loaded per tube and centrifuged for 60 min at 4 °C and 121,000 × *g* (Beckman L8-M ultracentrifuge, SW 50.1 rotor). The membranous material at the 25.2%/37.7% (purified mitochondrial outer membranes) and 37.7%/51.2% (contact sites) interfaces were collected, diluted tenfold with cold 20 mM potassium phosphate, pH 7.2, recovered by centrifugation for 1 h at 184,000 × *g* (Beckman L8-M ultracentrifuge, 50.2Ti rotor), and resuspended in a small volume of 20 mM potassium phosphate, pH 7.2. The pellet at the bottom of the tube from the discontinuous sucrose gradient was resuspended in 20 mM potassium phosphate, pH 7.2 and used for analysis.

### In situ proximity ligation assay (PLA)

HdhQ7 and HdhQ111 were grown overnight on glass coverslips. After washing with PBS, the cells were fixed with 4% paraformaldehyde for 20 mins at room temperature, permeabilized with 0.1% Triton X-100 in PBS for 5 min, and blocked with PLA blocking buffer for 1 h at 37 °C. The cells were then subject to the PLA using Duolink® In Situ Red Starter Kit Mouse/Rabbit assay kit (DUO92101, Sigma) according to the manufacturer’s instructions. Briefly, after blocking, the cells were incubated with rabbit anti-ATAD3A (1:500, Abcam, ab112572) and mouse anti-Mitofilin or mouse anti-Cytochrome *C* antibodies overnight at 4 °C. The cells were then incubated with the PLA probes (Anti-Rabbit PLUS and Anti-Mouse MINUS) for 1 h at 37 °C, followed by the ligation and amplification. In this assay, the oligonucleotides hybridize to the two PLA probes and join to a closed loop if they are in close proximity. Amplification solution, consisting of nucleotides and fluorescently labeled oligonucleotides, was added together with polymerase. The oligonucleotide arm of one of the PLA probes acts as a primer for “rolling-circle amplification” (RCA) using the ligated circle as a template, and this generates a conca-temeric product. Fluorescently labeled oligonucleotides hybridize to the RCA product. The PLA signal was visible as a distinct fluorescent spot and was analyzed by confocal microscopy (Fluoview FV1000, Olympus). The number of fluorescent signals was then quantitated using NIH Image J software.

### Immunogold electron microscopy

The cultured HdhQ7 and HdhQ111 cells were processed for immunocytochemistry and examined as previously described^51^. Grids were then incubated with the monoclonal antibody (anti-ATAD3A, Abcam ab112572) at 1:5, 1:25, and 1:50 dilution in phosphate buffered saline (PBS) containing 1% w/v bovine serum albumin and 0.01% v/v Tween 20 (PBT) for 12 h at 4 °C. Negative controls included normal rabbit serum and PBT replaced as the primary antibody. After washing, grids were incubated for 2 h in 10 nm gold-conjugated goat anti-rabbit IgG (BBInternational, Ted Pella, CA) diluted 1:30 in PBT, rinsed with PBS, and fixed with glutaraldehyde to stabilize the gold particles. For the enhancement of the mitochondria membrane, the grids were treated with the vapor fixation from osmium tetroxide. Samples were then stained with acidified uranyl acetate and Sato’s triple lead stain^52^, and examined in an FEI Tecnai Spirit (T12) with a Gatan US4000 4kx4k CCD.

### Mass spectrometry analysis of acetylated lysine residues

Total lysates of HdhQ7 and HdhQ111 cells were IP with anti-ATAD3A antibodies. The ATAD3A immunoprecitates were subject to SDS-PAGE analysis followed by Coomassie Brilliant blue staining. The SDS-PAGE gel was then submitted for tryptic and chymotryptic digestion and LC-MS/MS analysis. Briefly, gel bands were manually excised. Gel bands were then cut to minimize excess polyacrylamide, divided into a number of smaller pieces. The gel pieces washed with water and dehydrated in acetonitrile. The bands were then reduced with DTT and alkylated with iodoacetamide prior to the in-gel digestion. All bands were digested in-gel using trypsin, by adding 5 μL 10 ng/μL trypsin in 50 mM ammonium bicarbonate and incubating overnight digestion at r.t. to achieve complete digestion. The peptides that were formed were extracted from the polyacrylamide in two aliquots of 30 μL 50% acetonitrile with 5% formic acid. These extracts were combined and evaporated to <10 μL in Speedvac and then resuspended in 1% acetic acid to make up a final volume of ~30 μL for LC-MS analysis. The LC-MS system was a ThermoScientific Fusion Lumos tribrid mass spectrometer system. The HPLC column was a Dionex 15 cm × 75 μm id Acclaim Pepmap C18, 2 μm, 100 Å reversed-phase capillary chromatography column. Five μL volumes of the extract were injected and the peptides eluted from the column by an acetonitrile/0.1% formic acid gradient at a flow rate of 0.3 μL/min were introduced into the source of the mass spectrometer on-line. The microelectrospray ion source is operated at 2.5 kV. The digest was analyzed using the data dependent multitask capability of the instrument acquiring full scan mass spectra to determine peptide molecular weights and product ion spectra to determine amino acid sequence in successive instrument scans. The data were analyzed by using all CID spectra collected in the experiment to search the mouse or human UniProtKB with the search programs Sequest and Mascot. For post-translational modification analysis, the data from these bands was searched considering acetylation. In addition, a second LC-MS/MS experiment was carried out that corresponds to a parallel reaction monitoring (PRM) experiment which involves fragmentation of specific *m*/*z* ratios over the entire course of the LC experiment.

### Real-time PCR

Total RNA was isolated using RNeasy Mini Kit (QIAGEN), and 0.5–1 µg of total RNA was used to synthesize cDNA using QuantiTect Reverse Transcription Kit (QIAGEN). qRT-PCR was performed using QuantiTect SYBR Green (QIAGEN) and analyzed with the StepOnePlus Real-Time PCR System (Thermo Fisher Scientific). Three replicates were performed for each biological sample, and the expression values of each replicate were normalized against GAPDH cDNA using the 2^-ΔΔCT^ method. The primers used were as follows (5’−3’): mGAPDH-s: GACTTCAACAGCAACTCCCAC; mGAPDH-as: TCCACCACCCTGTTGCTGTA. mTFAM-s: AAGGATGATTCGGCTCAGG; mTFAM-as: GGCTTTGAGACCTAACTGG. mPOLRMT-rt-s: GCAACATGTCCTGAGGGAGT; mPOLRMT-rt-as: GCACCTTCTTCACCCTCATC. mPOLG-rt-s: ACGTGGAGGTCTGCTTGG; mPOLG-rt-as: AGTAACGCTCTTCCACCAGC. mTwink-rt-s: GTCATTCACCCTCGGAAAGA; mTwink-rt-as: GCCCAGTCACCAGTTTCCTA. mSSBP1-rt-s: TGTCCGGAAAAGCCTAAAGA; mSSBP1-rt-as: CCAAACTGCTGGCTACTTCA. mATAD3A-rt-s: AGCTAACGGAGGGGATGTCA; mATAD3A-rt-as: GCATCCATCATAGCTTCCGT.

### Measurement of mtDNA content

Total DNA was isolated from cells using Blood and Cell culture kit (QIAGEN), and then subject to qPCR using QuantiTect SYBR Green (QIAGEN). Quantitative PCR was performed using nuclear primers (GAPDH or Tert) and mtDNA primers (mt-CO2, mt-ND2, mt-CO1 and D-loop). Three technical replicates were performed for each biological sample, and the mtDNA abundance of each replicate were normalized against nuclear DNA using the 2^−ΔΔCT^ method.

For isolation of DNA from mouse plasma, the QIAamp DNA Blood Mini Kit (QIAGEN) was used. Briefly, 100–200 µl mouse plasma was subjected to total DNA extraction. Then the eluted DNA was diluted (1:5) to perform the qPCR experiment.

The related primers used are listed as follows: m-nucDNA GAPDH-DNA-s: GGACCTCATGGCCTACATGG; m-nucDNA GAPDH-DNA-as: TAGGGCCTCTCTTGCTCAG. m-mtCO2-s: TAGGGCACCAATGATACTGAAG; m-mtCO2-as: CTTCTAGCAGTCGTAGTTCACC. m-mtND2-s: AACCCACGATCAACTGAAGC; m-mtND2-as: TTGAGGCTGTTGCTTGTGTG. m-mtCO1-s: CTGAGCGGGAATAGTGGGTA; m-mtCO1-as: TGGGGCTCCGATTATTAGTG. m-mtDNA D loop1-s: AATCTACCATCCTCCGTGAAACC; m-mtDNA D loop1-as^40^: TCAGTTTAGCTACCCCCAAGTTTAA. m.mtDNA D loop2-s: CCCTTCCCCATTTGGTCT; m-mtDNA D loop2-as: TGGTTTCACGGAGGATGG. m-mtDNA D loop3-s: TCCTCCGTGAAACCAACAA; m-mtDNA D loop3-as: AGCGAGAAGAGGGGCATT. m-nucDNA Tert-s: CTAGCTCATGTGTCAAGACCCTCTT; m-nucDNA Tert-as: GCCAGCACGTTTCTCTCGTT.

### TFAM-mtDNA binding assay

Total lysates were isolated from cells with the indicated treatments, and then incubated with biotinylated LSP DNA probe (Biotin-LSP) or LSP (control) overnight at 4 °C. Then streptavidin agarose beads (Thermo Fisher Scientific) were added for 1 h at 4 °C. After incubation, the biotin-streptavidin complex was washed 3–5 times (1 ml/time) at 4 °C and subject to western blot analysis. To validate the LSP binding specificity to TFAM, 10 times (molar ratio) of non-labeled LSP was pre-incubated with an equal amount of lysate for 2 h prior to addition of the biotin-LSP probe.

The biotinylated LSP DNA probe is a double stranded DNA oligo containing a TFAM consensus sequence derived from the LSP^53^. The following related DNAs were used: Biotin-LSP-s: 5′-biotin-tgtgttagttggggggtgactgttaaa-3′; LSP-s: 5′ -tgtgttagttggggggtgactgttaaa-3′; and LSP-as: 5′-tttaacagtcaccccccaactaacaca-3′.

### Measurement of mtDNA lesion by Quantitative PCR

Because the movement of the polymerase on the template is blocked at a lesion, qPCR amplification is inversely proportional to the presence of DNA damage. The level of mtDNA lesion will be calculated by comparing the 10 kb mtDNA fragment amplification, as described previously^54,55^. To correct for the possible variations in the rate of mtDNA replication, the amplification of 10 kb mtDNA fragment was normalized to the 91 bp small mtDNA amplicon. Specifically, Total DNA was isolated as shown above. The amplification profile consisted of a 45-s denaturation at 94 °C, 22 cycles of denaturation for 15 s at 94 °C, annealing/extension at 64 °C for 12 min, and a final extension at 72 °C for 10 min. The primer nucleotide sequences used for the amplification of the 10 kb mouse mitochondrial fragments are listed as follows: m-mtDNA-Large-s: 5-CCAGTCCATGCAGGAGCATC-3; m-mtDNA-Large-as: 5-CGAGAAGAGGGGCATTGGTG-3. The primers used for the amplification of the 91 bp mouse mitochondrial fragments are listed as follows: m-mtDNA-91bp-s: 5-CCCAGCTACTACCATCATTCAAGT-3; m-mtDNA-91bp-as: 5-GATGGTTTGGGAGATTGGTTGATGT-3. The primer used for the amplification of the mouse nDNA fragment are listed as follows: nDNA-large-s: 5-CCACCAGGCGTCACCCTTGA-3; nDNA-large-as: 5-TGGGAGGCAGGGATCTGAAGC-3. All the related mtDNA lesion results were quantified from three qPCR experiments.

### Immunofluorescence analysis

Cells grown on coverslips were fixed with 4% paraformaldehyde for 20 mins at r.t., permeabilized with 0.1% Triton X-100 in PBS and blocked with 2% normal goat serum. The cells were then incubated with primary antibody (overnight, at 4 °C) followed by Alexa Fluor 568 or 488 secondary antibody (2 h, r.t.). After staining with Nuclei with Hoechst dye (1:10,000 dilution), the cells were mounted onto slides. The cells were imaged by Fluoview FV1000 microscopy (Olympus).

To examine mitochondrial superoxide production, the cells were stained with MitoSOX (Invitrogen, 5 µM for 10 min) at 37 °C. The cells were then washed with PBS three times and mounted for the image analysis. At least 150 cells/group were counted and quantitated.

Neurons derived from iPS cells were stained with anti-Tom20 (a mitochondrial marker) and anti-GAD67 (a striatal neuronal marker). Quantitation of mitochondrial length along the neurites was conducted only in the neurons immuno-positive for GAD67. To analyze dendrite and axon outgrowth of neurons, cells were stained with anti-MAP2 (a dendritic marker) and anti-GAD67 or anti-Tau (an axonal marker) and anti-DARPP32 (a medium spiny neuron marker). The length of MAP2^+^ dendrite and Tau^+^ axon in the neurons immuno-positive for GAD67 or DARPP32 was quantitated, respectively. At least 50 neurons/group were counted.

For Iba1 immunofluorescence staining, mice were deeply anesthetized and transcardially perfused with 4% paraformaldehyde in PBS. Frozen brain sections (10 μm, coronal) were permeabilized with 0.1% Triton X-100 in TBS-T buffer followed by blocking with 5 % normal goat serum. The brain sections were then incubated with anti-Iba1 antibody (1:500 dilution) for overnight at 4 °C, followed by staining with Alex-488 anti-rabbit secondary antibody (1:2000 dilution). Iba1-immunopositive cells in the 100 mm^2^ were quantitated by image J software.

### LDH assays

Cells were treated as described, and cell death was determined by using Cytotoxicity Detection Kit (LDH) according to protocols recommended by the manufacturer (Roche).

### Rational design of peptide inhibitor

In a similar approach to the peptide design for Drp1 peptide P110^56^ and VCP peptide inhibitor HV-3^31^, we used L-ALIGN sequence alignment software and identified one region of homology between Drp1 (human, NP_036192) and ATAD3A (human, NP_001164006). These regions are marked as regions DA1 and DA2, respectively. All the homologous sequences are conserved across a variety of species, including human, mouse, rat, and bovine. The peptides were synthesized at Ontores (Hangzhou, China) corresponding to regions DA1 and DA2 and conjugated to the cell permeating TAT protein-derived peptide, TAT_47–57_. These peptides are referred to as DA1 (TAT-GG-EDKRKT-NH2, Product number P103882, Lot# 0P082714SF-01) and DA2 (TAT-GG-EERRKT-NH2, Product number: P103883. Lot#: 0P0827714SF-02). Note that TAT_47–57_–based delivery was used in culture and in vivo and was found to be safe and efficacious for delivery of peptide cargoes to cells and also to cross the blood—brain-barrier^7,31,56^. The purity was assessed as >90% by mass spectrometry. Lyophilized peptides were dissolved in sterile water and stored at −80 °C until use.

### 3D modeling and rendering of protein structure

The 3D structural model of the ATAD3A N-terminal segment (residues 1–210) (GenBank: AAH63607.1) was predicted by I-TASSER^57^ and graphically rendered using the PyMol Molecular Graphics System, version 1.7.4.0, Schrodinger, LLC. The 3D crystal structure of the Drp1 GTPase-GED fusion construct (PDB ID: 4H1U) was similarly rendered using PyMol. DA1 and DA2 were mapped on Drp1 and ATAD3A, respectively. The K135 was labeled in pink in the ATAD3A N-terminal structural model.

### Measurement of mitochondrial respiratory activity

Mitochondrial respiration activity in HdhQ7 and HdhQ111 cells was analyzed using a Seahorse Bioscience XFp Extracellular Flux Analyzer. The cells were seeded in XFp 6-well microplates at 6,000 cells per well in 50 µl of growth medium. One hour prior to measuring oxygen consumption, the cell culture media was replaced with XF assay medium and maintained in a non-CO_2_ incubator for 1 h. The sensor cartridges were placed in the XFp Analyzer according to the manufacturer’s instructions for the Mito Stress Test kit. In brief, mitochondrial function was determined by the sequential injection of oligomycin A (2.5 µg/mL), FCCP (1 µM), and antimycin A (4 µM). Following each experiment, the total cellular number of each well was determined using DAPI staining.

### Immunoprecipitation

Cells and mouse brain tissues were harvested and lysed in total lysis buffer (10 mM HEPES-NaOH, pH 7.9, 150 mM NaCl, 1 mM EGTA, 1% Triton X-100, and protease inhibitor cocktail) for 30 mins on ice and centrifuged at 12,000 × *g* for 10 min at 4 °C. The supernatants were incubated with the indicated primary antibodies or control IgG (sc-2025 and sc-2027, Santa Cruz Biotechnology) overnight at 4 °C, followed by incubation with protein A/G beads (sc-2003, Santa Cruz Biotechnology) for 2 h at 4 °C. The immunoprecipitates were washed with lysis buffer three times for a total of 30 min and then subject to WB analysis.

For Drp1-Mff or Drp-MiD51 (MIEF1) interaction, the cells were cross-linked by 1 mM DSP for 10 min at r.t., then 10 mM Glycine in PBS was added to quench the redundant DSP activity. For Drp1-Fis1 interaction, the cells were cross-linked by 1% FA for 20 min at r.t., then added 10 mM Glycine in PBS to quench the redundant activity. The cells were washed with PBS, sonicated, and lysed with 1% Triton X-100 total lysis buffer.

For biotin-DA1 binding assay, biotin-DA1 and biotin-TAT (10 µM, each) were incubated with total lysates of cell cultures for overnight at 4 °C followed by the incubation with streptavidin beads for 1 h. Immunoprecipitates were washed four times with cell lysate buffer and were analyzed by SDS-PAGE and immunoblotting.

### Preparation of ATAD3A protein

Using pcDNA3.1 (+)-ATAD3A-Flag plasmid as template, ATAD3A-Flag protein was synthesized by TNT® Quick Coupled Transcription/Translation System (Promega, Cat# L1170) followed by protein purification with Flag-peptide (Sigma, F3290). The expression of ATAD3A protein was confirmed by western blot analysis with both anti-ATAD3A and anti-Flag antibodies. This approach provides convenient single-tube, coupled transcription/translation reactions for eukaryotic cell-free protein expression and has been used to detect direct protein–protein interaction^58,59^.

### GST pulldown assay

GST-Drp1 or GST control protein were incubated with Glutathione-beads in TBST buffer at r.t. After 1 h of incubation, ATAD3A-Flag protein was added, and thoroughly mixed in vibrating mixer for 30 mins at r.t., then transferred to 4 °C for overnight incubation. Immunoprecipitates were then washed for three time and analyzed by SDS-PAGE.

To test the inhibitory function of DA1 peptide on in vitro binding between GST-Drp1 and ATAD3A-Flag, DA1 peptide or control peptide TAT (60 µM, each) was incubated with ATAD3A-Flag protein before mixing with GST-Drp1-glutathione beads complex.

### In vitro binding assay

GST-Drp1 and ATAD3A-Flag proteins (2 µg, each) were incubated in the buffer (20 mM HEPES [pH 7.4], 100 mM KCL, 2 mM MgCl2) for 30 min at RT, and followed with Flag antibody incubation at 4 °C for overnight. The mixture was then incubated with protein A/G beads for 2 h followed by immunoprecipitation. Note that no crosslinker was added in these in vitro binding assays.

For biotin-DA1 binding assay, biotin-DA1 and biotin-TAT (10 µM, each) were incubated with GST-Drp1 or ATAD3A-Flag protein for overnight at 4 °C followed by the incubation with streptavidin beads for 1 h. Immunoprecipitates were washed four times with cell lysate buffer and were analyzed by SDS-PAGE and immunoblotting.

Drp1 GTP hydrolysis was monitored as a function of time as described previously^24,60^.

### Western blot analysis

Protein concentration was measured by protein assay dye reagents (Bio-rad). The protein was resuspended in Laemmli buffer, loaded on SDS-PAGE, and transferred onto nitrocellulose membranes. Membranes were probed with the indicated antibodies, followed by visualization with ECL. Representative blots have been cropped for presentation. Images of full-size blots are presented in Supplementary Fig. 9.

### Animal models of HD

All animal experiments were conducted in accordance with protocols approved by the Institutional Animal Care and Use Committee of Case Western Reserve University and were performed based on the National Institutes of Health Guide for the Care and Use of Laboratory Animals. Sufficient procedures were employed for reduction of pain or discomfort of subjects during the experiments.

Male R6/2 mice and their wild-type (WT) littermates (5 weeks old) were purchased from Jackson Laboratories [Bar Harbor, ME; B6CBA-TgN (HD exon1)62; JAX stock number: 006494]. These mice (C57BL/6 and CBA genetic background) are transgenic for the 5’ end of the human HD gene carrying 100–150 glutamine (CAG) repeats.

YAC128 [FVB-Tg(YAC128)53Hay/J, JAX stock number: 004938] breeders (FVB/N genetic background) were purchased from Jackson Laboratories. The YAC128 mice contain a full-length human huntingtin gene modified with a 128 CAG repeat expansion in exon 1. The mice were mated, bred, and genotyped in the animal facility of Case Western Reserve University. Male mice at the ages of 2, 3, 6, 9, and 12 months were used in the study.

All mice were maintained at a 12-hour light/dark cycle (on 6 am, off 6 pm).

### Systemic peptide treatment in HD mice

All randomization and peptide treatments were prepared by an experimenter not associated with behavioral and neuropathology analysis. Male R6/2 mice (Tg) and their age-matched wild-type littermates (Wt) (6-week-old) were implanted with a 28-day osmotic pump (Alzet, Cupertino CA, Model 2004) containing TAT control peptide or DA1 peptide, which delivered peptides at a rate of 1 mg/kg/day. Male YAC128 mice (Tg) and their age-matched wild-type littermates (WT) were treated with TAT control peptide or DA1 peptide (1 mg/kg/day) by Alzet osmotic pump starting from the age of 3 months. The pump was replaced once every month.

### Behavioral analysis in HD mice

All behavioral analyses were conducted by an experimenter who was blinded to genotypes and treatment groups.

Gross locomotor activity was assessed in R6/2 mice and age-matched wild-type littermates at the ages of 12 weeks, and in YAC128 mice and age-matched wild-type littermates at the ages of 2, 3, 6, 9, and 12 months. The mice were placed in the center of an activity chamber (Omnitech Electronics, Inc) and allowed to explore while being tracked by an automated beam system (Vertax, Omnitech Electronics Inc). Distance moved, horizontal and vertical activities were recorded. In R6/2 mice and wildtype littermates, one-hour locomotor activity was conducted. In YAC128 mice and wildtype littermates, 24 h of locomotor activity analysis was performed.

Hindlimb clasping was assessed with the tail suspension test once a week from the ages 8 to 12 weeks in R6/2 mice. The mice were suspended by the tail for 60 s, and the latency for the hindlimbs or all four paws to clasp was recorded using the score system:^61^ Clasping over 10 s, score 3; 5–10 s, score 2; 0–5 s, score 1; and 0 s, score 0.

The body weight and survival rate of HD mice and wild-type littermates were recorded throughout the study period.

### Immunohistochemistry

Mice were deeply anesthetized and transcardially perfused with 4% paraformaldehyde in PBS. Brains were processed for paraffin embedment. Brain sections (10 μm, coronal) were used for immunohistochemical localization of DARPP-32 (1:500, Abcam) or ATAD3A (1:200, Genetex) using the IHC Select HRP/DAB kit (Millipore). Quantitation of DARPP-32 immunostaining was conducted using NIH image J software. The same image exposure times and threshold settings were used for sections from all treatment groups. An experimenter blinded to the experimental groups conducted the quantitation.

### Striatum volume

Coronal sections (10 µm) spaced 200 µm apart throughout the striatum will be stained with anti-NeuN (Sigma, MAB377, 1:200 dilution) antibody followed by secondary antibodies and detection with diaminobenzidine (DAB, Pierce). The perimeter of the striatum will be traced in each of the serial sections using a ×2 objective. For the volume estimation, consecutive sections (an average of 10–15 sections/animal) will be visualized and the perimeter of the striatum will be outlined. The striatal volume will be estimated by multiplying the sum of all the sectional areas (mm^2^) by the distance between successive sections (200 μm).

### Dendrite morphology of medium spiny neuron in mice

Dendrite morphology of medium spiny neurons in wildtype and HD R6/2 mice was analyzed by Golgi-Cox staining using Novaultra Golgi-Cox Stain Kit (IHCWorld, SKU IW-3023). Briefly, mice were deeply anesthetized and transcardially perfused with 4% paraformaldehyde in PBS. After thoroughly washing with PBS overnight, mouse brains were immersed in the Golgi-Cox solution in dark at room temperature, then replaced with fresh Golgi-Cox Solution after 2 days of immersion and continued the impregnation at room temperature in dark for 14 days following the manufactory instruction. After washing with PBS for 2 days, serial coronal sections of 200 µm thick were cut on Vibratome Series 1000 Sectioning System. The coronal sections were washed with water and stained with Post-Impregnation Solution for 10 min in dark at room temperature. Following the 3 times of washing with water, the brain sections were mounted on Superfrost plus slides (Thermo Scientific). Medium spiny neurons in striatum were selected for analysis. Total dendritic length per neuron was measured using NIH Image J plug-in simple neurite tracer.

### Statistical analysis

Sample sizes were determined by a power analysis based on pilot data collected by our labs or published studies. In the animal studies, we used *n* = 15–20 mice/group for behavioral tests, *n* = 14–15 mice/group for recording survival rate and body weight, *n* = 6–9 mice/group for biochemical analysis, and *n* = 6 mice/group for pathology studies. In the cell culture studies, we performed each study with at least three independent replications. For all of the animal studies, we ensured randomization and blinded conduct in experiments. For all imaging analyses, an observer who was blind to the experimental groups conducted the quantitation. No samples or animals were excluded from the analysis.

Data were analyzed by Student’s *t* test for comparison of two groups or ANOVA with post-hoc Tukey’s test for comparison of multiple groups. Survival rate was analyzed by Log-rank (Mantel-Cox) test and behavioral tests were analyzed by repeated-measure two-way ANOVA. Data are expressed as mean ± SEM. Statistical significance was considered achieved when the value of *p* was < 0.05.

## Supplementary information

Supplementary Information

Source Data

## Data Availability

The datasets generated under the current study are available from the corresponding author upon reasonable request. All statistical resource data are presented in Source Data File. The proteomic database was submitted to figshare (https://figshare.com/) 10.6084/m9.figshare.7763459 [https://figshare.com/s/aebb3df638e35a035bd3].
