# Supplementary material for: ATAD3A oligomerization causes neurodegeneration by coupling mitochondrial fragmentation and bioenergetics defects
Source: Nat Commun. 2019 Mar 26;10:1371. doi: 10.1038/s41467-019-09291-x (PMC6435701; doi:10.1038/s41467-019-09291-x)

Supplementary information

ATAD3A oligomerization causes neurodegeneration by coupling mitochondrial fragmentation and bioenergetics defects

Yuanyuan Zhao[1], Xiaoyan Sun[1], Di Hu[1], Domenick A. Prosdocimo[2,3] Charles Hoppel[4,5], Mukesh K. Jain[2,3], Rajesh Ramachandran[1] and Xin Qi[1,4*]

[1]Department of Physiology & Biophysics, [2]Case Cardiovascular Research Institute and Harrington Heart & Vascular Institute, [3]Department of Medicine, University Hospitals Case Medical Center, [4]Center for Mitochondrial Disease, [5]Department of Pharmacology, Case Western Reserve University School of Medicine, Cleveland, OH 44106, USA.

***Corresponding author:**
Xin Qi PhD, Department of Physiology and Biophysics, Case Western Reserve University School of Medicine, 10900 Euclid Ave, E516, Cleveland, Ohio, 44106-4970, USA. Tel: 216-368-4459; Fax: 216-368-5586; E-mail: xxq38@case.edu

Supplementary Figure 1

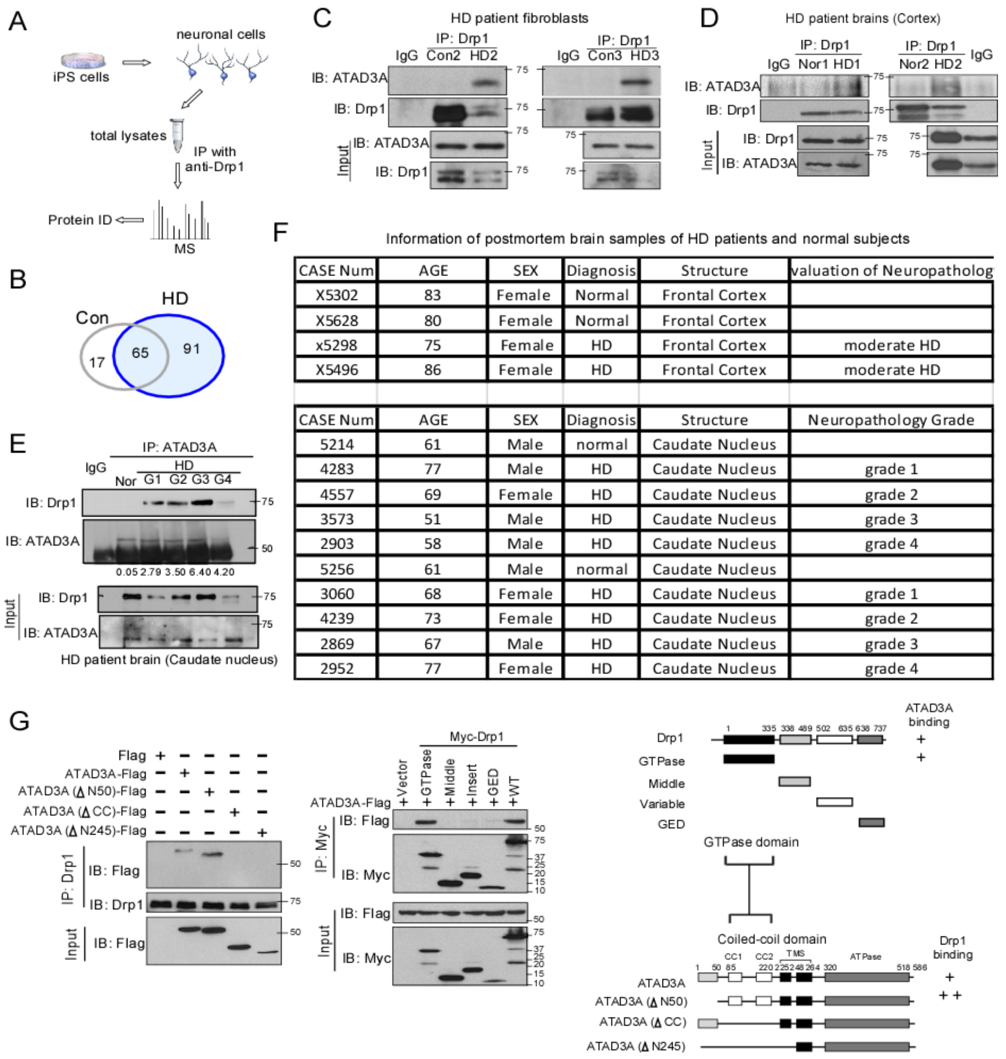

CASE Num	AGE	SEX	Diagnosis	Structure	valuation of Neuropatholog
X5302	83	Female	Normal	Frontal Cortex	
X5628	80	Female	Normal	Frontal Cortex	
x5298	75	Female	HD	Frontal Cortex	moderate HD
X5496	86	Female	HD	Frontal Cortex	moderate HD

CASE Num	AGE	SEX	Diagnosis	Structure	Neuropathology Grade
5214	61	Male	normal	Caudate Nucleus	
4283	77	Male	HD	Caudate Nucleus	grade 1
4557	69	Female	HD	Caudate Nucleus	grade 2
3573	51	Male	HD	Caudate Nucleus	grade 3
2903	58	Male	HD	Caudate Nucleus	grade 4
5256	61	Male	normal	Caudate Nucleus	
3060	68	Female	HD	Caudate Nucleus	grade 1
4239	73	Female	HD	Caudate Nucleus	grade 2
2869	67	Male	HD	Caudate Nucleus	grade 3
2952	77	Female	HD	Caudate Nucleus	grade 4

Supplementary Figure 1: ATAD3A binds to Drp1 in HD models. (A) Proteomic analysis of Drp1 interactors in neuronal cells derived from HD patient-iPS cells. Neuronal cells were differentiated from HD patient- or normal subject-iPS cells for 40 days. Total protein lysates were subjected to immunoprecipitation (IP) with anti-Drp1 antibodies. The immunoprecipitates were eluted from beads followed by tandem mass spectrometry analysis. (B) Venn diagram showing the protein numbers and overlaps of Drp1 binding proteins in cells differentiated from iPS cells of HD patient and normal subject. Only candidates with spectra count over 3 will be analyzed. (C) Total protein lysates were harvested from HD patient or normal fibroblasts. HD2: GM05539, 10 years old, Male; HD3: GM21756, Female; Con2: nHDF (juvenile); Con3: Huf1822 (adult). (D) Total protein lysates were harvested from frozen postmortem cortex of HD patients and normal subjects. Nor 1: X5302; Nor2: X5628; HD1: X5298; HD2: X5496. See the information of subjects in (F). IP analysis was conducted to determine Drp1 and ATAD3A interaction. All show representative blots above are from at least 3 independent experiments. (E) Total lysates of frozen postmortem caudate nucleus of normal subjects and HD patients with different disease severity (grade 1 to grade 4, slight to severe) were subject to IP analysis. Nor: normal subject 5256; G1: HD 3060, grade 1; G2: HD 4239, grade 2; G3: HD 2869, grade 3; G4: HD 2952, grade 4. The number below the blots indicate the relative density of Drp1 in ATAD3A immunoprecipitates (Drp1/ATAD3A in IP fraction). See the information of subjects in (F). (F) The list of postmortem brain samples of HD patients and normal subjects used in Fig. 1G and Supplementary Fig. 1D, E. (G) Domain interaction between Drp1 and ATAD3A. Upper: HEK293 cells were expressed with the indicated ATAD3A-Flag truncated mutants for 48 hours. Lower: HEK293 cells were co-expressed with Myc-Drp1 deletion mutants and ATAD3A-Flag for 48 hours. Maps of ATAD3A truncated mutants and Drp1 deletion mutants are shown. The domain

regions of Drp1-ATAD3A interaction are labeled as ‘+’. Shown representative blots are from at least 3 independent experiments.

Supplementary Figure 2

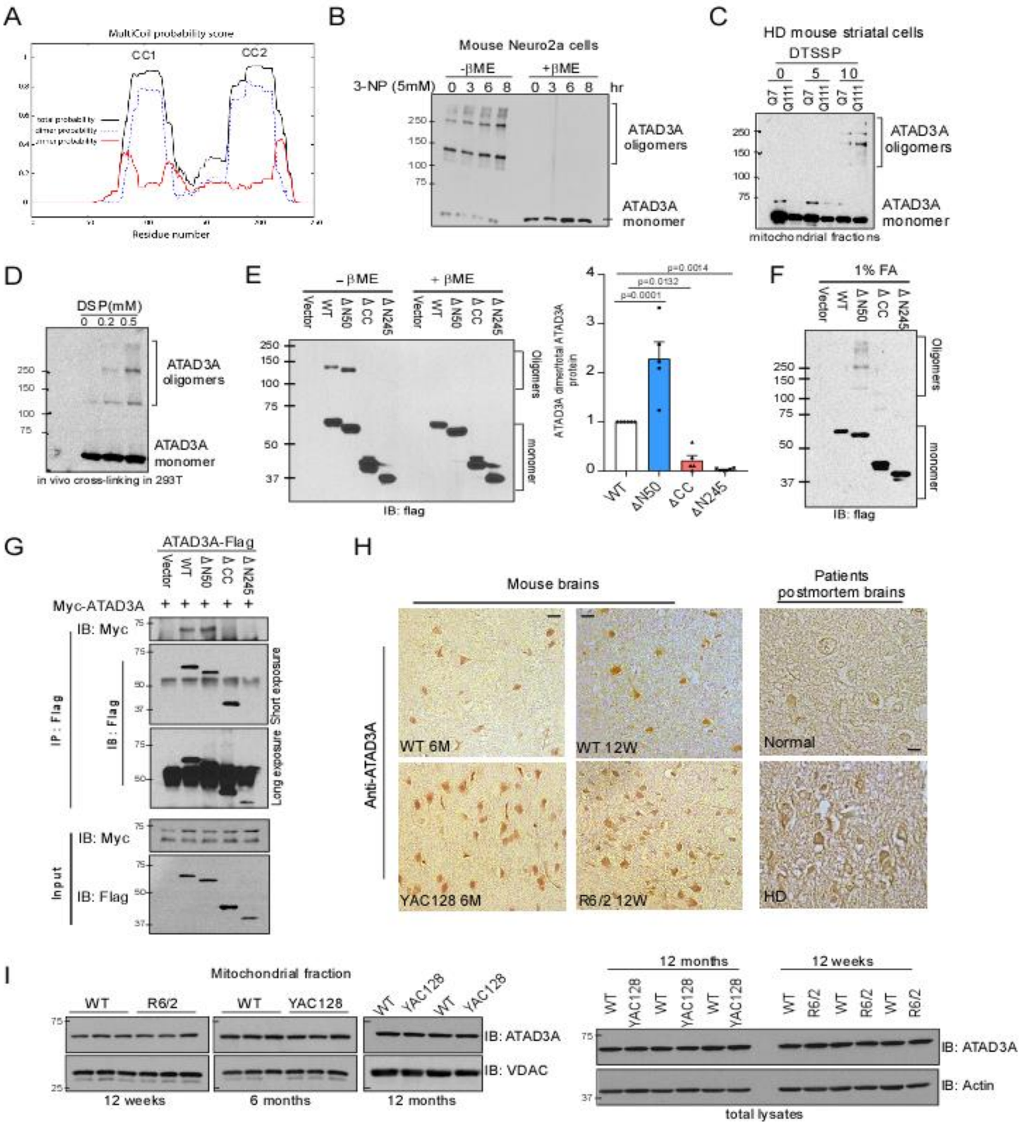

Supplementary Figure 2: ATAD3A forms oligomers in HD. (A) The presence and location of coiled-coil (CC) regions in the ATAD3A N-terminal sequence (residues 1-245) was predicted using the program MultiCoil. The potential of these regions to form dimeric or trimeric coiled-coils is indicated by a probability score that ranges from 0 to 1. (B) Neuro2a cells were treated with 3-NP (5mM) at the indicated times. ATAD3A oligomerization was analyzed by Western blot (WB) in the presence or absence of β-ME. (C) Mitochondria were isolated from HdhQ7 and Q111 cells followed by incubation with the crosslinker DTSSP (0.5 mM). (D) HEK293 cells were treated with crosslinker DSP for 5 min with the indicated doses. ATAD3A oligomers were analyzed by WB. (E) ATAD3A-Flag truncated mutants were expressed in HEK293 cells for 48 hours. ATAD3A oligomers were analyzed by WB with anti-Flag antibodies in the presence or absence of β-ME. Histogram shows the relative density of ATAD3A dimer versus the total level of ATAD3A in the presence of β-ME. Data are mean ± SEM of 5 independent experiments, one-way ANOVA with Tukey’s *post-hoc* test. (F) HEK293 cells expressing ATAD3A truncated mutants were incubated with 1% FA for crosslinking. WB was conducted. (G) HEK293 cells were co-expressed with ATAD3A-Flag truncated mutants and Myc-ATAD3A for 48 hours. Total protein lysates were subject to IP with anti-Flag antibodies followed by WB with the indicated antibodies. (H) Brain sections (10 μm) from YAC128 (6 months old),

R6/2 (12 weeks old) and age-matched wildtype mice, and formalin-fixed patient postmortem caudate nucleus (Nor: ID 624, 62 years old, Male; HD: ID 2983, 30 years old, Male) were stained with anti-ATAD3A antibodies. Scale bar: 10 µm. (I) The protein levels of ATAD3A in YAC128 (6 and 12 month) and R6/2 (12 weeks) mice. The total and mitochondrial levels of ATAD3A were analyzed by WB with protein lysates harvested from the striatum of YAC128 or R6/2 mice at the indicated ages. Actin: a loading control for the total lysates. VDAC: a mitochondrial loading control. Shown blots in B-D, F, G, I are representative of 3 independent experiments.

Supplementary Figure 3

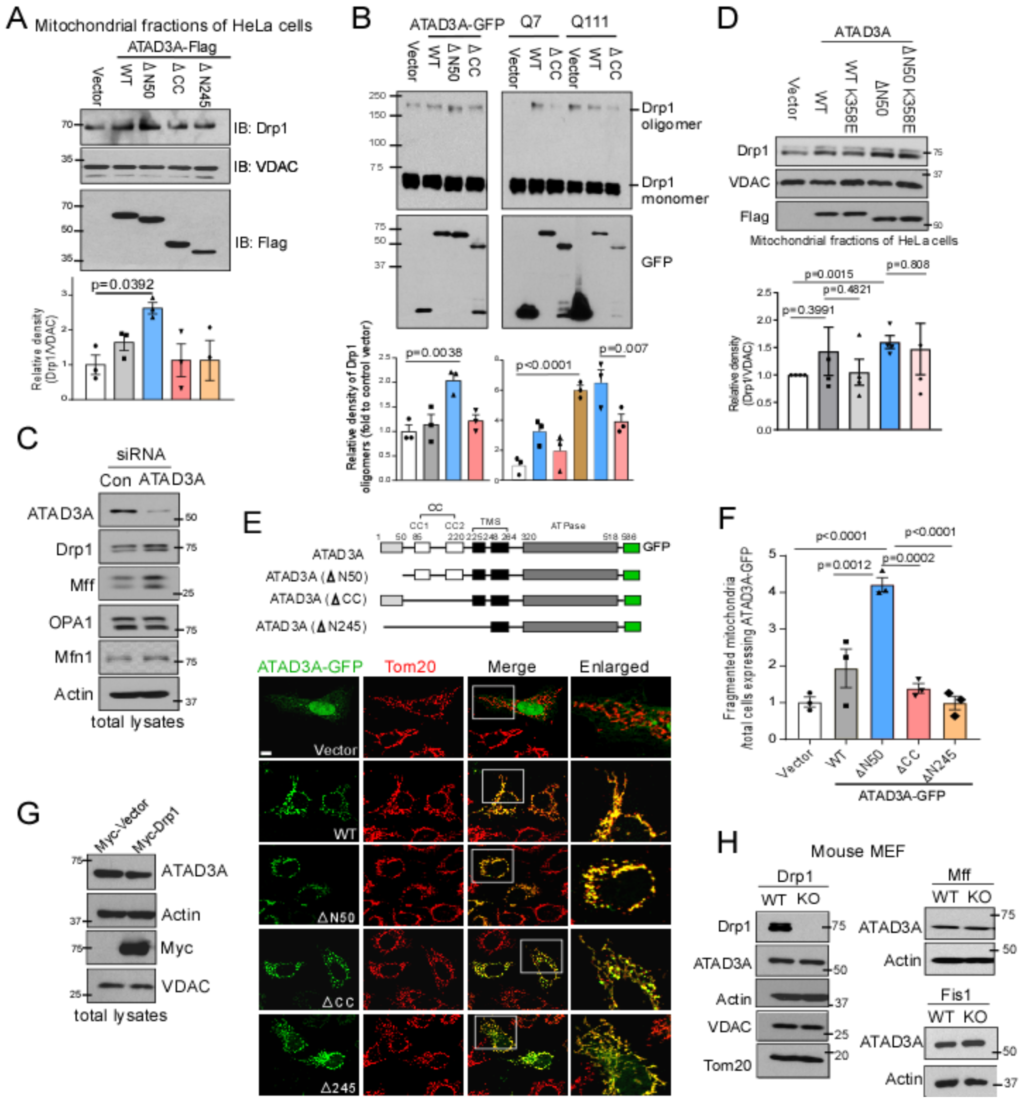

Supplementary Figure 3: Effects of ATAD3A on Drp1 activation. (A) HeLa cells were transfected with ATAD3A-Flag truncated mutants for 48 hours. Mitochondria were isolated and Drp1 translocation to mitochondria were determined by WB. Histogram: quantitation of relative Drp1 mitochondrial level. VDAC was used as a mitochondrial loading control. 3 independent experiments. (B) ATAD3A-GFP truncated mutants were expressed in wildtype mouse striatal cells (left) and HD striatal cells (right). Drp1 oligomerization in total protein lysates was analyzed by WB with anti-Drp1 antibody in the absence of β-ME. 3 independent experiments. (C) Wildtype striatal cells were transfected by control siRNA or ATAD3A siRNA for 80 hours. Mitochondrial fusion/fission related proteins were analyzed by WB with the indicated antibodies. Shown blots are representative of 3 independent experiments. (D) HeLa cells were transfected with ATAD3A wildtype (WT), WT-ATAD3A K358E, ATAD3A ΔN50 or ATAD3A ΔN50 K358E plasmids for 48 hours, and mitochondria were then isolated. Drp1 translocation to the mitochondria was determined by WB. VDAC was used as a mitochondrial loading control. Histogram: the

quantitation of relative Drp1 mitochondrial level to VDAC. 4 independent experiments. (E) HeLa cells were transfected with ATAD3A-GFP truncated mutants, shown in upper panel, for 48 hours. Cells were stained with anti-Tom20 antibodies (red) and mitochondria morphology was imaged. Scale bar: 10 µm. (F) The quantitation of the number of cells with fragmented mitochondria. ~200 cells/group were analyzed. 3 independent experiments. (G) HEK293 cells were expressed with Myc vector or Myc-Drp1 for 48 hours. ATAD3A protein level was analyzed by WB. Actin was used as a loading control. Shown blots are representative from 3 independent experiments. (H) ATAD3A protein level was analyzed by WB in Drp1-, Mff- or Fis1-knockout (KO) MEFs. Actin was used as a loading control. Shown blots are representative from 3 independent experiments. Data are mean ± SEM. One-way ANOVA with Tukey's *post-hoc* test.

Supplementary Figure 4

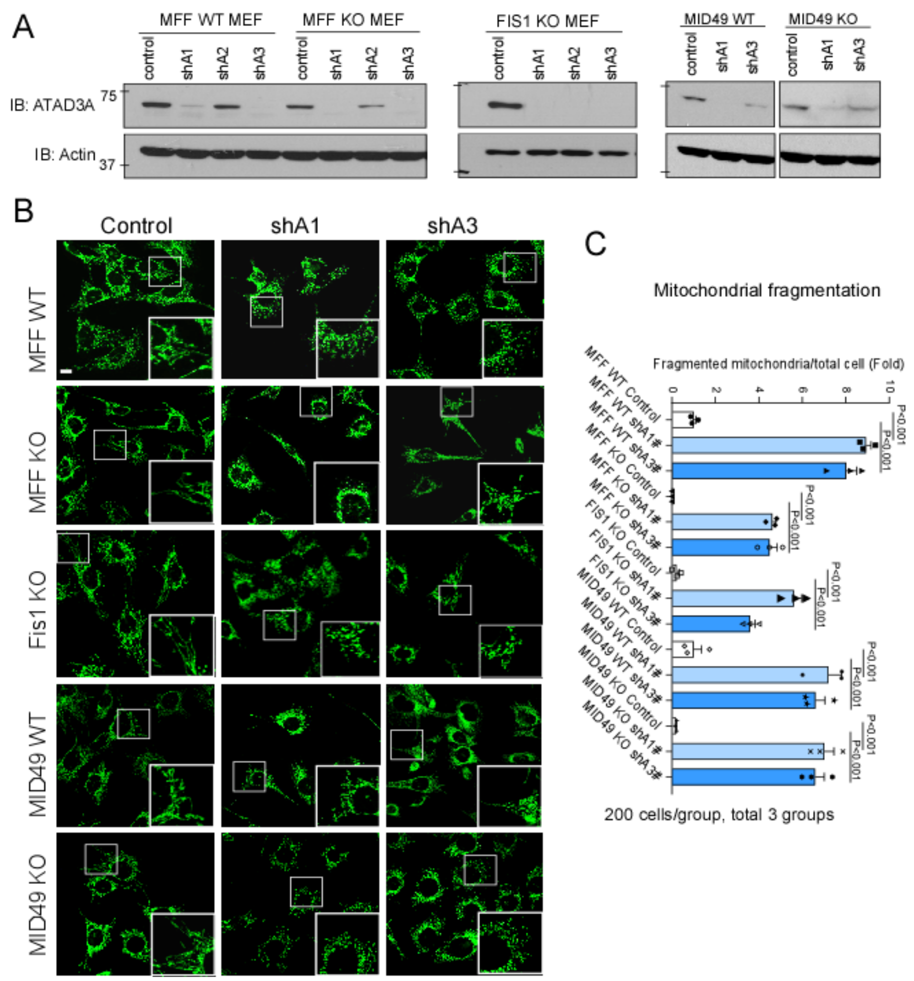

Supplementary Figure 4: Effects of ATAD3A knockdown on mitochondrial morphology in mitochondrial fission adaptors knockout (KO) MEFs. MFF-, Fis1- or MiD49-knockout (KO) MEFs and wildtype MEFs were infected with lentivirus containing ATAD3A shRNAs or control shRNA for 2 days and maintained in the cell culture medium containing puromycin (1 µg/ml). (A) Total cell lysates at the indicated groups were harvested, and the protein level of ATAD3A was determined by WB with anti-ATAD3A antibody. Actin was used as a loading control. ATAD3A shRNA1 (shA1) and shRNA3 (shA3) exhibited great knockdown efficacy. ATAD3A shRNA1 and 3 were thus used through the study. (B) MEF cells were stained with anti-Tom20 to indicate mitochondrial network. The boxed area was enlarged and shown in the right down corner in each of images. Scale bar: 10 µm. (C) The quantitation of the number of cells with fragmented mitochondria. ~200 cells/group were analyzed. The data are mean ± SEM of 3 independent experiments, one-way ANOVA with Tukey's *post-hoc* test.

Supplementary Figure 5

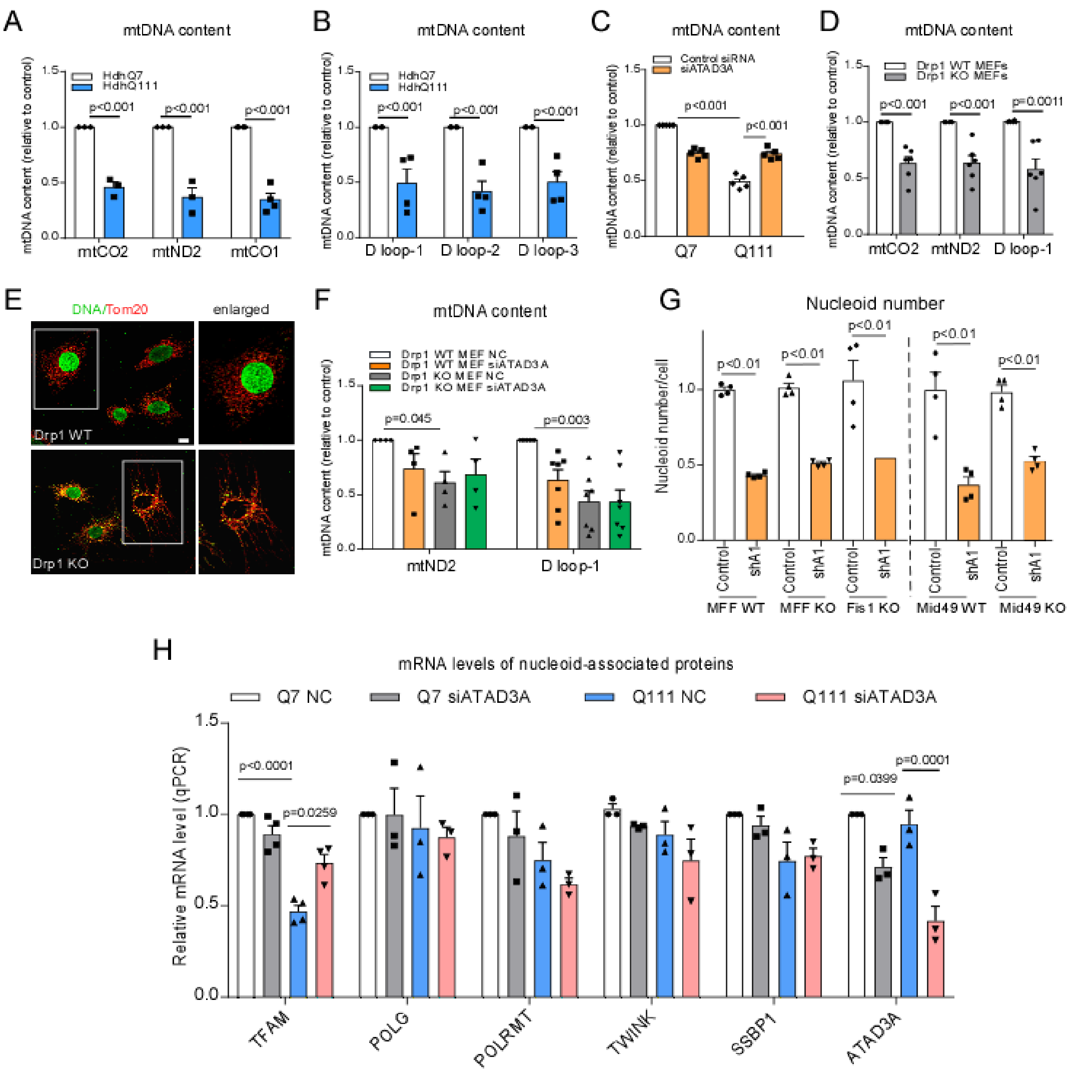

Supplementary Figure 5: Analysis of mtDNA content in cells. Mitochondrial DNA content was analyzed by qPCR in HdhQ7 and HdhQ111 striatal cells using primers of mtCO2, mtND2 and mtCO1 (A), and primers from D-loop region of mtDNA (B). (C) HdhQ7 and HdhQ111 cells were transfected with control siRNA or ATAD3A siRNA for 3 days. mtDNA content was analyzed by qPCR with mtND2 primers. (D) mtDNA content was compared between Drp1 WT and KO MEFs using qPCR with primers of mtCO2, mtND2 and D-loop. (E) Drp1 WT and KO MEFs were stained with anti-Tom20 (red) and anti-DNA (green) antibodies. The colocalization of DNA/Tom20 was imaged. Scale bar: 10 μm. (F) Drp1 WT and KO MEF cells were transfected with control siRNA or ATAD3A siRNA for 4 days, and then the total DNA was isolated for the mtDNA content analysis by qPCR. (G) Cells at the indicated groups were stained with anti-Tom20 and anti-DNA antibodies. The co-localization of DNA and Tom20 was analyzed by confocal microscopy (40X oil lens). The number of nucleoids immunopositive for both anti-DNA and anti-Tom20 was quantitated by NIH Image J software. (H) HdhQ7 and HdhQ111 striatal cells were transfected with control siRNA or ATAD3A siRNA for 3 days. The mRNA levels of nucleoid components including TFAM, POLG, POLRMT, TWINK and SSBP1 were analyzed by qPCR. All the data are mean ± SEM of at least 3 independent experiments, two-way ANOVA with Tukey's *post-hoc* test.

Supplementary Figure 6

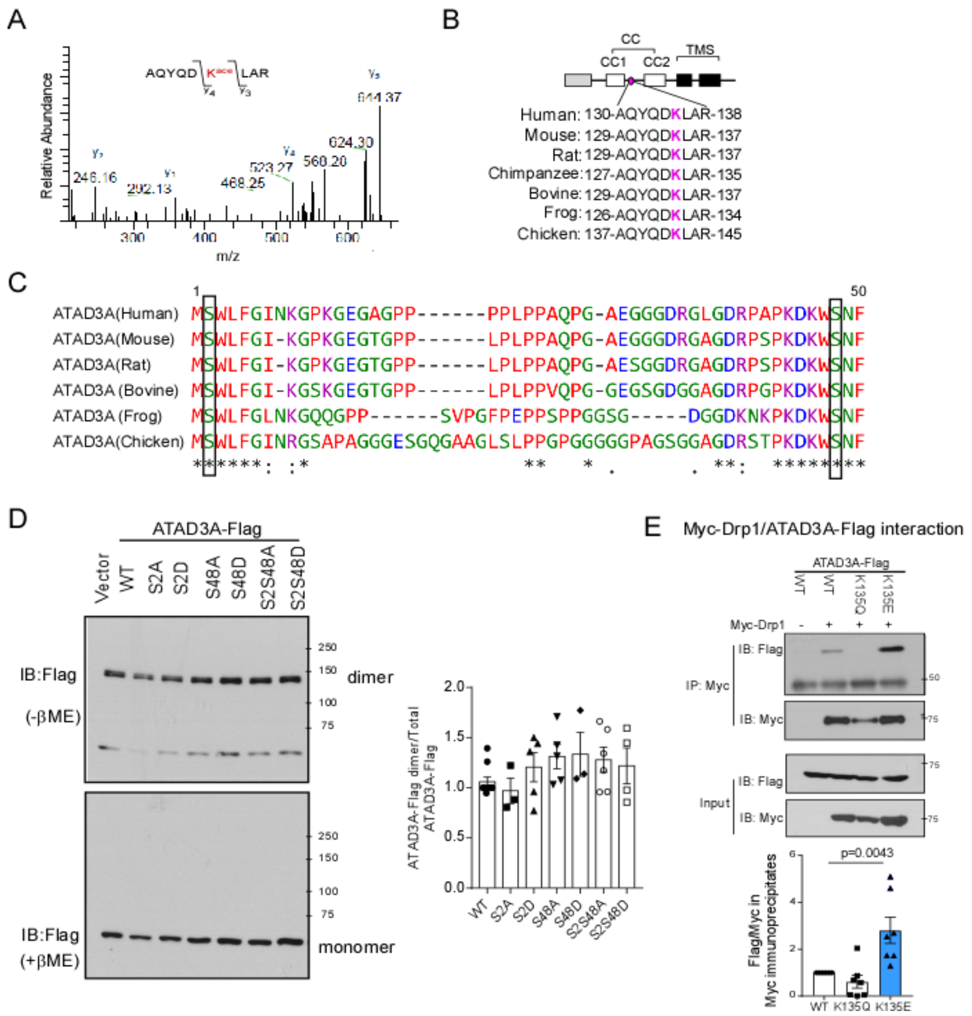

Supplementary Figure 6: ATAD3A K mutants. Total cell lysates of HdhQ7 and HdhQ111 cells were IP with anti-ATAD3A antibodies followed by LC-MS analysis. (A) LC-MS/MS spectra of K^{ace} in peptide 129-AQYQDKLAR-137 in mouse HdhQ7 cells (K^{ace}: m/z 567.7987). (B) The peptide sequence containing K^{ace} is conserved among species. (C) Sequence alignment indicated two conserved serine sites (S2 and S48) within the first 50 amino acids. Computation prediction indicates S48 is the putative phosphorylated site. (D) Serine site mutagenesis was performed. The serine 2 or 48 was replaced to either Alanine (phosphor-deficient, S2A, S48A, S2S48A) or Aspartate (phosphor-mimetic, S2D, S48D, S2S48D). HEK293 cells were transfected with phosphor-mutants of ATAD3A as indicated for 48 hours. ATAD3A dimerization was determined by western blot analysis in the absence of β-ME. No significant ATAD3A dimerization was observed among cells expressing various serine phosphorylated mutants. (E) HEK293 cells were transfected with Myc-Drp1 and ATAD3A-Flag WT or K mutants for 48 hours. Immunoprecipitation with anti-Myc antibody followed by western blot with the indicated antibodies was performed to determine Myc-Drp1 and ATAD3A-Flag interaction. Histogram shows the quantitation of relative density of Flag/Myc in Myc immunoprecipitates. All data are mean ± SEM of at least 3 independent experiments, one-way ANOVA with Tukey's *post-hoc* test.

Supplementary Figure 7

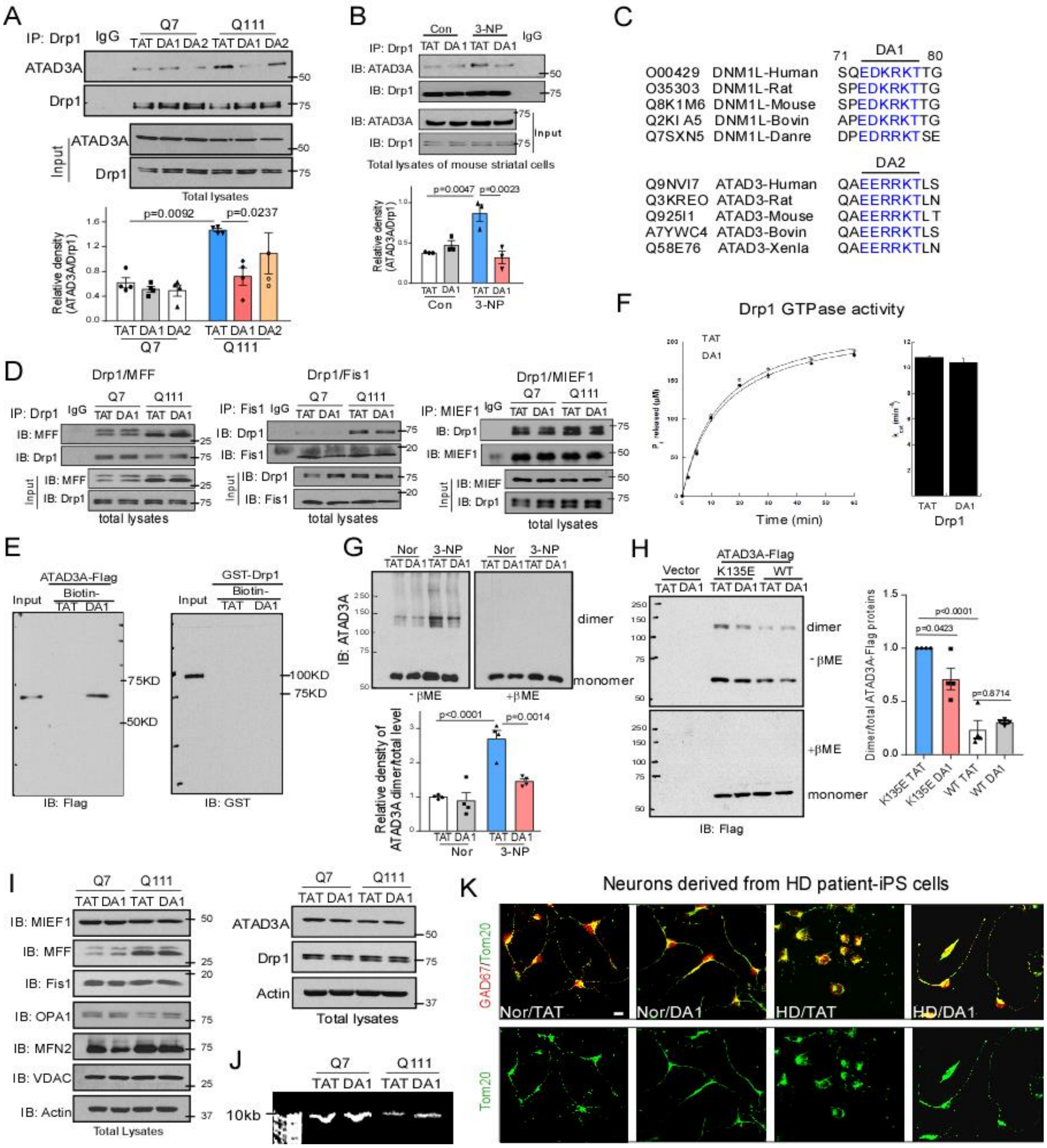

Supplementary Figure 7: Effects of DA1 on mitochondrial dynamics. (A) cells were treated with TAT or DA1 or DA2 (1 µM/day for 4 days, each). IP analysis was performed. Histogram: quantitation of ATAD3A level in Drp1 immunoprecipitates. 4 independent experiments, two-way ANOVA with Tukey's *post-hoc* test. (B) Wildtype mouse striatal cells were treated with TAT or DA1 (1 µM) overnight followed by exposure to 3-NP (5mM for 4 hours). IP analysis was performed. Histogram: the quantitation of ATAD3A levels in Drp1-immuniprecipates. 3 independent experiments. (C) Sequence alignment of DA1 and DA2 through species. (D) cells were treated with DA1 or TAT (1 µM/day for 4 days), followed by incubation with crosslinker 1mM DSP (left, right) or 1% FA (middle). IP analysis was performed. Blots are representative of 3 independent experiments. (E) Biotin-conjugated DA1 or TAT (10 µM, each) was incubated with either ATAD3A-Flag or GST-Drp1 proteins. Immunoprecipitates were analyzed by WB with the indicated antibodies. Shown blots are representative of 3 independent experiments. (F) Left: Time course of Drp1 GTP hydrolysis plotted as a function of inorganic phosphate (Pi) released over time. Right: The turnover number (k_{cat}) of initial Drp1 GTP hydrolysis in the absence and presence of DA1 is plotted. 3 independent experiment. (G) Neuo2A cells were treated with TAT or DA1 (1 µM) overnight following exposure of 3-NP (5mM) for 7 hours. ATAD3A dimerization was determined. Histogram: the relative density of ATAD3A dimer versus the total level of

ATAD3A in the presence of β-ME. 4 independent experiments. (H) HEK293 cells were expressed ATAD3A WT or K135E mutant. ATAD3A dimerization was determined. Histogram: the relative density of ATAD3A dimer versus the total level of ATAD3A in the presence of β-ME. 4 independent experiments. (I) The total protein levels of mitochondrial dynamics proteins were determined by WB. Shown blots are representative of 2 independent experiments. (J) Representative DNA agarose gel of amplification of the 10kb mtDNA fragment for Fig. 6F. (K) The images show a cluster of neurons for Fig. 7A. Scale bar: 10 µm. Data are mean ± SEM. One-way ANOVA with Tukey's *post-hoc* test for B, G, H.

Supplementary Figure 8

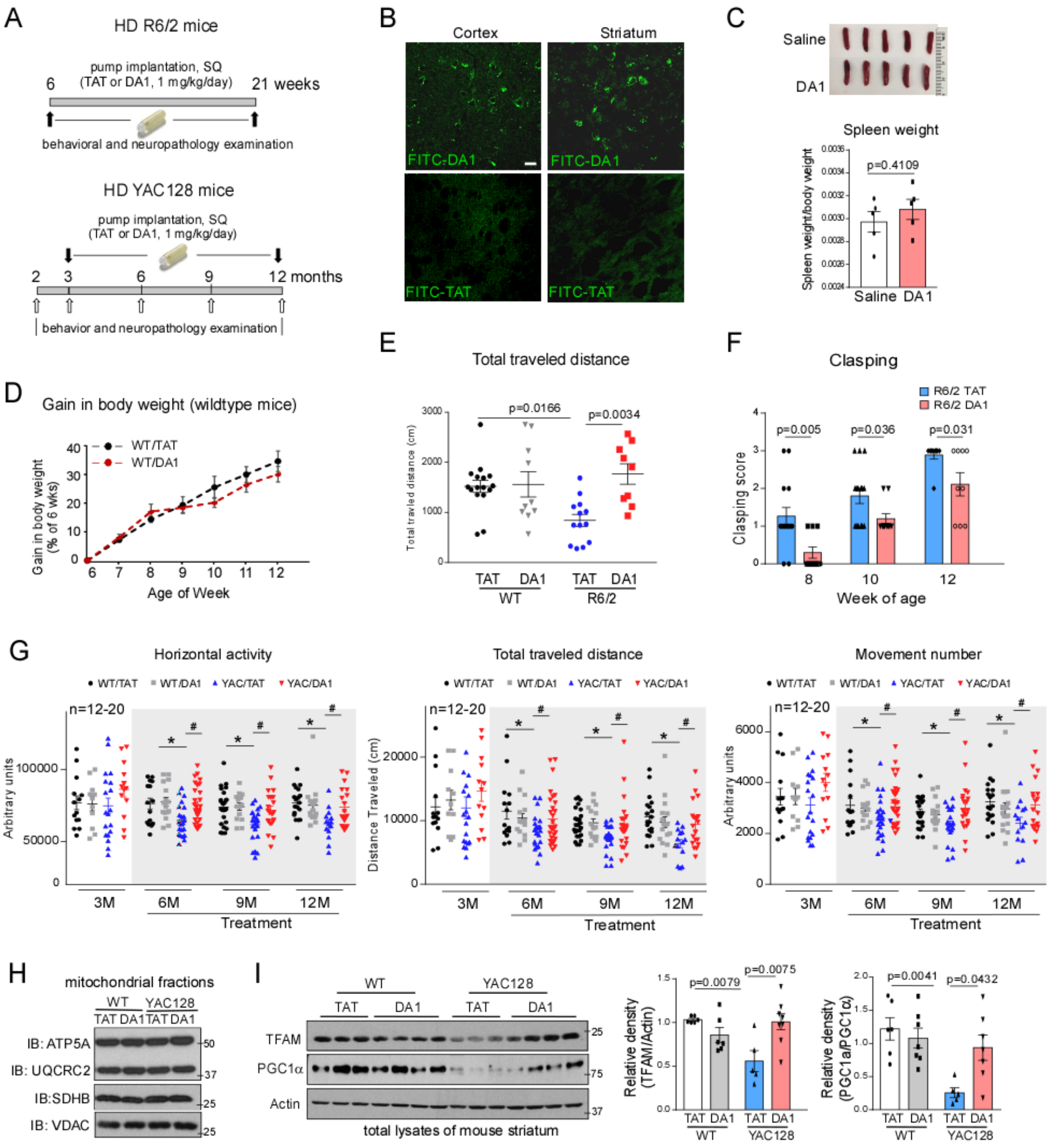

Supplementary Figure 8: Effects of DA1 treatment in R6/2 and YAC128 HD mice. (A) Timeline and administration avenue of DA1 treatment in HD R6/2 (upper) and YAC128 (lower) mice. (B) FITC-conjugated DA1 or TAT (1 mg/kg, each) was i.p. injected in wildtype mice for 1 day. Mouse brains were harvested and sectioned (10 µm). FITC-conjugated DA1 was visualized by microscope. Scale bar: 10 µm. (C) Wildtype mice were treated with DA1 (1 mg/kg/day) or saline for 30 days using Alzet mini pumps. Thirty days of DA1 treatment had no effects on spleen morphology and relative weight (spleen/body weight). n=5 mice/group. (D) Body weight of wildtype mice treated with TAT or DA1 (1 mg/kg/day) was recorded from the age of 6 to 12 weeks. n=10 mice/group. (E) One hour of overall movement activity in R6/2 and wildtype mice was determined by locomotion activity chamber at the age

of 12 weeks (WT/TAT: 16 mice; WT/DA1: 10 mice; R6/2/TAT: 13 mice; R6/2/DA1: 9 mice). Shown is total traveled distance of mice. One-way ANOVA with Tukey's *post-hoc* test. (F) Hindlimb clasping was assessed with the tail suspension test once a week from the ages of 8 to 12 weeks (n=10-15 mice/group). Multiple t test. (G) YAC128 and wildtype mice were treated with the TAT or DA1 (1 mg/kg/day) from the age of 3 to 12 months. A locomotion activity chamber was used to monitor 24 hours of general motility of YAC128 and wildtype mice at the indicated age (n=12-20 mice/group). Shown are horizontal activity, total traveled distance and movement number of mice. #, $p<0.05$ vs. HD mice treated with TAT; *, $p<0.05$ vs. wildtype mice treated with TAT, two-way ANOVA with Tukey's *post-hoc* test. (H) nuclear-encoded mitochondrial respiratory related proteins were analyzed with striatal mitochondrial lysates of YAC128 and wildtype mice (6 months old) by WB with the indicate antibodies. Shown blots were from three independent experiments. (I) TFAM and PGC1α in total striatal lysates of YAC128 mice (12 months old) were analyzed by WB. Actin was used as a loading control. n=6 mice/group, one-way ANOVA with Tukey's *post-hoc* test. Data are mean ± SEM.

Supplementary Figure 9

Figure 1 (Zhao et al)

Fig 1C (Left)

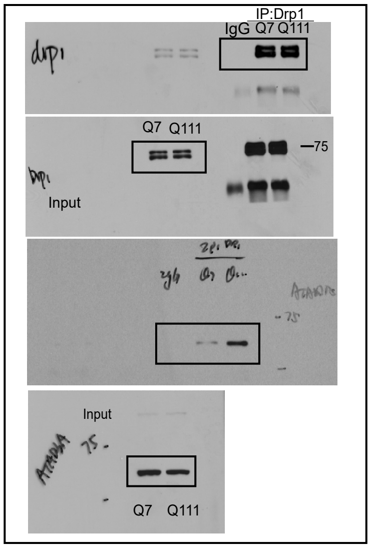

Fig 1C (Right)

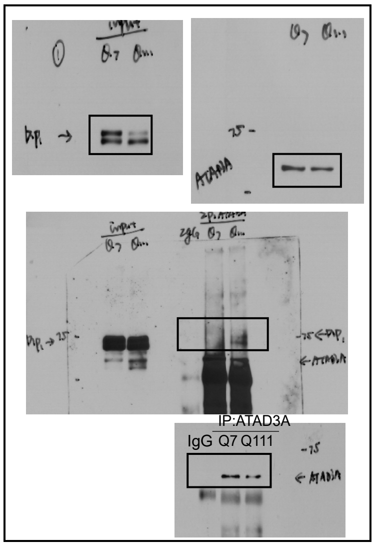

Fig 1D

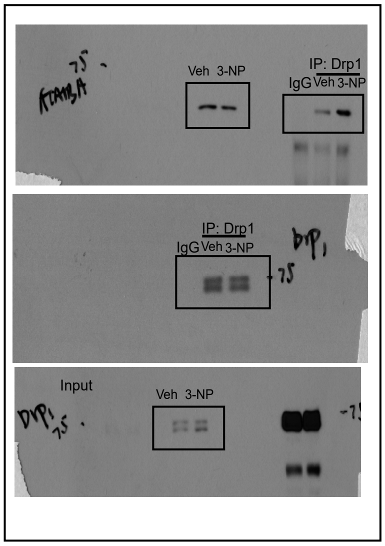

Fig 1F

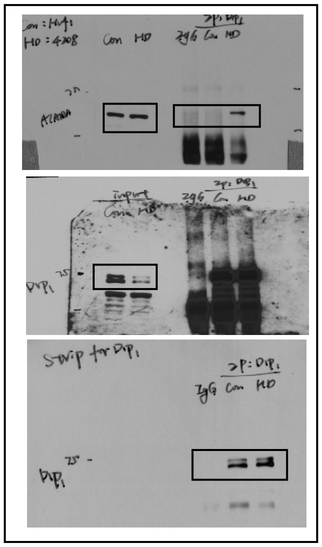

Fig 1E (Left)

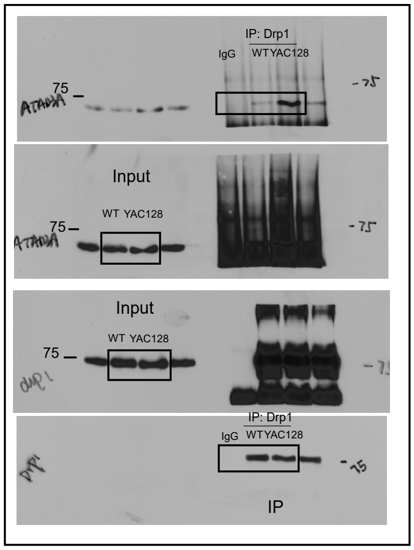

Fig 1E (Right)

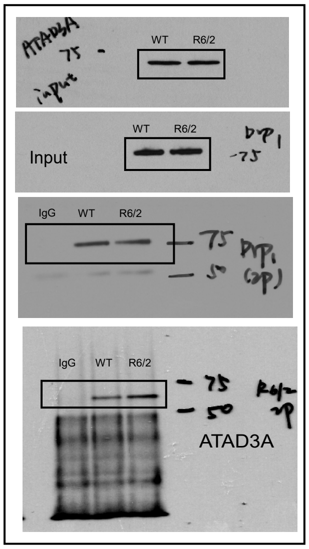

Fig 1G

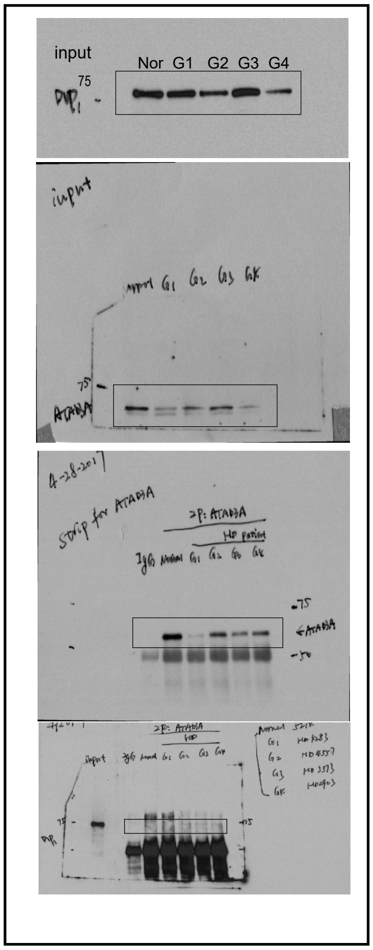

Fig 1H (Left)

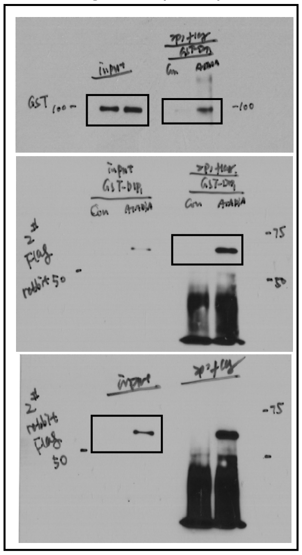

Fig 1H (Right)

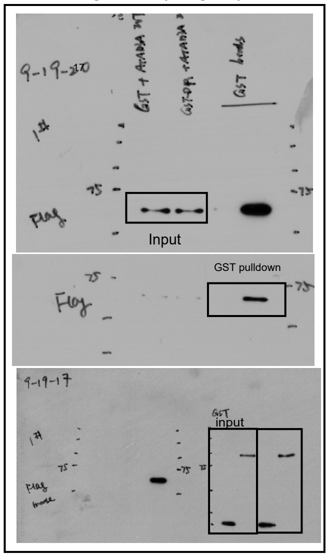

Figure 2 (Zhao et al)

Fig 2A

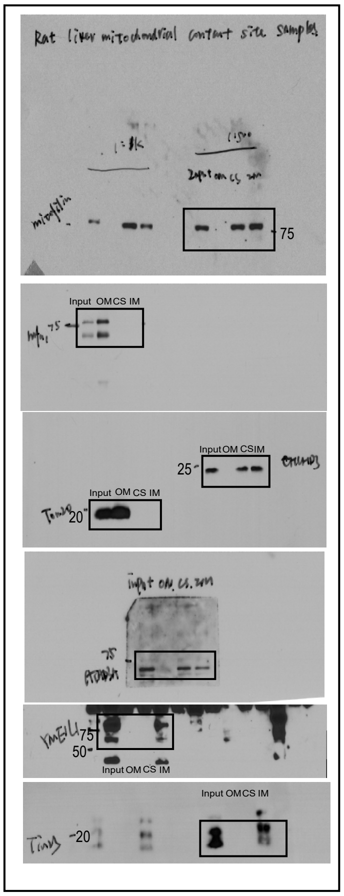

Fig 2D (Left)

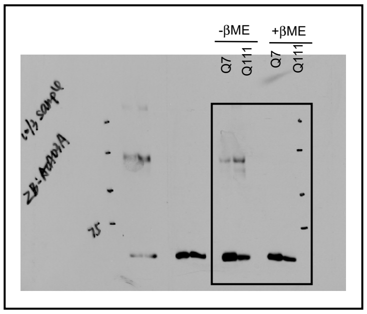

Fig 2D (Right)

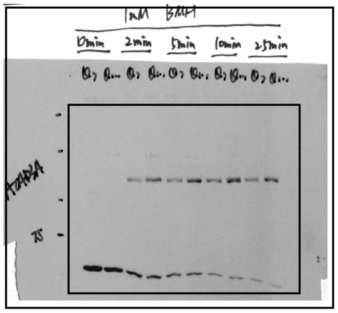

Fig 2E

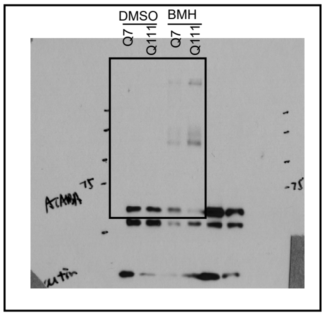

Fig 2F

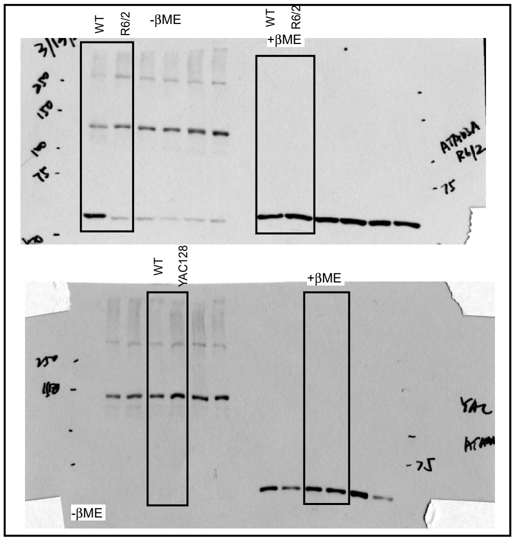

Fig 2G

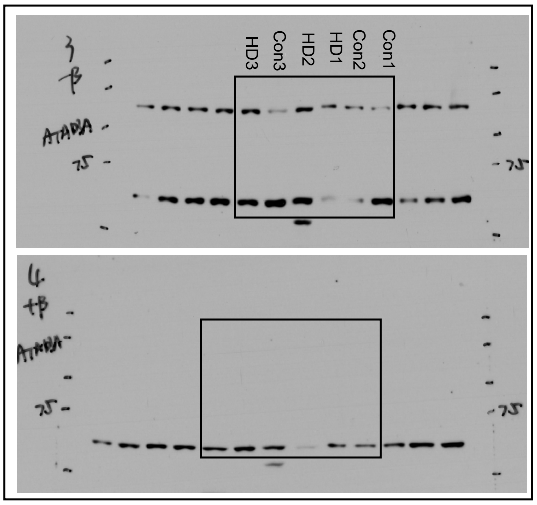

Fig 2H (Left)

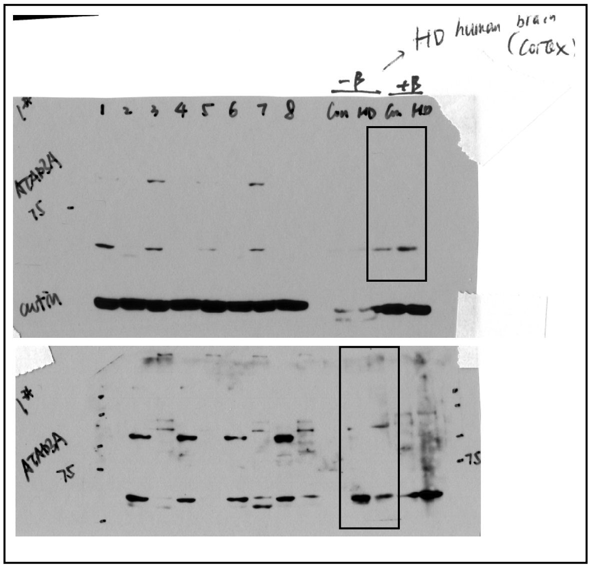

Fig 2H (Right)

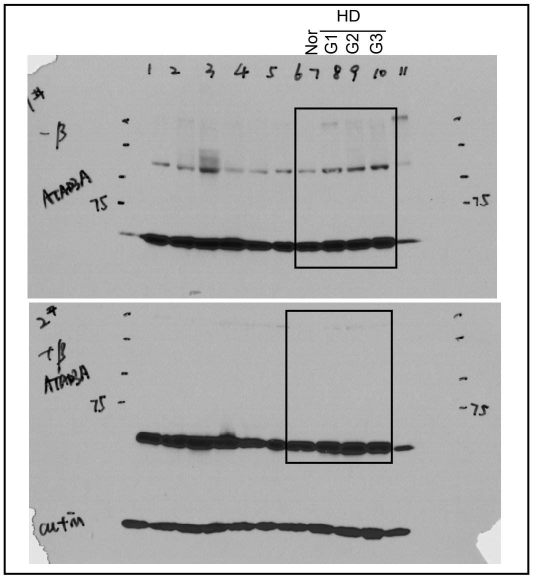

Figure 3 (Zhao et al)

Fig 3A-Top

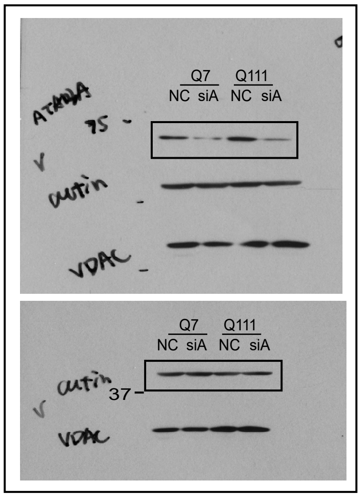

Fig 3A-Bottom

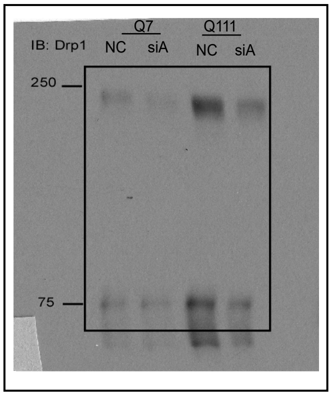

Fig 3H-Left

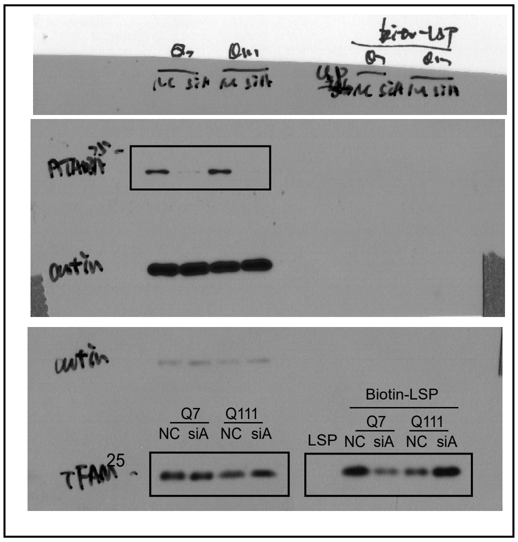

Fig 3B-Top

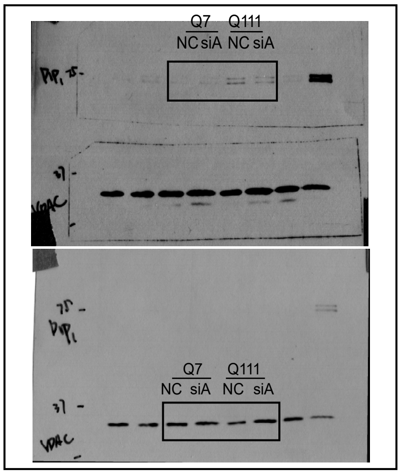

Fig 3B-Bottom

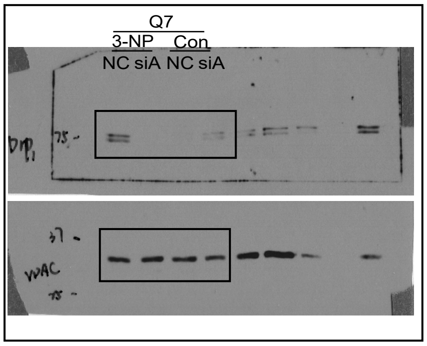

Fig 3H-Right

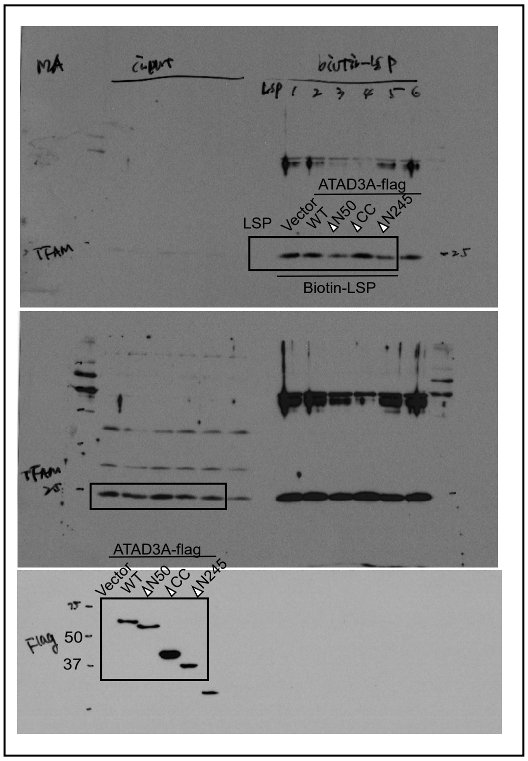

Fig 3E

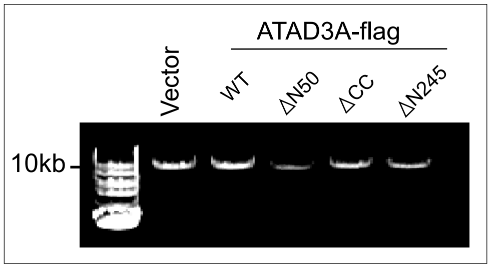

Figure 4 (Zhao et al)

Fig4A (left)

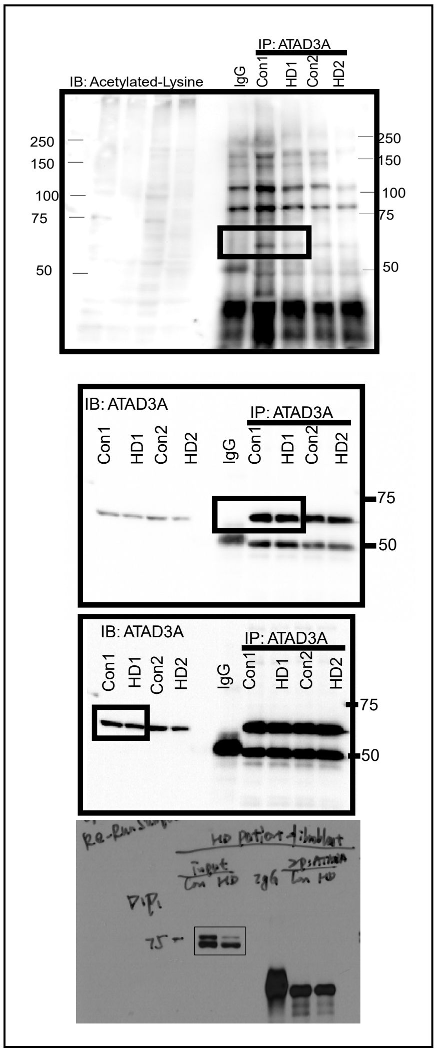

Fig4A (right)

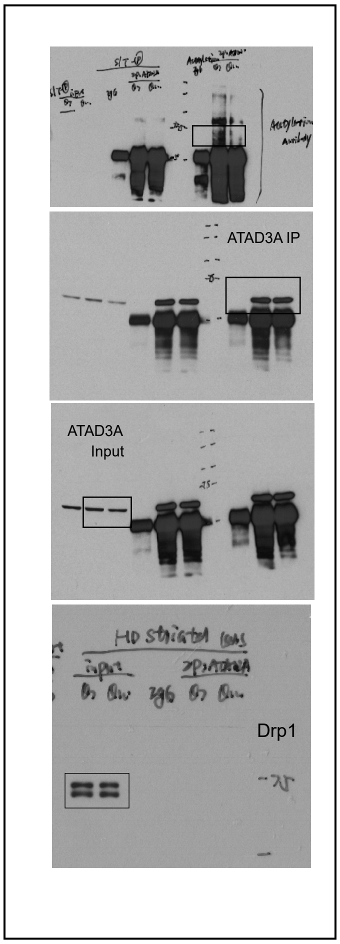

Fig4C

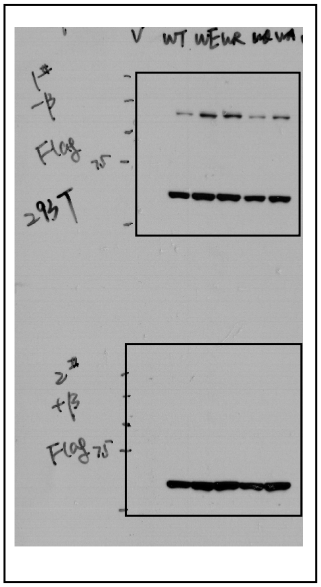

Fig4D

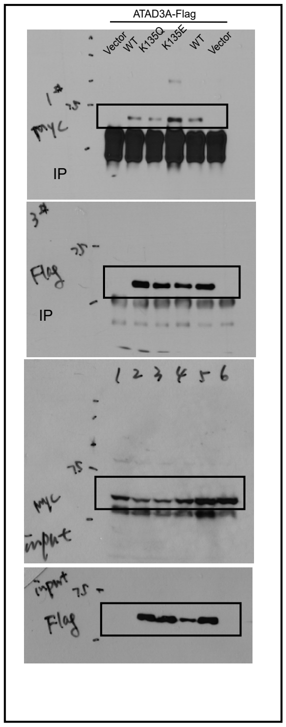

Fig4E

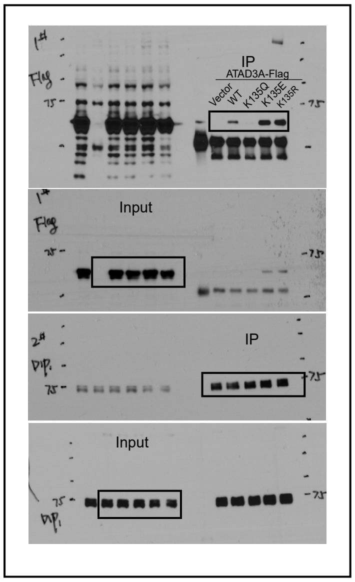

Fig4G

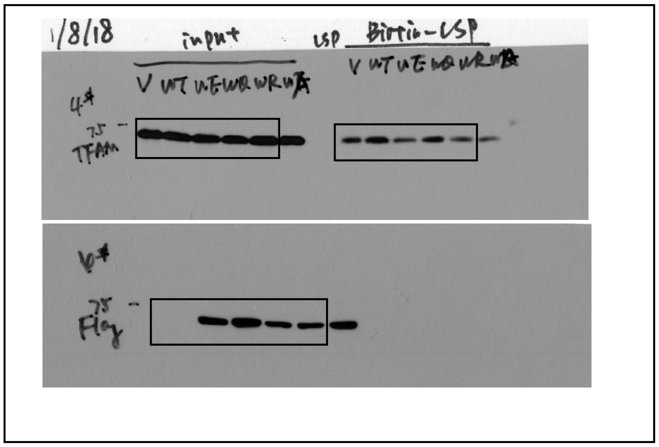

Figure 5 (Zhao et al)

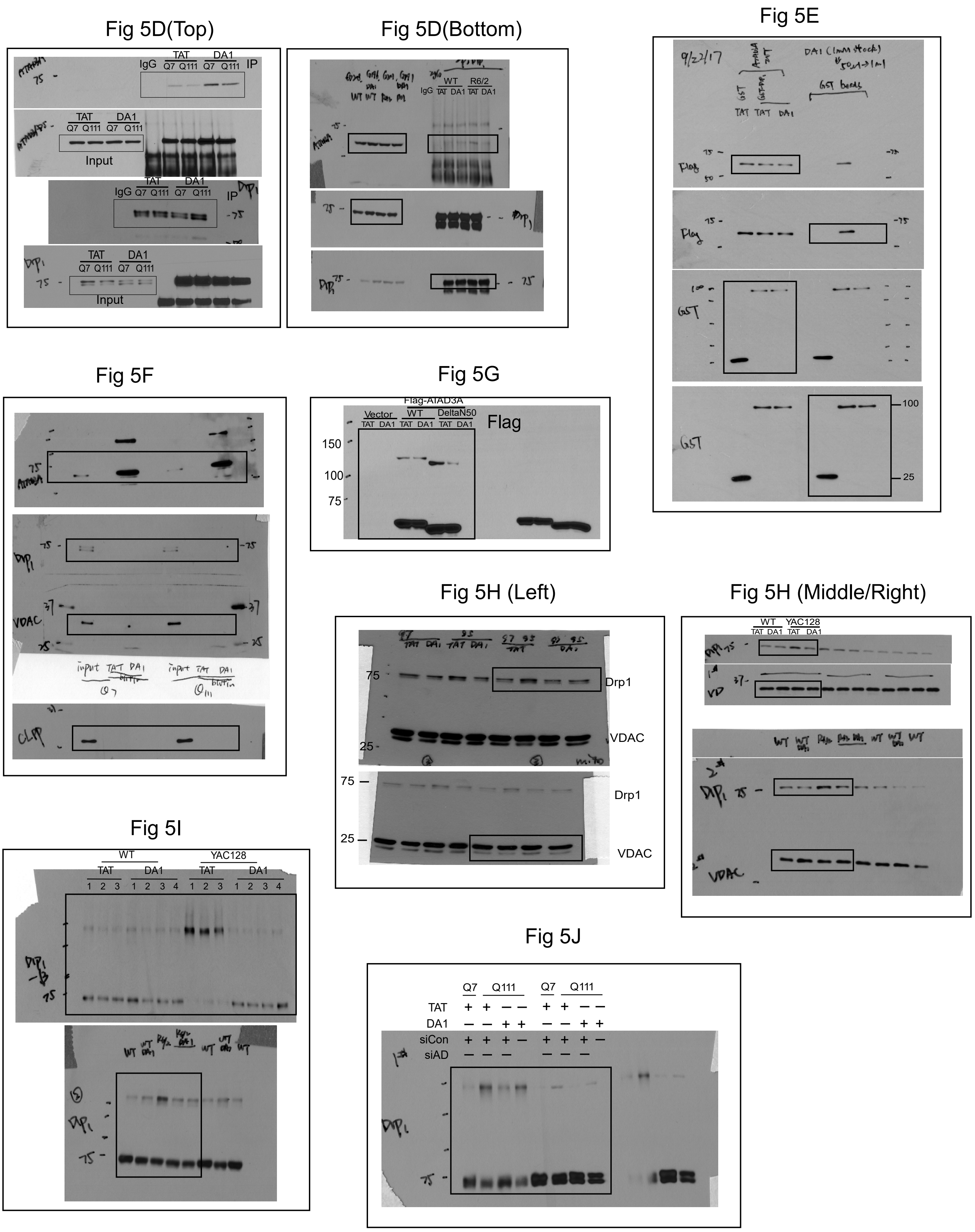

Figure 6 (Zhao et al)

Fig 6B

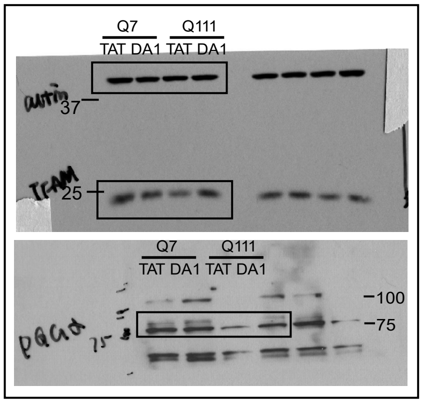

Fig 6C

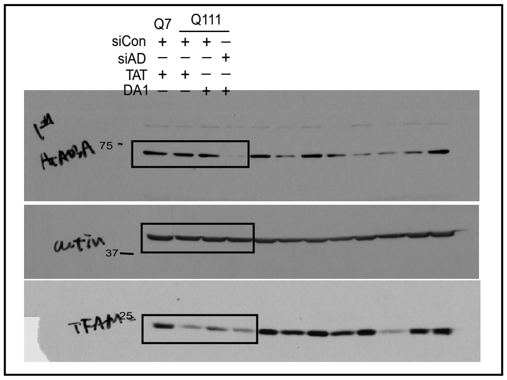

Fig 6E

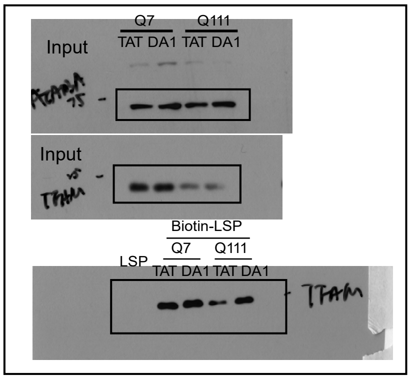

Fig 6D

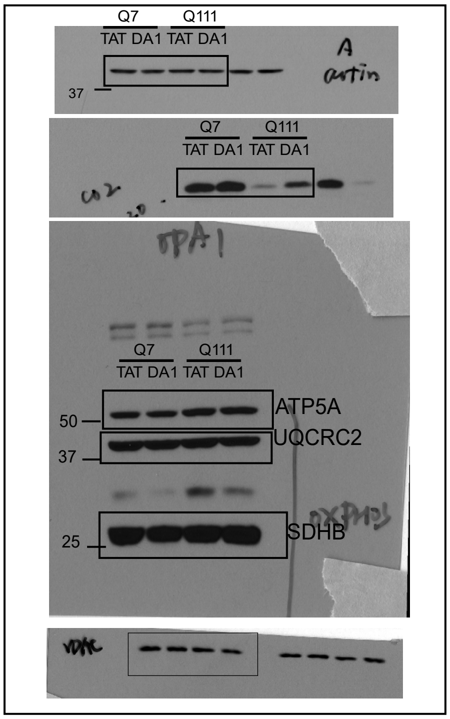

Figure 7 (Zhao et al)

Fig 7L

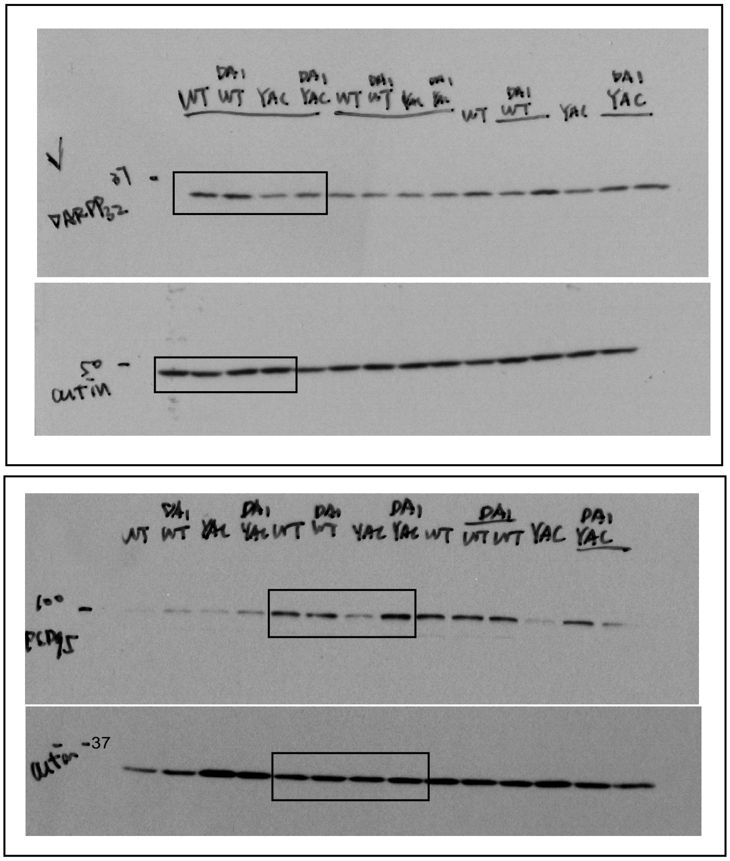

Figure 8 (Zhao et al)

Fig 8A

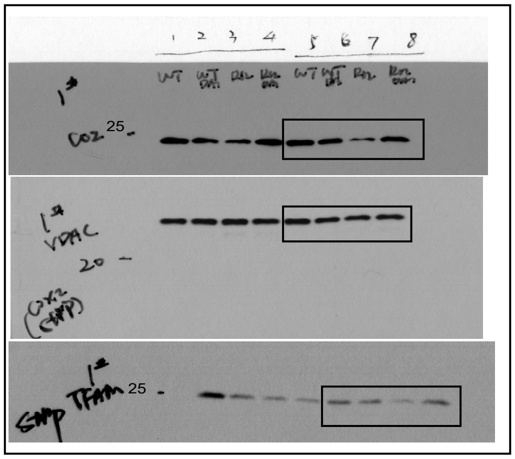

Fig 8B

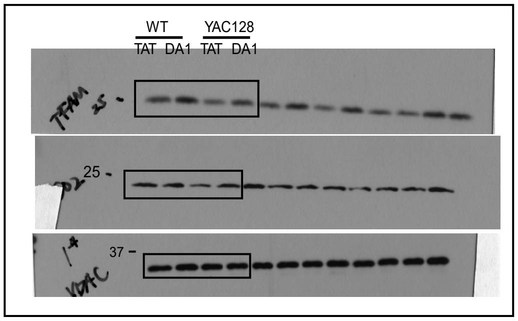

Supplementary Figure 1 (Zhao et al)

Sup Fig 1C (Left)

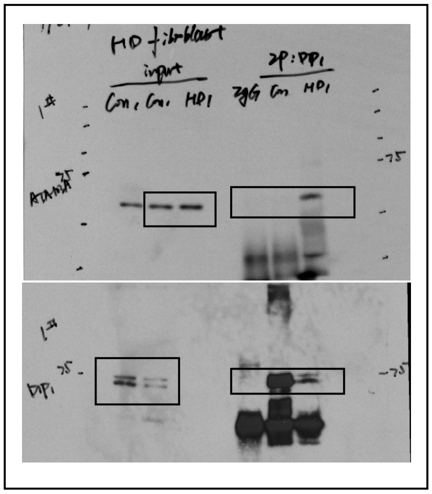

Sup Fig 1C (Right)

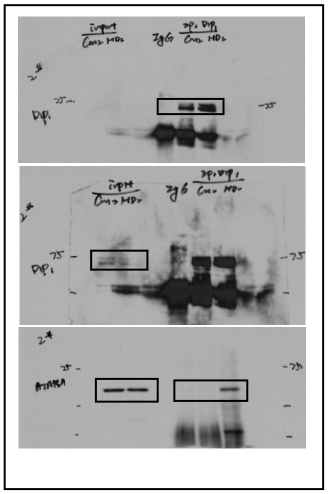

Sup Fig 1D(Left)

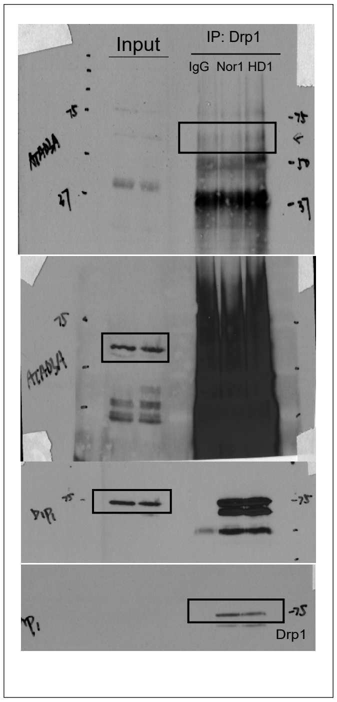

Sup Fig 1D(Right)

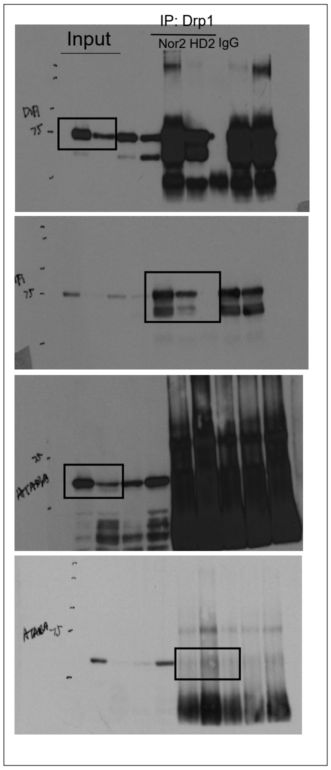

Sup Fig 1E

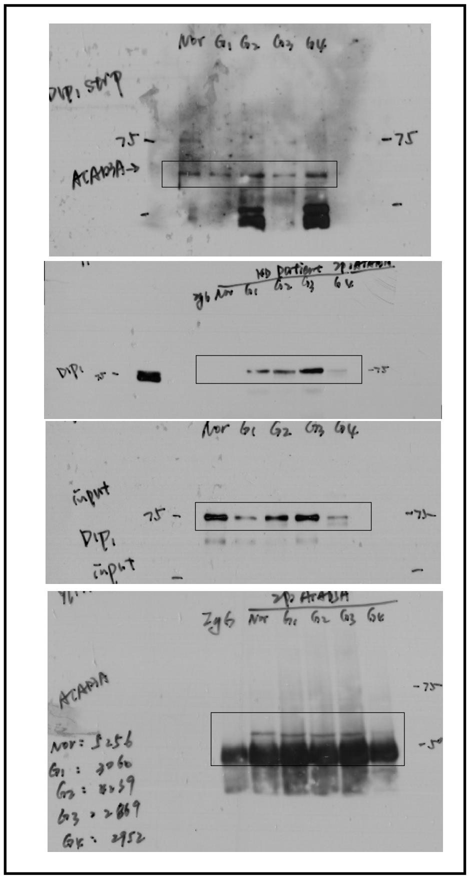

Sup Fig 1G(Left)

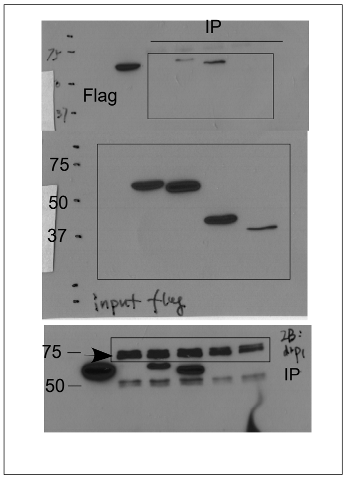

Sup Fig 1G(Right)

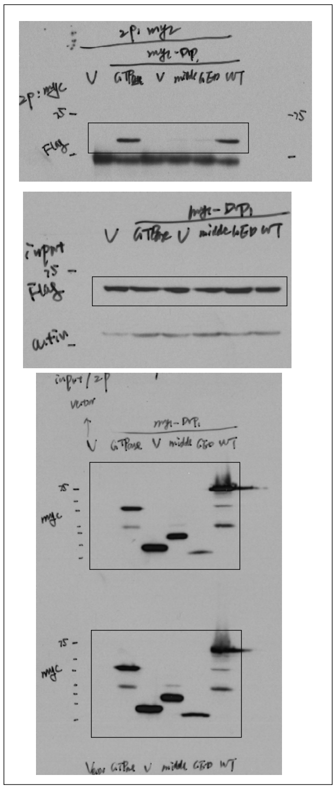

Supplementary Figure 2 (Zhao et al)

Sup Fig2B

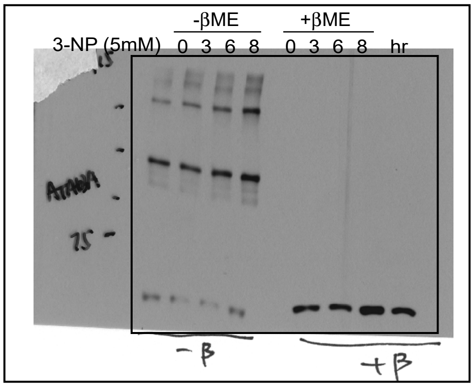

Sup Fig2C

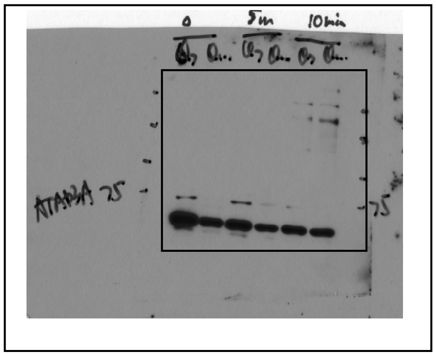

Sup Fig2D

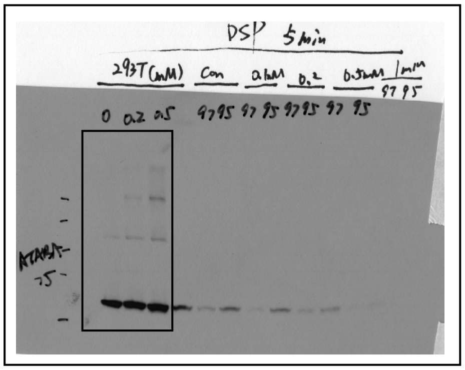

Sup Fig2E

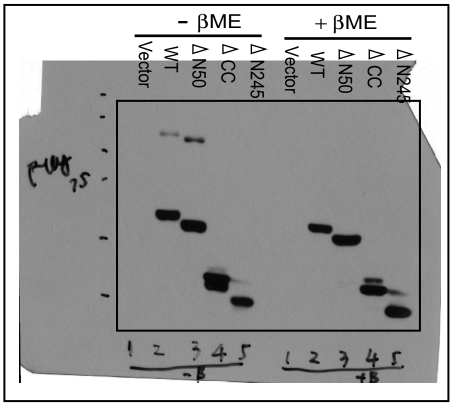

Sup Fig2F

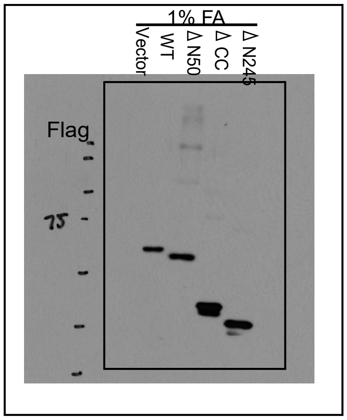

Sup Fig2I(Left 1)

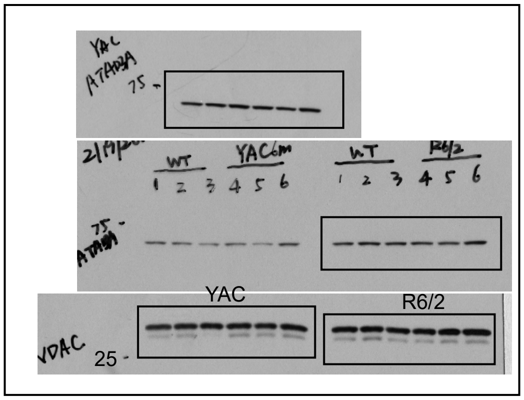

Sup Fig2G

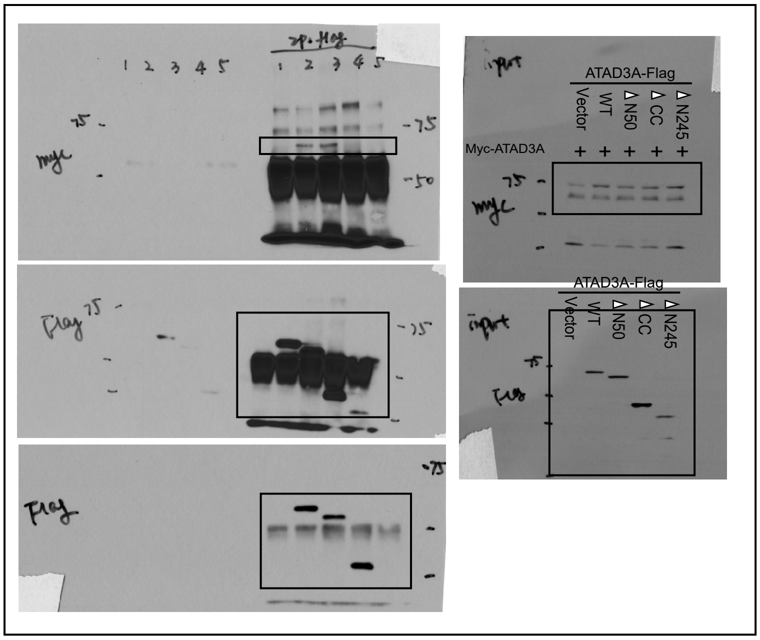

Sup Fig2I (Left 2-YAC 12months)

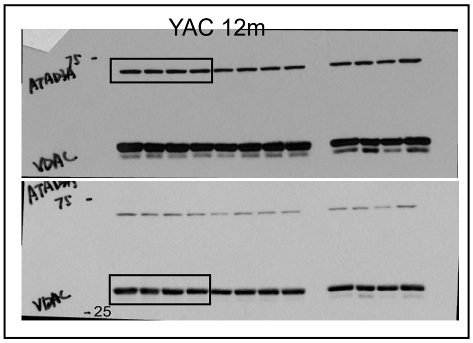

Sup Fig2I (Right)

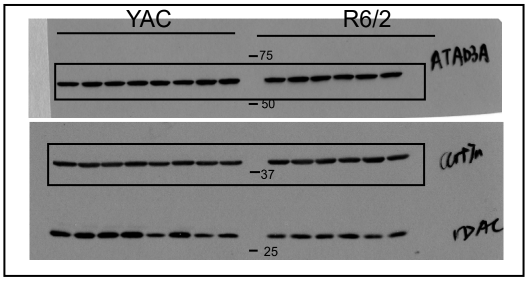

Supplementary Figure 3 (Zhao et al)

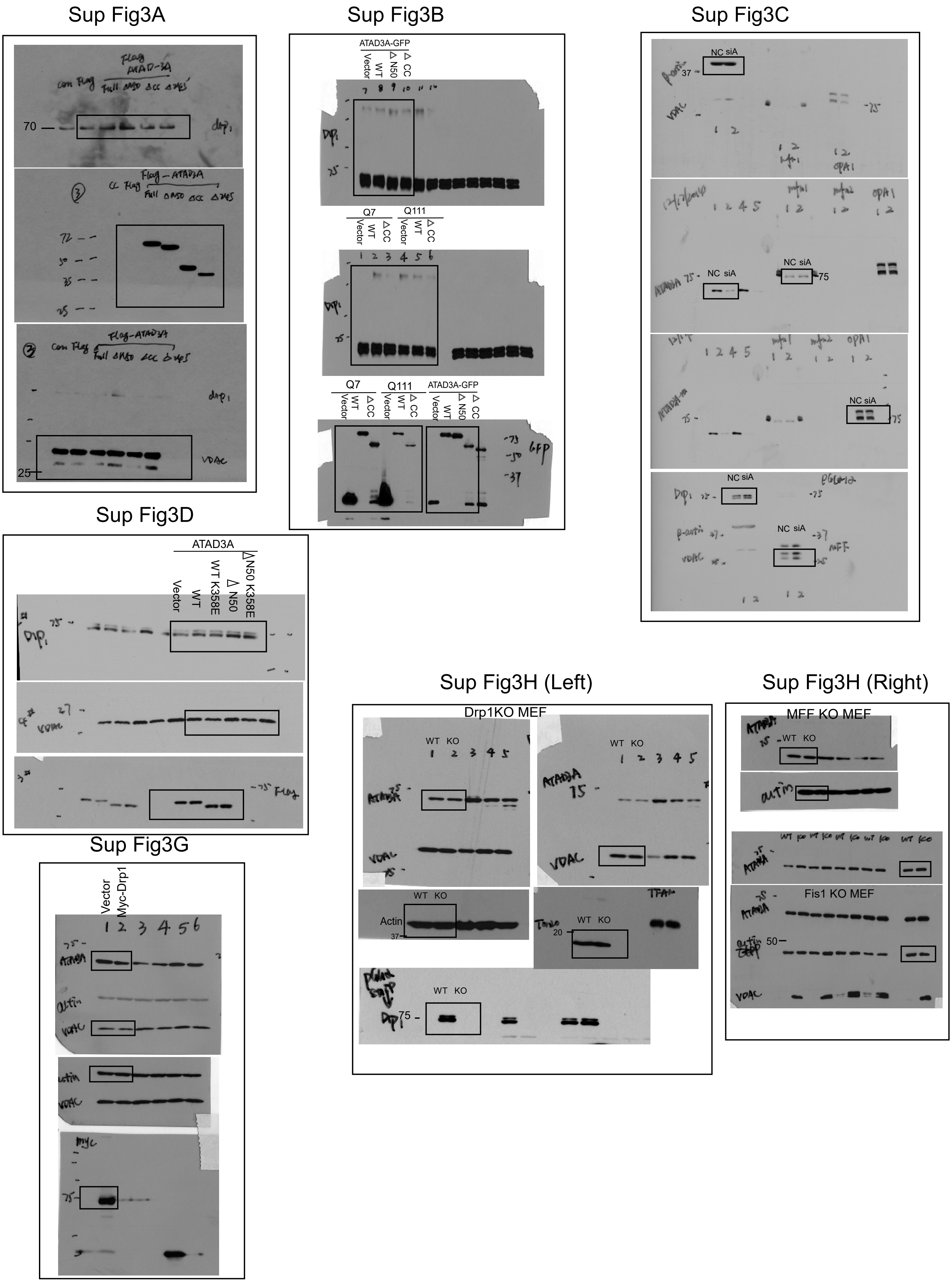

Supplementary Figure 4 (Zhao et al)

Sup Fig 4A

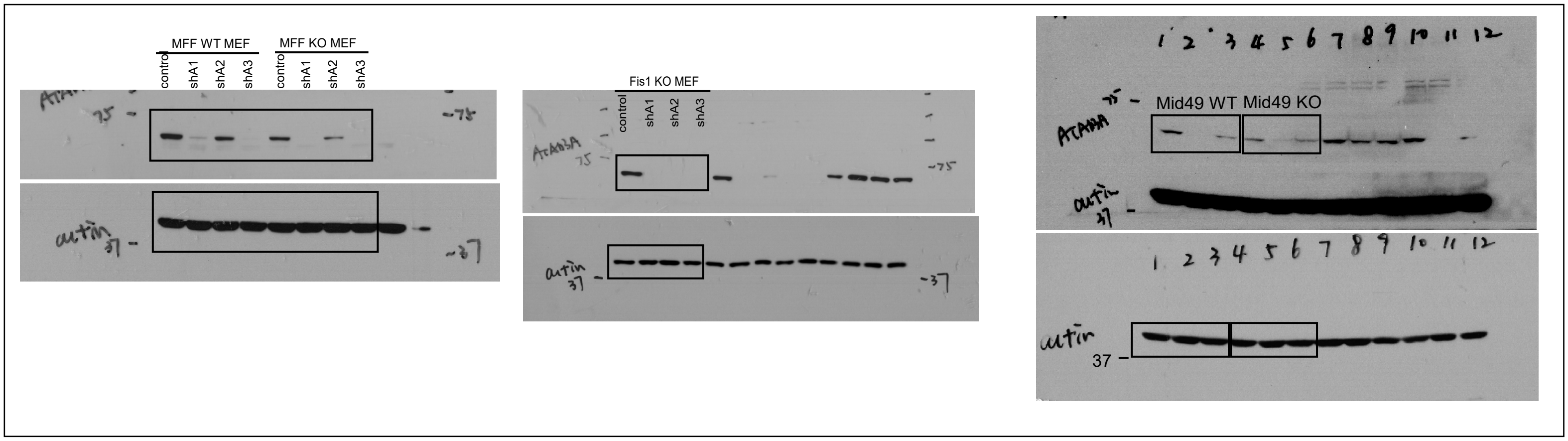

Supplementary Figure 6 (Zhao et al)

Sup Fig 6D

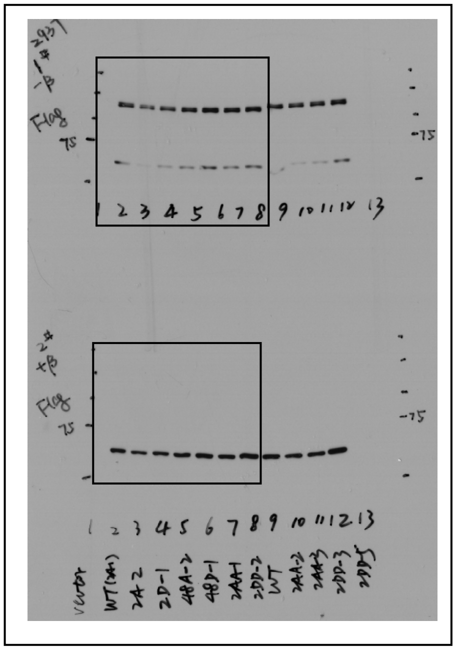

Sup Fig 6E

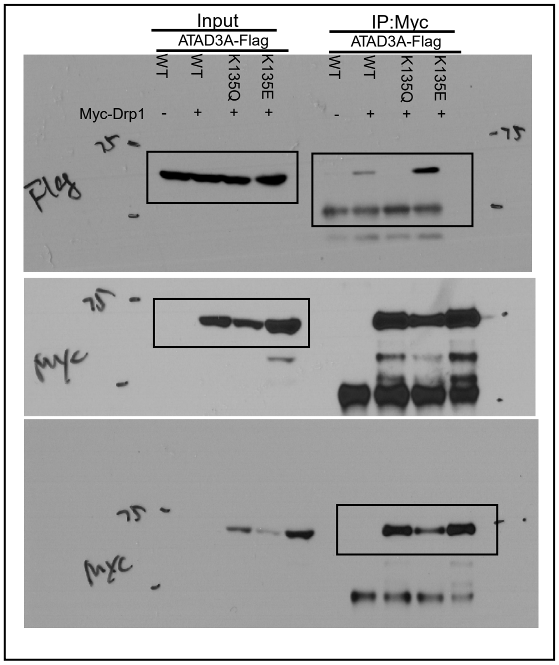

Supplementary Figure 7 (Zhao et al)

Sup Fig 7A

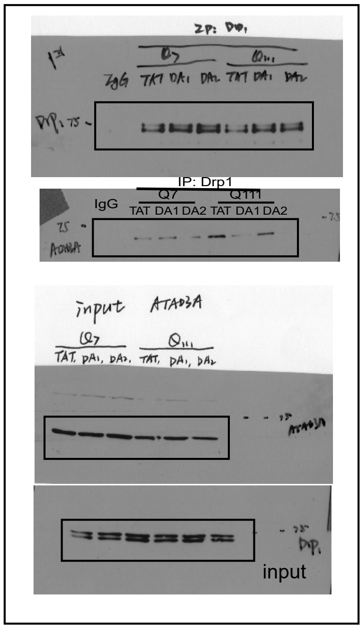

Sup Fig 7B

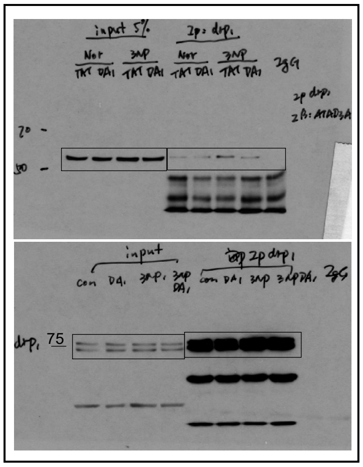

Sup Fig 7E

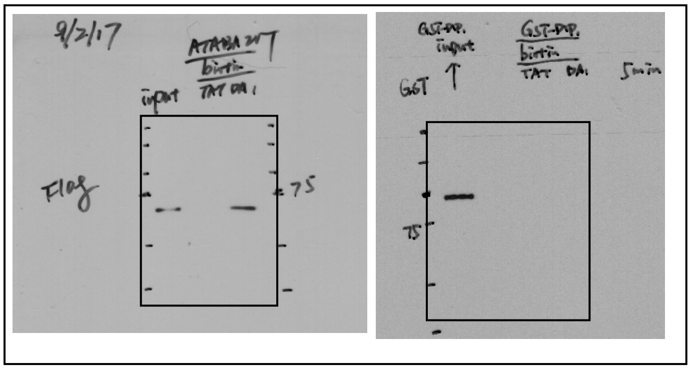

Sup Fig 7G

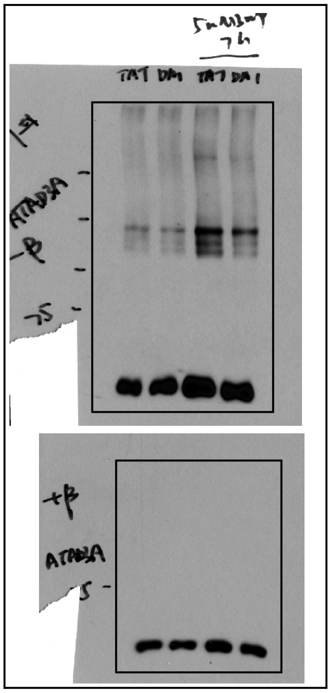

Sup Fig 7D (Left)

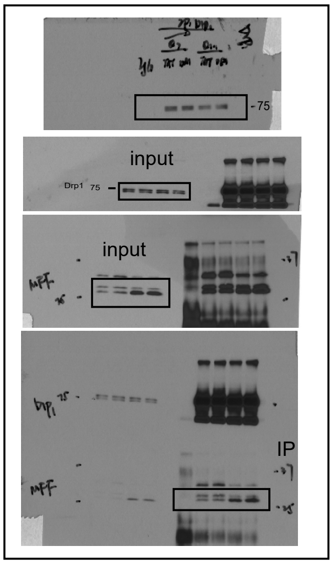

Sup Fig 7D (Middle)

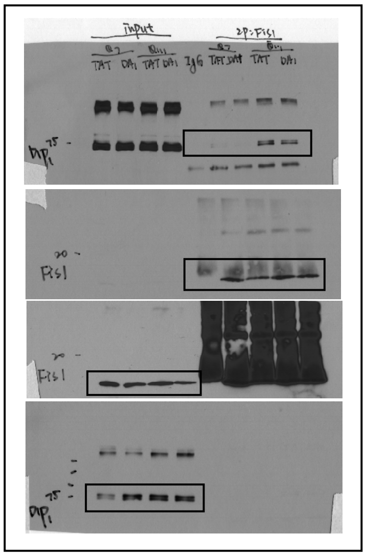

Sup Fig 7D (Right)

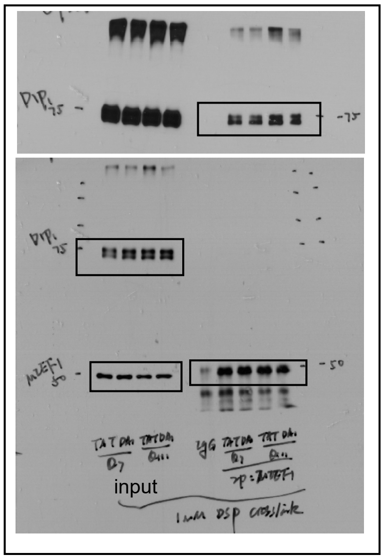

Sup Fig 7H

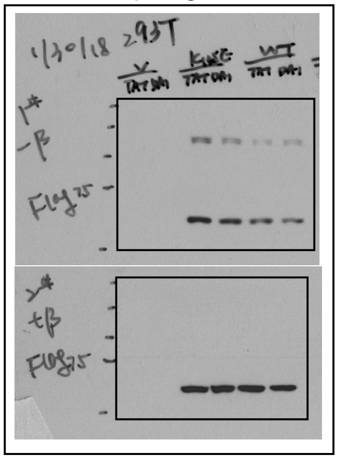

Sup Fig 7I (Left)

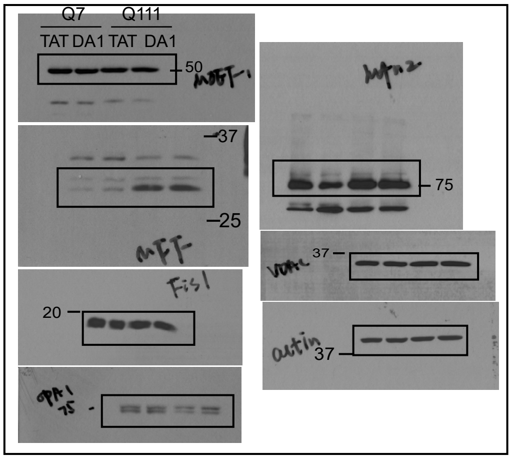

Sup Fig 7I (Right)

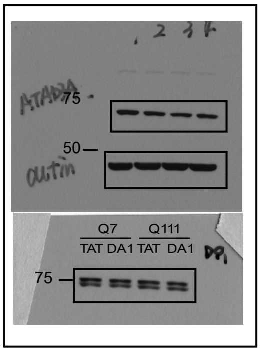

Sup Fig 7J

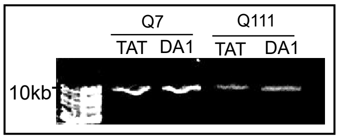

Supplementary Figure 8 (Zhao et al)

Sup Fig 8G

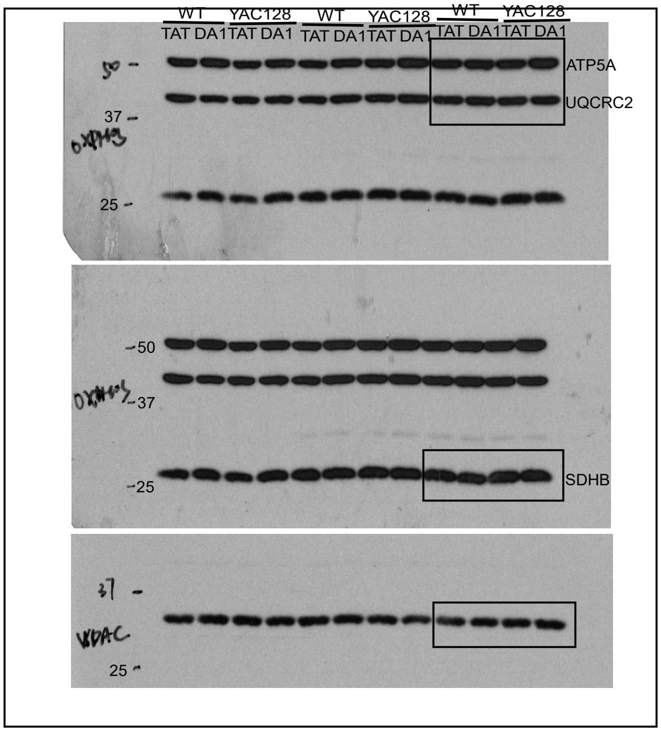

Sup Fig 8H

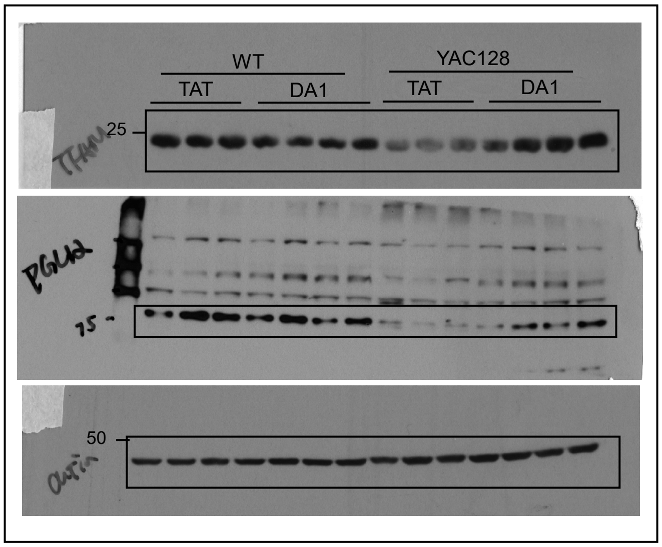

Supplement: Supplementary file 1 — Supplementary Information [file 41467_2019_9291_MOESM1_ESM.pdf]
